# Association of PIRCHE scores and allograft injury in kidney transplant recipients

**DOI:** 10.3389/fimmu.2026.1858108

**Published:** 2026-06-08

**Authors:** Miklos Z. Molnar, Kendon J. Holdaway, Divya Raghavan, Silviana Marineci, Suayp Oygen, Fruzsina Toth, Katalin Fornadi

**Affiliations:** 1 Transplant Institute, University of Rochester Medical Center, Rochester, NY, United States; 2 Division of Nephrology, Department of Medicine, University of Rochester, Rochester, NY, United States; 3 Department of Surgery, University of Rochester, Rochester, NY, United States; 4 Department of Internal Medicine, Division of Nephrology & Hypertension, Spencer Fox Eccles School of Medicine at the University of Utah, Salt Lake City, UT, United States; 5 Doctoral College, Theoretical and Translational Medicine Division, Semmelweis University, Budapest, Hungary; 6 Department of Surgery, Division of Transplantation and Advanced Hepatobiliary Surgery, Spencer Fox Eccles School of Medicine at the University of Utah, Salt Lake City, UT, United States

**Keywords:** antibody mediated rejection, donor derived cell free DNA, donor specific antibody, kidney transplantation, PIRCHE score, T cell mediated rejection

## Abstract

**Background:**

Early allograft injury during the first year after kidney transplantation is a major determinant of long-term graft survival. Predicted Indirectly ReCognizable HLA Epitopes (PIRCHE) quantify donor-derived human leucocyte antigen (HLA) peptides presented to recipient CD4^+^ T cells via the indirect pathway and may capture alloimmune risk beyond conventional HLA mismatch.

**Methods:**

In this single-center retrospective cohort study, we evaluated adult kidney transplant recipients transplanted between 2021 and 2024. Five-locus PIRCHE-T2 (T-cell epitope load) and PIRCHE-B (surface-accessible amino acid mismatch) scores were calculated using high-resolution HLA typing with imputation when necessary. The primary endpoint was a composite of post-transplant allograft injury within one year, including donor-specific antibody (DSA) development, histologic or molecular rejection, or elevation of donor-derived cell-free DNA (dd-cfDNA). Secondary endpoints were each component analyzed separately. Predictive performance was assessed using receiver operating characteristic analysis and compared to the only HLA A/B/DR antigen mismatch model, and associations were evaluated using Cox proportional hazards models.

**Results:**

Among 683 recipients, 250 (37%) experienced the primary endpoint. Higher five-locus PIRCHE-T2 and PIRCHE-B scores were significantly associated with the composite outcome [adjusted hazard ratio (HR)(95% confidence interval (CI)] _per point increase_ 1.009(1.004-1.014) and 1.043(1.027-1.060), and adjusted HR _low versus high risk_ 1.653(1.254-2.180) and 2.280(1.664-3.123), respectively). Both scores demonstrated modest discriminatory performance (area under the curve (AUC) 0.575–0.621). Higher PIRCHE scores were also independently associated with increased risk of *de novo* or recurrent DSA, dd-cfDNA elevation, and showed trends toward increased rejection risk. Sensitivity analyses incorporating six-locus PIRCHE-T2 (including DQA1) yielded consistent results. Compared with HLA A/B/DR antigen-mismatch models, PIRCHE-B scores improved prediction primarily for DSA, whereas PIRCHE-T2 did not add predictive value for outcomes.

**Conclusions:**

Higher PIRCHE scores are associated with increased risk of early alloimmune injury after kidney transplantation across multiple complementary biomarkers. Although discriminatory performance was modest, PIRCHE provides mechanistically grounded risk stratification and may complement existing immunologic assessment strategies.

## Introduction

Allograft injury during the first year after kidney transplantation is a critical determinant of long-term graft survival. Early injuries—including ischemia–reperfusion injury, acute cellular rejection, antibody-mediated rejection, and subclinical inflammation—initiate immune and non-immune pathways that promote chronic allograft dysfunction (1). These early insults accelerate structural damage, trigger maladaptive repair processes, and increase the risk of subsequent donor-specific antibody formation. Consequently, prevention, early detection, and mitigation of allograft injury in the first post-transplant year are central to improving long-term transplant outcomes.

PIRCHE (Predicted Indirectly ReCognizable HLA Epitopes) is a computational algorithm that estimates the repertoire of donor-derived HLA peptides that can be processed and presented by recipient human leucocyte antigen (HLA) class II molecules and recognized by recipient CD4^+^ T cells via the indirect pathway of allorecognition. The algorithm aggregates this predicted indirect alloreactivity into quantitative scores, including total and locus-specific metrics that reflect the recipient’s baseline immunologic exposure to donor HLA–derived epitopes. In the transplant context, higher PIRCHE scores are interpreted as indicative of an increased theoretical risk of CD4^+^ T cell–mediated alloimmune responses, particularly through provision of T cell help to B cells and subsequent donor-specific antibody (DSA) formation. Thus, PIRCHE scores provide a mechanistically grounded estimate of the burden of indirectly recognizable donor HLA epitopes presented on recipient HLA class II molecules and their potential to drive T cell–dependent humoral alloimmunity.

The PIRCHE-B (previously called Snow) matching algorithm is a comprehensive histocompatibility tool that integrates the Snowflake and Snowball methods to provide a detailed characterization of HLA protein surfaces (2, 3). It assesses compatibility by simultaneously evaluating allele-specific solvent accessibility and repeated local ellipsoid protrusion to determine if mismatched donor amino acids are physically accessible to recipient antibodies (2, 3). By identifying mismatches that exceed specific thresholds for both surface area and protrusion, the algorithm is hypothesized to more accurately predict immunological risk in solid-organ transplantation (2, 3). This integrated approach is available as a cloud-based web service designed to enhance the specificity of B-cell epitope matching compared to traditional molecular matching methods (2, 3).

Across multiple cohorts, higher class II PIRCHE-II scores—particularly DRB1- and DQB1-derived peptides—consistently predict an increased risk of *de novo* donor-specific antibodies (dnDSA), independent of conventional HLA mismatch and often independent of B-cell epitope load (4–11). Locus-specific analyses identify HLA-DQ as the dominant dnDSA target, supporting a key role for indirect CD4^+^ T-cell help (12, 13). Higher PIRCHE-II scores have also been independently associated with T cell–mediated rejection and antibody-mediated rejection, and predict progression of rejection and graft injury, underscoring their utility for immunologic risk stratification (7, 14–18).

Most of these studies focusing on dnDSA development or rejection used low resolution typing (4), while only a few used high resolution/allele level typing (6, 7, 12, 14). In addition, no previous kidney transplant study to date has measured and jointly analyzed PIRCHE scores using high-resolution/allele-level typing and comprehensive assessment of allograft injury using biomarkers such as elevation of donor-derived cell-free DNA (dd-cfDNA), DSA, histologic rejection and molecular rejection signatures using Molecular Microscope^®^ Diagnostic System (MMDx) platform (19).

In this retrospective, single-center study, we evaluated the association between PIRCHE scores and kidney allograft injury using complementary biomarkers such as dd-cfDNA, DSA, histologic rejection, and molecular rejection signatures assessed with the MMDx (19). We hypothesized that a higher PIRCHE score is associated with an increased risk of post-transplant DSA development, a higher incidence of rejection, and elevated levels of dd-cfDNA during the first year following kidney transplantation. Additionally, we sought to assess the performance characteristics of PIRCHE scores for predicting these alloimmune injury outcomes and compared them with those of classic HLA-A/B/DR antigen mismatches.

## Materials and methods

### Data source and study population

This single-center, retrospective, observational study included all adult kidney transplant recipients who underwent transplantation at the University of Utah between January 1, 2021, and December 31, 2024. All patients were followed for up to one year post-transplantation. Donor-related data were collected, including graft type [living donor, deceased donation after brain death (DBD), or donation after circulatory death (DCD)], demographics, comorbidities, terminal serum creatinine, cause of death, and serologic characteristics.

Recipient-level data included demographics, cause of end-stage renal disease, anthropometric measures, dialysis vintage, prior transplant history, multi-organ transplantation status, serologic data, and transplant-related variables such as induction immunosuppression, HLA-related data, laboratory values, and other relevant clinical parameters. Protocol biopsies are not performed at our center; all kidney allograft biopsies were performed for clinical indications. Histopathologic findings and molecular rejection assessments using the MMDx were collected for all biopsies.

Immunosuppressive therapy consisted of induction with either a lymphocyte-depleting agent or basiliximab, followed by standard maintenance immunosuppression. Maintenance therapy included prednisolone in combination with either belatacept, tacrolimus or cyclosporine A (microemulsion formulation, Neoral), together with mycophenolate mofetil, azathioprine, or sirolimus.

Donor-specific antibody and donor-derived cell-free DNA results were also collected. During the study period, DSA testing was performed at 3, 6, 9, and 12 months post-transplant per institutional protocol or at any time in the discretion of the treating provider. Similarly, dd-cfDNA measurements were obtained at 1, 2, 3, 4, 6, 9, and 12 months post-transplant per protocol or as clinically indicated. Data were extracted from electronic medical records and local transplant databases and managed using the Research Electronic Data Capture (REDCap) system hosted by the University of Utah. The study was approved by the University of Utah Institutional Review Board, which granted a waiver of informed consent (#IRB_00162331).

### Exposure variables/PIRCHE scores

Molecular matching was assessed using predicted indirectly recognizable HLA-derived T cell epitope loads (PIRCHE-T2) and the number of surface-accessible HLA amino acid mismatches (PIRCHE-B). PIRCHE-T2 and PIRCHE-B scores were calculated using donor–recipient mismatches at the HLA-A, HLA-B, HLA-C, HLA-DRB1, and HLA-DQB1 loci to derive both locus-specific and aggregate scores. The scores were calculated using the PIRCHE web service (www.pirche.com, v4.5, database Frost 1.1/Snow 1.1/IMGT 3.54, binding rank threshold 30%, surface area threshold 0.26, protrusion threshold 0.68). The overall five-locus PIRCHE-T2 and PIRCHE-B scores were used as the primary exposure variables. The distribution of five-locus PIRCHE-T2 and PIRCHE-B scores is shown (**Supplementary Figures 1A, B**).

As a sensitivity analysis, PIRCHE-T2 score were additionally calculated incorporating the HLA-DQA1 locus, resulting in six-locus aggregate and locus-specific scores. The distribution of six-locus PIRCHE-T2 score is shown (**Supplementary Figure 1C**). High-resolution (allele-level) HLA typing data were used when available. When only low-resolution typing data were available, allele-level imputation was performed using 2007 National Marrow Donor Program (NMDP) haplotype frequencies stratified by reported ethnicity (20). For individuals with mixed or unknown ethnicity, a combined population reference was applied (20). The proportion of any low-resolution typing (for either donor or recipient) was 14% for HLA-A, 14% for HLA-B, 15% for HLA-C, 4% for HLA-DRB1, and 1% for HLA-DQB1. Because imputation is not available for HLA-DQA1, this locus was not imputed; consequently, six-locus PIRCHE-T2 score was evaluated exclusively in sensitivity analyses.

### Outcome variables

Four distinct endpoints were evaluated. The first endpoint was the development of *de novo* or recurrent donor-specific antibodies. Recurrent preexisting DSA was defined as DSA that was present at the time of transplantation (mean fluorescence intensity (MFI) >2,000), or historically detected but below the cutoff at the time of transplantation and subsequently detected within one year post-transplantation with an MFI >2,000. *De novo* DSA was defined as DSA detected within one year after transplantation with an MFI >2,000 that had not been detected either historically or at the time of transplantation. HLA antibody testing was performed using a Luminex-based single-antigen bead assay (LabScreen, One Lambda/Thermo Fisher Scientific).

The second endpoint was the detection of any type of rejection within the first year after transplantation, based on histopathological evaluation. Kidney biopsies were performed only when clinically indicated and were interpreted according to the Banff 2019 classification.

The third endpoint was the diagnosis of any form of molecular rejection within the first year after transplantation, as assessed by MMDx (19).

The fourth endpoint was the elevation of dd-cfDNA at any time during the first year after transplantation. dd-cfDNA elevation was defined as either (a) an absolute dd-cfDNA value ≥1.0%, or (b) an absolute dd-cfDNA value ≥0.5% accompanied by a ≥61% relative increase compared with the immediately preceding measurement (21).

The primary endpoint of the study was the occurrence of any of the above events within 12 months after transplantation. Time-to-event was defined as the interval from the date of transplantation to the date of the first occurrence of any endpoint event. Secondary endpoints consisted of the three outcomes (1: DSA, 2: rejection and 3: elevation of dd-cfDNA, either histopathological or molecular), each evaluated independently.

### Statistical analysis

Patient characteristics were summarized using mean ± standard deviation (SD) or median with interquartile range (IQR) for continuous variables, and counts with percentages for categorical variables. Comparisons between groups were performed using the Student’s *t*-test or the Mann–Whitney *U* test for continuous variables, and the chi-square test for categorical variables, as appropriate.

To assess the predictive performance of PIRCHE scores, receiver operating characteristic (ROC) curve analyses were conducted, and the area under the ROC curve (AUC) was calculated for the primary endpoint and for each secondary endpoint separately. The Youden Index was calculated to evaluate the discriminatory performance of each PIRCHE score for each outcome. Optimal cutoff values were identified based on the maximum Youden Index, and these thresholds were used to classify patients into “low-risk” and “high-risk” groups. As sensitivity analysis we defined “low-risk” and “high-risk” groups based on median value of the scores.

Associations between PIRCHE scores—both as continuous variables and as categorical risk groups—and clinical outcomes were evaluated using Cox proportional hazards regression models and Kaplan–Meier survival analyses with log-rank testing. The proportional hazards assumption was assessed using scaled Schoenfeld residuals. Variables included in multivariable-adjusted models were selected *a priori* based on biological plausibility, published literature, and clinical relevance, and were required to be available in the study database. Adjustments were made for recipient characteristics (age, sex, race/ethnicity, body mass index at transplantation, cause of end-stage renal disease, dialysis vintage, calculated panel reactive antibody at allocation, history of prior kidney transplantation, and preemptive transplant status), donor characteristics (age, sex, race/ethnicity, and donor type [living vs. deceased]), and transplantation-related factors (induction regimen, cold ischemia time, cytomegalovirus risk status, and kidney-alone versus multiorgan transplantation). As a sensitivity analysis, we repeated all analyses excluding multiorgan recipients.

We compared the performance of baseline models (unadjusted and adjusted) using HLA-A/B/DR antigen mismatch as the exposure across all outcomes, and we evaluated an extended model that added PIRCHE scores. Model performance was compared between the base and extended Cox models using likelihood ratio tests, Akaike and Bayesian information criteria (AIC/BIC), and Harrell’s C-statistic to assess incremental improvements in model fit and discrimination.

All statistical tests were two-sided, and a *p* value <0.05 was considered statistically significant. Statistical analyses were performed using STATA version 19 (StataCorp, College Station, TX).

## Results

### Patient characteristics

**Figure 1:**
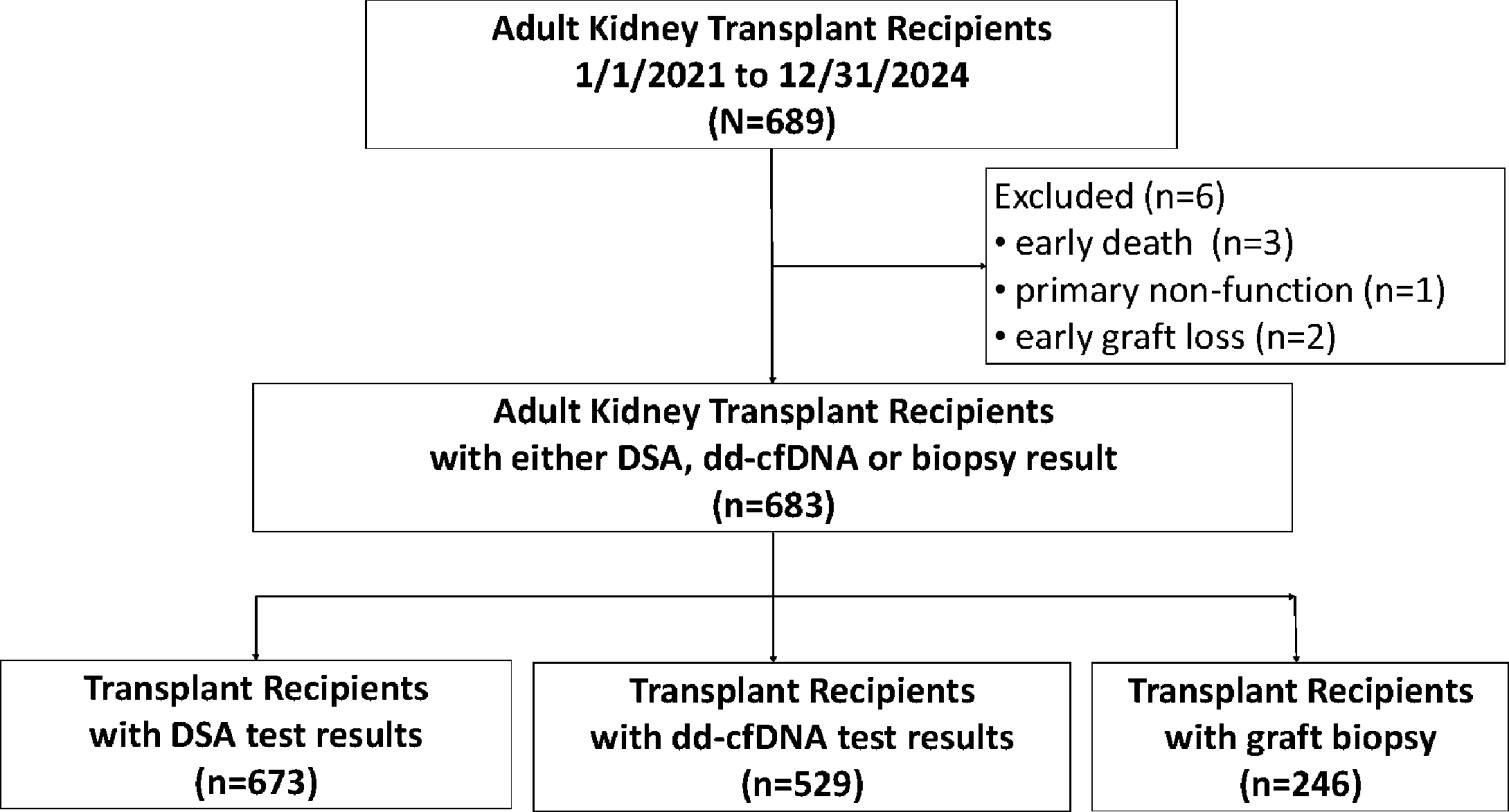
Flowchart of cohort selection.

Between January 1, 2021, and December 31, 2024, 689 patients underwent kidney transplantation at the University of Utah and were initially included in the study cohort (**Figure 1**). Six patients were excluded due to the absence of outcome data, resulting from early graft loss or death shortly after transplantation. Specifically, three patients died on postoperative days (POD) 6, 6, and 39; one patient experienced primary nonfunction of the allograft; and two patients lost their grafts due to surgical complications (graft thrombosis) on POD 1 and POD 2. The final analytic cohort, therefore, consisted of 683 patients.

The mean [± standard deviation (SD)] recipient age was 50 ± 15 years; 61% were male. The cohort was racially and ethnically diverse, with 67% White, 19% Hispanic, 4% Asian and 2% African American recipients. The median (interquartile range (IQR)) dialysis vintage was 34 (16–56) months, and the mean (± SD) body mass index was 28.7 ± 5.7 kg/m². The median (IQR) calculated panel reactive antibody (cPRA) was 0% (0%–0%), and the mean (± SD) was 15 ± 31%. Living donor transplantation accounted for 31% of cases, and among deceased donor transplants, 43% were donation after circulatory death. The mean (± SD) donor age was 37 ± 16 years; 56% of donors were male, and 81% were White. The median (IQR) kidney donor profile index (KDPI) was 28 (12–53), and the median (IQR) cold ischemia time (CIT) was 13.4 (4.9–20.2) hours (**Table 1**).

**Table 1:** Patient characteristics based on graft injury.

Total population/graft injury/no graft injury	Total cohort	No graft injury	Graft injury	p-value
N	683	433	250	
Five-locus PIRCHE-T2 score, median (IQR)	57 (39–76)	55 (37–73)	61 (45–78)	<0.001
PIRCHE-B score, median (IQR)	13 (8-18)	12 (7-17)	15 (11- 20)	<0.001
Six-locus PIRCHE-T2 score, median (IQR)	69 (45-96)	66 (41-91)	77 (56-100)	0.001
Recipients’ characteristics				
Age (years), mean (SD)	49.9 (15.0)	50.4 (14.8)	49.1 (15.2)	0.27
Gender (N (%))				0.12
male	417 (61.1%)	274 (63.3%)	143 (57.2%)	
female	266 (38.9%)	159 (36.7%)	107 (42.8%)	
Race/ethnicity (N (%))				0.37
White	460 (67.4%)	301 (69.5%)	159 (63.6%)	
Hispanic	127 (18.6%)	78 (18.0%)	49 (19.6%)	
African American	15 (2.2%)	8 (1.8%)	7 (2.8%)	
Asian	26 (3.8%)	17 (3.9%)	9 (3.6%)	
Other	54 (7.9%)	28 (6.5%)	26 (10.4%)	
Unknown	1 (0.1%)	1 (0.2%)	0 (0.0%)	
Body mass index (kg/m2), mean (SD)	28.7 (5.7)	28.8 (5.8)	28.5 (5.5)	0.52
Dialysis vintage (months), median (IQR)	33.6 (15.6-56.4)	29.9 (14.1-50.7)	37.9 (18.8-62.5)	0.024
Cause of kidney failure (N (%))				0.60
Diabetes mellitus	212 (31%)	140 (32.3%)	72 (28.8%)	
Hypertension/Vascular disease	87 (13%)	49 (11.3%)	38 (15.2%)	
Cystic kidney disease	55 (8%)	36 (8.3%)	19 (7.6%)	
Glomerulonephritis	159 (23%)	102 (23.6%)	57 (22.8%)	
Other/unknown	170 (25%)	106 (24.5%)	64 (25.6%)	
Prior kidney transplant (N (%))				0.81
No	615 (90.0%)	389 (89.8%)	226 (90.4%)	
Yes	68 (10.0%)	44 (10.2%)	24 (9.6%)	
Multi-organ transplantation (N (%))				0.58
Liver + kidney	15 (2.2%)	8 (1.8%)	7 (2.8%)	
Pancreas + kidney	18 (2.7%)	13 (3.0%)	5 (2.0%)	
Heart + kidney	11 (1.6%)	8 (1.8%)	3 (1.2%)	
None	636 (93.1%)	403 (93.1%)	233 (93.2%)	
Liver +Heart+Kidney	3 (0.4%)	1 (0.2%)	2 (0.8%)	
CMV risk categories (N (%))				0.38
Low	172 (25.2%)	112 (25.9%)	60 (24.0%)	
Intermediate	298 (43.6%)	191 (44.1%)	107 (42.8%)	
High	192 (28.1%)	114 (26.3%)	78 (31.2%)	
Unknown	21 (3.1%)	16 (3.7%)	5 (2.0%)	
Was the donor organ pumped? (N (%))				0.049
No	187 (29.1%)	129 (31.8%)	58 (24.5%)	
Yes	456 (70.9%)	277 (68.2%)	179 (75.5%)	
En block kidney (N (%))	14 (2.0%)	6 (8%)	8 (19%)	0.097
Dual kidney (N (%))	15 (2.2%)	11 (15%)	4 (9%)	0.37
Transplantation characteristics				
Cold ischemic time (hours), median (IQR)	13.4 (4.9-20.2)	12.47 (4.2-20.0)	15.1 (7.2-20.9)	0.013
cPRA (%), median (IQR)	0 (0-0)	0 (0-0)	0 (0-16)	0.054
Donors’ characteristics				
Age (years), mean (SD)	37.4 (15.7)	37.7 (15.3)	36.7 (16.3)	0.41
Gender (N (%))				0.61
male	383 (56.1%)	246 (56.8%)	137 (54.8%)	
female	300 (43.9%)	187 (43.2%)	113 (45.2%)	
Race/ethnicity				0.11
White	555 (81.3%)	352 (81.3%)	203 (81.2%)	
African American	21 (3.1%)	10 (2.3%)	11 (4.4%)	
Asian	13 (1.9%)	7 (1.6%)	6 (2.4%)	
Other	10 (1.5%)	4 (0.9%)	6 (2.4%)	
Unknown	84 (12.3%)	60 (13.9%)	24 (9.6%)	
Donor type (N (%))				0.004
Living	213 (31.2%)	152 (35.1%)	61 (24.4%)	
Deceased	470 (68.8%)	281 (64.9%)	189 (75.6%)	
Donor DCD (N (%))				0.36
No	269 (57.2%)	156 (55.5%)	113 (59.8%)	
Yes	201 (42.8%)	125 (44.5%)	76 (40.2%)	
KDPI, median (IQR)	28 (12-53)	26 (12-53)	28 (13-53)	0.63
Donor cause of death (N (%))				0.91
Anoxia	232 (49.4%)	138 (49.1%)	94 (49.7%)	
Cerebrovascular/stroke	66 (14.0%)	40 (14.2%)	26 (13.8%)	
Head trauma	155 (33.0%)	94 (33.5%)	61 (32.3%)	
Central nervous system tumor	3 (0.6%)	1 (0.4%)	2 (1.1%)	
Other	14 (3.0%)	8 (2.8%)	6 (3.2%)	
Donor terminal creatinine (mg/dl), median (IQR)	0.82 (0.65-1.03)	0.82 (0.67-1.04)	0.81 (0.61-1.0)	0.36
Donors diabetes (N (%))				0.61
No	645 (95.4%)	408 (95.1%)	237 (96.0%)	
Yes	31 (4.6%)	21 (4.9%)	10 (4.0%)	
Donors hypertension (N (%))				0.31
No	572 (85.1%)	368 (86.2%)	204 (83.3%)	
Yes	100 (14.9%)	59 (13.8%)	41 (16.7%)	
Donor malignancy (N (%))				0.84
No	665 (97.4%)	422 (97.5%)	243 (97.2%)	
Yes	18 (2.6%)	11 (2.5%)	7 (2.8%)	
Immunological characteristics				
Number of HLA mismatches (HLA A,B and DR) (N (%))				<0.001
0	26 (3.8%)	24 (5.5%)	2 (0.8%)	
1	17 (2.5%)	12 (2.8%)	5 (2.0%)	
2	40 (5.9%)	32 (7.4%)	8 (3.2%)	
3	122 (17.8%)	87 (20.1%)	35 (14.0%)	
4	163 (23.9%)	100 (23.1%)	63 (25.2%)	
5	212 (31.0%)	123 (28.4%)	89 (35.6%)	
6	103 (15.1%)	55 (12.7%)	48 (19.2%)	
HLA mismatches A (N (%))				0.20
0	24 (15%)	19 (17.6%)	5 (8.8%)	
1	75 (45%)	50 (46.3%)	25 (43.9%)	
2	66 (40%)	39 (36.1%)	27 (47.4%)	
HLA mismatches B (N (%))				0.66
0	13 (8%)	10 (9.3%)	3 (5.3%)	
1	46 (28%)	30 (27.8%)	16 (28.1%)	
2	106 (64%)	68 (63.0%)	38 (66.7%)	
HLA mismatches DR (N (%))				0.017
0	49 (14.4%)	39 (17.2%)	10 (8.8%)	
1	169 (49.8%)	117 (51.5%)	52 (46.0%)	
2	122 (35.9%)	71 (31.3%)	51 (45.1%)	
HLA mismatches DQ (N (%))				0.52
0	34 (21%)	25 (23.1%)	9 (16.1%)	
1	80 (49%)	50 (46.3%)	30 (53.6%)	
2	50 (30%)	33 (30.6%)	17 (30.4%)	
Induction treatment (N (%))				
No Induction	0 (0%)	0 (0%)	0 (0%)	
Basiliximab	82 (12.0%)	57 (13.2%)	25 (10.0%)	0.22
Thymoglobulin	568 (83.2%)	350 (80.8%)	218 (87.2%)	0.032
Steroid	682 (99.9%)	432 (99.8%)	250 (100.0%)	0.45
Alemtuzumab	21 (3.1%)	14 (3.2%)	7 (2.8%)	0.75
Outcomes				
Proportion of Antibody Mediated rejection of the subset of patients underwent biopsy (N (%))				<0.001
No	185 (75.2%)	97 (100.0%)	88 (59.1%)	
Yes	61 (24.8%)	0 (0.0%)	61 (40.9%)	
Proportion of rejection of the subset of patients underwent biopsy (N (%))				<0.001
No	147 (59.8%)	97 (100.0%)	50 (33.6%)	
Yes	99 (40.2%)	0 (0.0%)	99 (66.4%)	
Proportion of T-cell mediated Rejection of the subset of patients underwent biopsy (N (%))				<0.001
No	164 (66.7%)	97 (100.0%)	67 (45.0%)	
Yes	82 (33.3%)	0 (0.0%)	82 (55.0%)	
Post-transplant donor specific antibodies (N (%))				<0.001
No	515 (76.6%)	422 (100.0%)	93 (37.2%)	
Yes	157 (23.4%)	0 (0.0%)	157 (62.8%)	
Dd-cfDNA elevation (N (%))				<0.001
No	419 (79.2%)	336 (100.0%)	83 (43.0%)	
Yes	110 (20.8%)	0 (0.0%)	110 (57.0%)	
Delayed graft function (N (%))				0.017
No	624 (91.4%)	404 (93.3%)	220 (88.0%)	
Yes	59 (8.6%)	29 (6.7%)	30 (12.0%)	
Death (N (%))				0.80
No	652 (95.5%)	414 (95.6%)	238 (95.2%)	
Yes	31 (4.5%)	19 (4.4%)	12 (4.8%)	
Graft Loss (N (%))				0.007
No	667 (97.7%)	428 (98.8%)	239 (95.6%)	
Yes	16 (2.3%)	5 (1.2%)	11 (4.4%)	

Values are expressed as mean (standard deviation), median (interquartile range), or number (%). Continuous variables were compared via t-tests or Mann-Whitney U tests. Categorical variables were compared via Chi-square tests.

Baseline recipient, donor, and transplant characteristics, stratified by primary outcome, are presented in **Table 1**, and stratification by PIRCHE score risk category is shown in **Supplementary Tables 1**, **2**. Compared with patients without allograft injury, those with allograft injury had longer dialysis vintage and were more likely to receive organs with longer cold ischemic time, organs preserved by machine perfusion, organs from a deceased donor, and thymoglobulin induction (**Table 1**).

### Primary outcome

Among the 683 patients included in the analysis, 250 (37%) experienced the primary outcome within the first year following transplantation. The 250 primary outcome events comprised 125 (50%) DSA events, 87 (35%) elevated dd-cfDNA events, and 38 (15%) rejection events; the distribution of the cumulative outcomes in the first year is shown in **Table 2**. The median (IQR) five-locus PIRCHE-T2 (61 (45–78) vs. 55 (37–73), *p* < 0.001) and PIRCHE-B scores (15 (11–20) vs. 12 (7–17), *p* < 0.001) were significantly higher among patients who experienced the primary outcome compared with those who did not (**Table 1**).

**Table 2:** Patients experiencing cumulative outcomes in the first year after kidney transplantation.

N=683	DSA only	Rejection only	Elevated dd-cfDNA only	DSA & rejection	DSA & elevated dd-cfDNA	Rejection & elevated dd-cfDNA	DSA & rejection & elevated dd-cfDNA	No injury
Number of patients (%)	89 (13%)	27 (4%)	48 (7%)	24 (4%)	14 (2%)	18 (3%)	30 (4%)	433 (63%)

Receiver operating characteristic analyses demonstrated that both five-locus PIRCHE-T2 and PIRCHE-B scores exhibited weak discriminatory ability for the primary outcome, with areas under the curve (AUCs) of 0.575 and 0.621, respectively **(****Supplementary Figures 2A, B**). Based on the Youden Index, optimal cutoff values of <53 vs ≥53 for five-locus PIRCHE-T2 and <11 vs ≥11for PIRCHE-B were identified and used to define low- and high-risk groups. At these thresholds, five-locus PIRCHE-T2 demonstrated a sensitivity of 66% and specificity of 48%, while PIRCHE-B demonstrated a sensitivity of 76% and specificity of 44%. Detailed diagnostic performance metrics are provided in **Supplementary Tables 3**, **4**.

**Figure 2:**
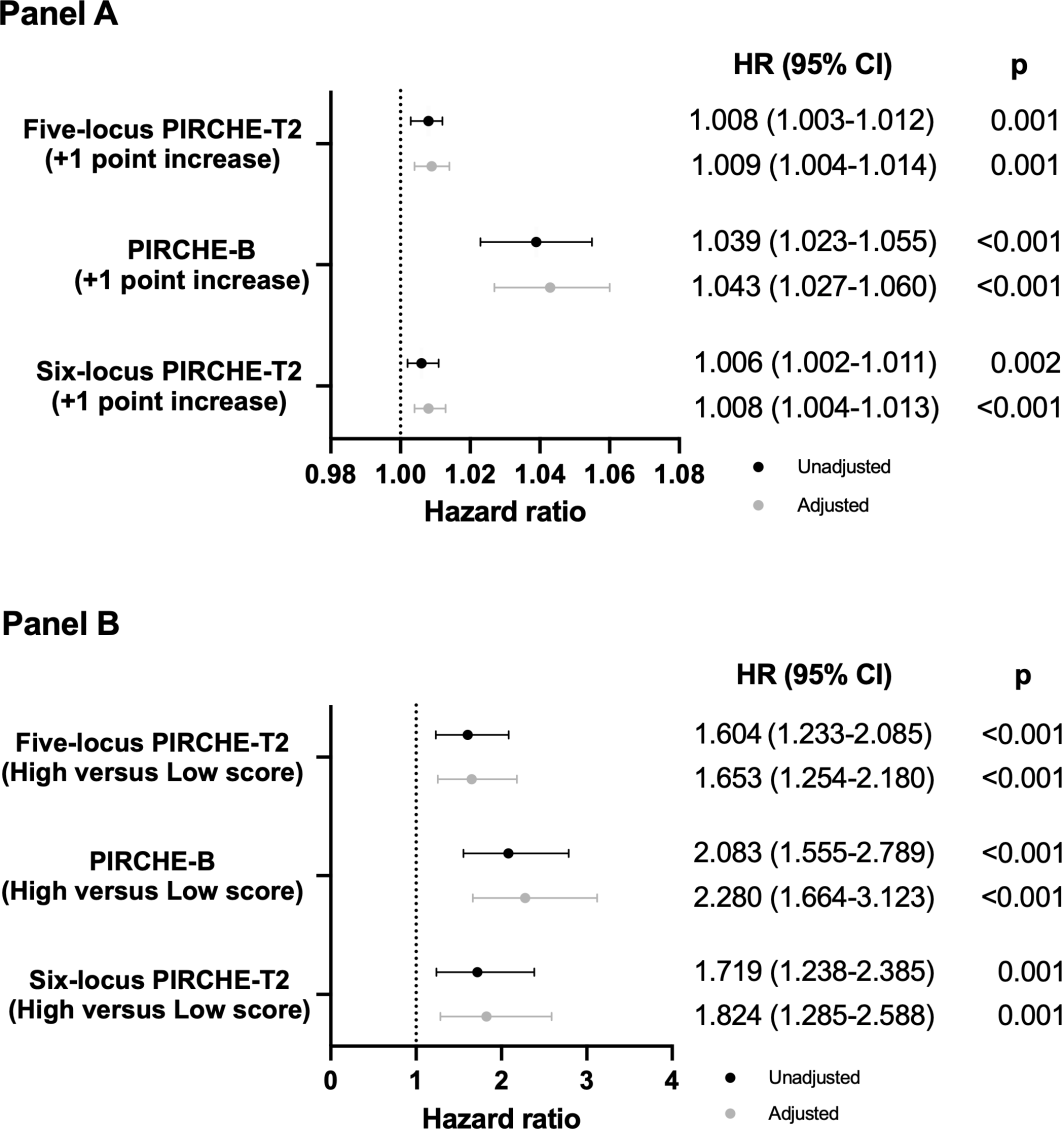
Association of PIRCHE scores and primary outcome using Cox proportional regression models. Panel **(A)** shows each one-point increase in PIRCHE scores, while Panel **(B)** shows risk groups by PIRCHE scores.

**Figure 3:**
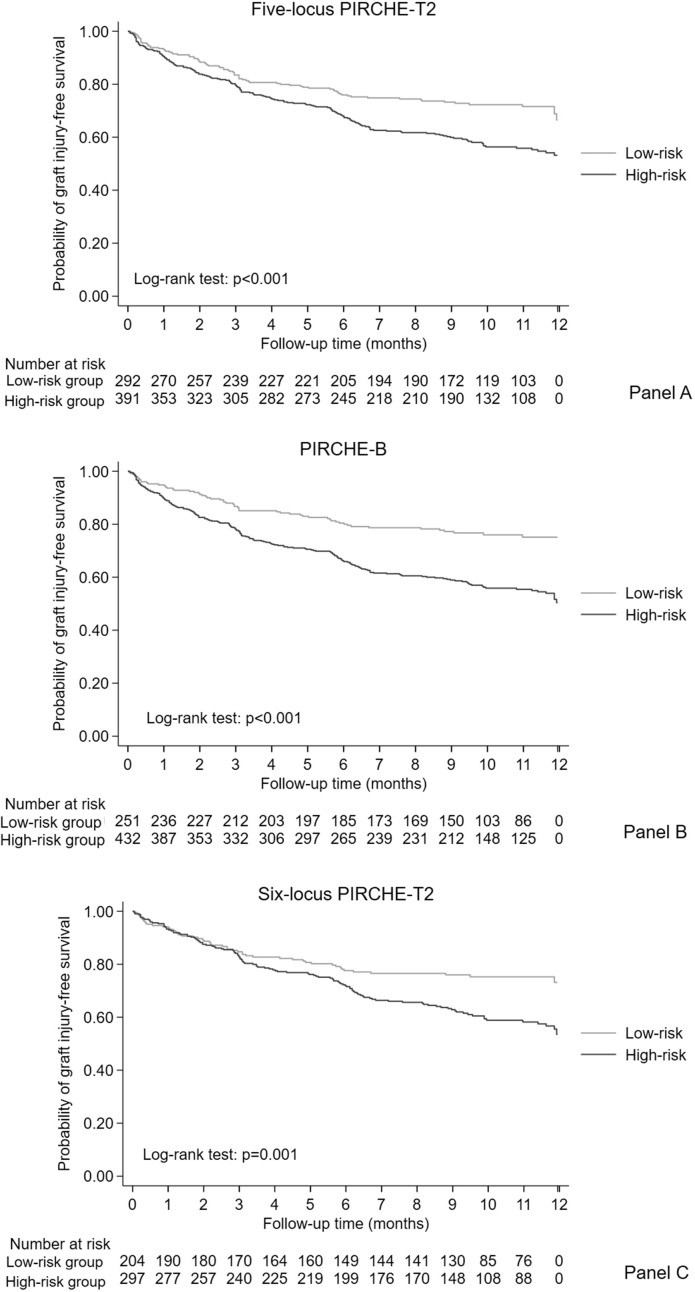
Kaplan-Meier curves by PIRCHE risk groups for primary outcome (Panel A: Five-locus PIRCHE-T2; Panel B: PIRCHE-B; Panel C: Six-locus PIRCHE-T2).

Higher five-locus PIRCHE-T2 scores were significantly associated with an increased risk of the primary outcome, as shown in **Figure 2** and the Kaplan–Meier survival analysis (**Figure 3A**). In unadjusted Cox proportional hazards models, both each one-point increase in five-locus PIRCHE-T2 score and classification into the high-risk group were associated with a higher risk of the primary outcome ([HR_per one-point increase_ = 1.008; 95% CI: 1.003-1.012] and [HR_high vs. low_ = 1.604; 95% CI: 1.233-2.085]). These associations remained statistically significant after adjustment for recipient, donor, and transplant-related covariates ([HR_per one-point increase_ = 1.009; 95% CI: 1.004-1.014] and [HR_high vs. low_ = 1.653; 95% CI: 1.254-2.180]).

Similarly, higher PIRCHE-B scores were associated with an increased risk of the primary outcome (**Figures 2**, **3B**). In unadjusted analyses, both each one-point increase in PIRCHE-B score and membership in the high-risk group were significantly associated with the primary outcome ([HR_per one-point increase_ = 1.039; 95% CI: 1.023-1.055] and [HR_high vs. low_ = 2.083; 95% CI: 1.555-2.789]). These associations remained robust after multivariable adjustment ([HR_per one-point increase_ = 1.043; 95% CI: 1.027-1.060] and [HR_high vs. low_ = 2.280; 95% CI: 1.664-3.123]).

### Secondary outcomes

#### Donor specific antibodies

Among the 673 patients with DSA data included in the analysis, 157 (23%) developed post-transplant *de novo* or recurrent DSA within the first year following transplantation. Baseline recipient, donor, and transplant characteristics stratified by five-locus PIRCHE-T2 risk category are presented in **Supplementary Table 5**, and stratification by PIRCHE-B risk category is shown in **Supplementary Table 6**. The median (IQR) five-locus PIRCHE-T2 (60 (45–80) vs. 56 (38–74), *p* = 0.006) and PIRCHE-B (16 (11–21) vs. 12 (8–18), *p* < 0.001) scores were significantly higher in patients who developed post-transplant *de novo* or recurrent DSA compared with those who did not (**Supplementary Figures 3A, B**). Receiver operating characteristic analyses are shown in **Supplementary Figures 4A, B**. Using the Youden Index, optimal cutoff values of <73 vs ≥73 for five-locus PIRCHE-T2 and <13 vs ≥13 for PIRCHE-B were identified and used to stratify patients into low- and high-risk groups. Detailed diagnostic performance characteristics are presented in **Supplementary Tables 7**, **8**. Higher five-locus PIRCHE-T2 and PIRCHE-B scores were significantly associated with an increased risk of post-transplant *de novo* or recurrent DSA, as demonstrated by Cox regression analyses (**Supplementary Figure 5**) and Kaplan–Meier survival curves (**Supplementary Figures 6A, B**).

#### Rejection based on histopathological evaluation and/or molecular microscope diagnostic system

Among the 683 patients included in the analysis, of whom 246 underwent kidney biopsy, 99 patients (14%) experienced rejection within the first year after transplantation, including 61 (9%) cases of antibody-mediated rejection (AMR) and 82 (12%) cases of T cell–mediated rejection (TCMR). Baseline recipient, donor, and transplant characteristics stratified by five-locus PIRCHE-T2 risk category are presented in **Supplementary Table 9**, and stratification by PIRCHE-B risk category is shown in **Supplementary Table 10**. The median (IQR) five-locus PIRCHE-T2 score was higher among patients who developed rejection compared with those who did not (66 (50–77) vs. 57 (39–75), *p* = 0.022), while the median (IQR) PIRCHE-B score also trended higher (14 (10–20) vs. 12 (7–18), *p* = 0.071; **Supplementary Figures 7A, B**). Receiver operating characteristic analyses are presented in **Supplementary Figures 8A, B**. Based on the Youden Index, optimal cutoff values of <66 versus ≥66 for five-locus PIRCHE-T2 and <12 versus ≥12 for PIRCHE-B were identified and used to stratify patients into low- and high-risk groups. Detailed diagnostic performance metrics are summarized in **Supplementary Tables 11****,****12**. Higher five-locus PIRCHE-T2 and PIRCHE-B scores showed a trend toward an increased risk of rejection, as demonstrated by Cox proportional hazards analyses (**Supplementary Figure 9**) and Kaplan–Meier survival curves (**Supplementary Figures 10A, B**).

#### Elevation of donor-derived cell-free DNA

Among the 529 patients with dd-cfDNA data included in the analysis, 110 (21%) developed elevated dd-cfDNA within the first year following transplantation. Baseline recipient, donor, and transplant characteristics stratified by five-locus PIRCHE-T2 risk category are presented in **Supplementary Table 13**, and stratification by PIRCHE-B risk category is shown in **Supplementary Table 14**. The median (IQR) five-locus PIRCHE-T2 (65 (51–80) vs. 56 (37–76), *p* = 0.003) and PIRCHE-B (15 (11–20) vs. 13 (8–18), *p* = 0.013) scores were significantly higher among patients who developed elevated dd-cfDNA compared with those who did not (**Supplementary Figures 11A, B**). Receiver operating characteristic analyses are presented in **Supplementary Figures 12A, B**. Using the Youden Index, optimal cutoff values of <49.32 vs ≥49.32 for five-locus PIRCHE-T2 and <10 vs ≥10 for PIRCHE-B were identified and used to stratify patients into low- and high-risk groups. Detailed diagnostic performance metrics are summarized in **Supplementary Tables 15**, **16**. Higher five-locus PIRCHE-T2 and PIRCHE-B scores were significantly associated with an increased risk of dd-cfDNA elevation, as demonstrated by Cox proportional hazards analyses (**Supplementary Figure 13**) and Kaplan–Meier survival curves (**Supplementary Figures 14A, B**).

### Sensitivity analysis

The median (IQR) six-locus PIRCHE-T2 scores were significantly higher among patients who experienced the primary outcome compared with those who did not (77 (56–100) vs. 66 (41–91), *p* = 0.002); **Table 1**). Receiver operating characteristic analyses are presented in **Supplementary Figure 2C**. Using the Youden Index, an optimal cutoff value of <63 vs ≥63 for six-locus PIRCHE-T2 was identified and used to stratify patients into low- and high-risk groups. Detailed diagnostic performance metrics are summarized in **Supplementary Tables 17****-****20**. Higher six-locus PIRCHE-T2 scores were significantly associated with an increased risk of the primary outcome, as demonstrated by Cox proportional hazards analyses (**Figure 2**) and Kaplan–Meier survival curves (**Figure 3C**). Similar associations were observed when *de novo* or recurrent DSA, rejection, or dd-cfDNA elevation were analyzed as individual outcomes (**Supplementary Figures 3****-****12C**). Similar associations were found for the primary outcome and all secondary outcomes, except rejection, in our sensitivity analysis using a median cut-off score to define “low-risk” and “high-risk” groups (not shown), and in all analyses after excluding multiorgan recipients (not shown).

### Model performance and incremental predictive value of PIRCHE scores

#### Primary outcome

**Figure 4:**
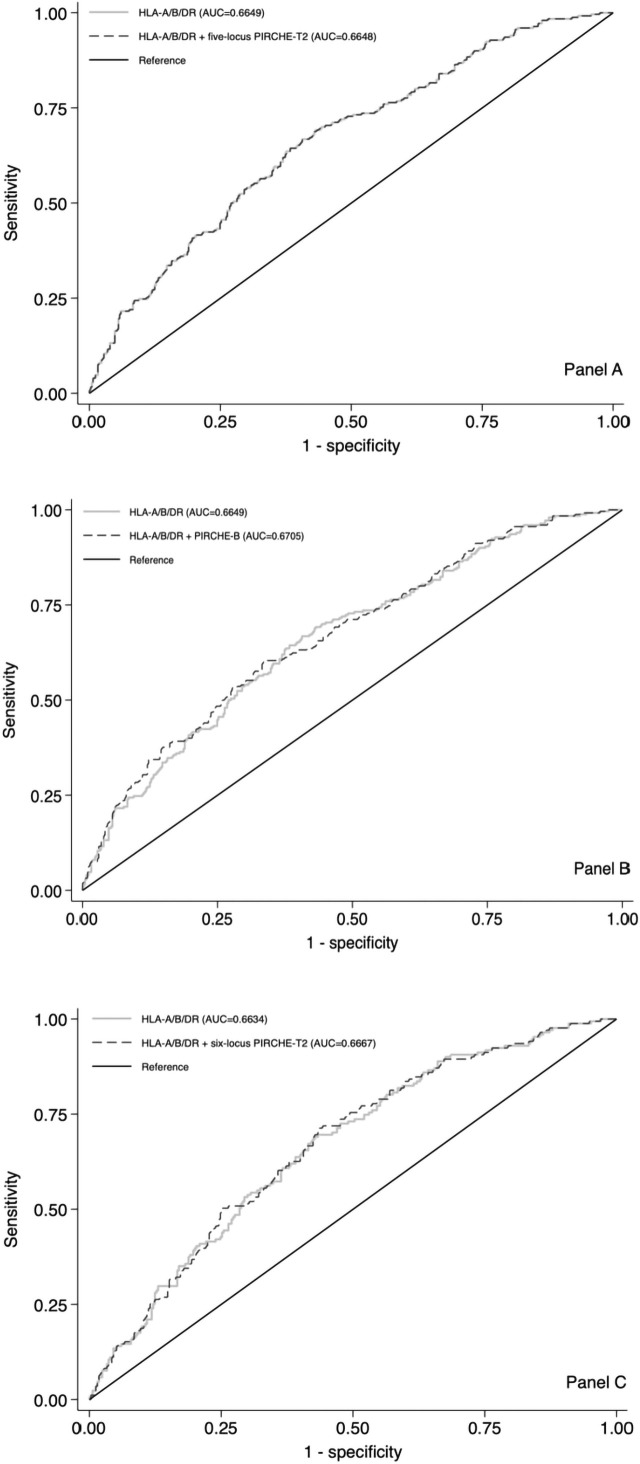
Performances of models with PIRCHE scores and without PIRCHE scores to detect primary outcome [**(A)** Five-locus PIRCHE-T2; **(B)** PIRCHE-B; **(C)** Six-locus PIRCHE-T2].

Compared with the unadjusted model using HLA-A/B/DR antigen mismatch, the extended model, after adding PIRCHE-B, showed a statistically significant improvement over the base model (LR test p=0.019), with a modest reduction in AIC and a small increase in discrimination (C-index 0.575 to 0.589). However, BIC did not improve, suggesting limited incremental predictive value. In the adjusted model, adding PIRCHE-B significantly improved model fit (LR test, p=0.018), with a modest reduction in AIC, but did not improve BIC and resulted in only a minimal increase in discrimination (C-index 0.649 to 0.653), suggesting limited incremental predictive value (**Figure 4B**). Both five and six-locus PIRCHE-T2 provide no incremental predictive value for the primary outcome, either in the univariate or multivariable model (**Figures 4A, C****)**.

#### DSA outcome

Adding PIRCHE-B significantly improved model fit (LR test, p<0.001), reduced AIC and BIC, and substantially improved discrimination (C-index from 0.555 to 0.596), indicating meaningful incremental predictive value in the unadjusted model. In the fully adjusted Cox model for DSA, addition of PIRCHE-B significantly improved model fit and discrimination (LR test p=0.0005; AIC 1886.5 to 1876.3; C-index 0.687 to 0.701), indicating meaningful incremental predictive value (**Supplementary Figure 15B**). Both five and six-locus PIRCHE-T2 provide no incremental predictive value for the DSA outcome, either in the univariate or multivariable model (**Supplementary Figures 15A, C****)**.

#### Rejection outcome

Five-locus PIRCHE-T2 and PIRCHE-B do not add predictive value for rejection in the unadjusted and fully adjusted models (**Supplementary Figures 16A, B**). In the adjusted Cox model for all rejection, addition of six-locus PIRCHE-T2 significantly improved model fit (LR p=0.0087) and discrimination (C-index 0.729 to 0.734), and six-locus PIRCHE-T2 remained independently associated with the outcome (HR 1.015, p=0.007) (**Supplementary Figure 16C****)**.

#### Elevation of donor-derived cell-free DNA outcome

Neither PIRCHE-T2, nor PIRCHE-B adds predictive value for elevated dd-cfDNA in the unadjusted and fully adjusted model (**Supplementary Figures 17A-C****)**.

## Discussion

In this single-center retrospective study of nearly 700 kidney transplant recipients, we demonstrate that higher PIRCHE-T2 and PIRCHE-B scores are independently associated with early allograft injury during the first year following transplantation. By integrating multiple complementary and clinically validated biomarkers—including donor-specific antibodies, donor-derived cell-free DNA, histologic rejection, and molecular rejection assessed by the Molecular Microscope^®^ Diagnostic System—we provide a comprehensive assessment of early alloimmune injury. Patients with higher PIRCHE scores experienced a 50% to twofold increased risk of the composite primary outcome, supporting the clinical relevance of molecular mismatch assessment beyond conventional HLA antigen matching.

A key strength and clinically relevant contribution of this study is the identification of pragmatic cutoff values for five- and six-locus PIRCHE-T2 and PIRCHE-B scores. These thresholds (five-locus PIRCHE−T2: 49-73, six-locus PIRCHE−T2: 57-78, and PIRCHE−B scores: 10-13) provide actionable guidance for transplant clinicians when evaluating immunologic risk at the time of donor selection. In particular, PIRCHE-based risk stratification could be incorporated into living donor evaluation, deceased donor offer acceptance, and allocation decisions, where rapid assessment of immunologic compatibility is critical. Patients identified as high risk based on PIRCHE scores may benefit from tailored immunosuppressive strategies, such as lymphocyte-depleting induction, closer post-transplant surveillance, or more frequent monitoring with biomarkers such as DSA and dd-cfDNA. However, these cutoff values should be interpreted cautiously. As our results have not been validated in an external cohort, these cutoffs should not be used without further evaluation.

Although the discriminatory performance of PIRCHE scores was modest, this finding should be interpreted in the context of their intended clinical role. PIRCHE scores are not designed to function as standalone diagnostic tests but rather as baseline immunologic risk indicators. Similar to traditional factors such as calculated panel-reactive antibody (cPRA) or donor type, PIRCHE scores provide incremental information that can refine risk stratification when combined with other clinical and immunologic variables. The consistent association of higher PIRCHE scores with multiple distinct manifestations of allograft injury underscores their robustness and supports their use in a multimodal risk assessment framework.

It is important to note that, compared with HLA-A/B/DR antigen-mismatch models, PIRCHE-B scores improved prediction primarily for DSA, whereas PIRCHE-T2 did not add predictive value for outcomes. Further studies are needed to assess the long-term outcomes and the additional value of PIRCHE scores compared to traditional HLA mismatch.

Our findings confirm and extend prior studies demonstrating an independent association between higher class II PIRCHE scores and post-transplant DSA development (4–9). Previous work has consistently shown that DRB1- and DQB1-derived PIRCHE-II scores predict *de novo* DSA independent of conventional HLA mismatch and B-cell epitope metrics (4–7, 9–11). In addition, higher class II PIRCHE-II scores—particularly those attributable to DRB1 and DQB1—have been independently associated with an increased incidence of biopsy-proven T cell–mediated rejection, including borderline lesions and vascular TCMR (7, 14, 15). In large multicenter modeling, DRB1- and DQB1-PIRCHE-II also emerge as independent immunologic determinants of antibody-mediated rejection alongside class II EPLET load and DSA strength, indicating a specific contribution of indirect CD4^+^ T-cell epitope load to AMR risk (16). In patients with initial borderline or no rejection, and in cohorts with established AMR, higher total and class II PIRCHE-II scores predict progression to overt T-cell mediated rejection (TCMR)/AMR and worsening microvascular inflammation, supporting their use for risk stratification after an index biopsy (17, 18). By leveraging predominantly high-resolution HLA typing and a large contemporary cohort, our study reinforces the clinical relevance of PIRCHE-based matching and demonstrates that its impact extends beyond humoral alloimmunity to encompass broader patterns of graft injury.

Importantly, this study is the first to directly demonstrate an association between PIRCHE scores and dd-cfDNA elevation. dd-cfDNA has become an increasingly important noninvasive biomarker in routine post-transplant care, providing a sensitive indicator of active graft injury that often precedes changes in serum creatinine (22). We observed that patients with higher PIRCHE scores at the time of transplantation had a two- to threefold increased risk of subsequent dd-cfDNA elevation. This finding has direct clinical implications, as it supports a strategy in which pre-transplant molecular mismatch assessment informs post-transplant surveillance intensity. High-risk patients identified by PIRCHE scores may benefit from closer dd-cfDNA monitoring to enable earlier detection of injury and timely intervention.

The comprehensive outcome definition used in this study represents another clinically meaningful advance. Rather than relying on a single biomarker or endpoint, we combined DSA, dd-cfDNA, histologic rejection, and molecular rejection to capture the full spectrum of early allograft injury. This approach reflects real-world clinical practice, where graft injury is often multifactorial and may be missed by traditional markers alone. Our findings suggest that PIRCHE scores are associated with a global propensity for alloimmune injury, rather than a single downstream manifestation.

Several limitations merit consideration. The single-center, retrospective design limits causal inference and generalizability, and external validation in independent, more diverse cohorts is needed. Although the majority of HLA typing was performed at high resolution, imputation was required for a minority of cases (<15%), potentially introducing some uncertainty. Additionally, although we adjusted for a broad range of clinical confounders, residual confounding cannot be ruled out. Finally, our center does not perform protocol biopsies, which may introduce significant selection bias.

Despite these limitations, the strengths of this study—including comprehensive biomarker assessment, robust statistical modeling, and practical cutoff identification—support the translational relevance of our findings. PIRCHE-based molecular matching offers a clinically actionable framework that can be readily integrated into existing transplant workflows without additional patient burden.

In conclusion, higher PIRCHE-T2 and PIRCHE-B scores are associated with an increased risk of early alloimmune injury across multiple complementary biomarkers. While their standalone discriminatory performance is modest, PIRCHE scores provide valuable baseline immunologic risk stratification and may guide individualized donor selection, immunosuppressive strategies, and post-transplant surveillance. Prospective studies are warranted to evaluate whether PIRCHE-guided clinical decision-making can improve long-term transplant outcomes.

## Data Availability

The raw data supporting the conclusions of this article will be made available by the authors, without undue reservation.
