# Supplementary material for: Association of PIRCHE scores and allograft injury in kidney transplant recipients
Source: Front Immunol. 2026 Jun 8;17:1858108. doi: 10.3389/fimmu.2026.1858108 (PMC13283821; doi:10.3389/fimmu.2026.1858108)
Supplement: Supplementary file 1 [file DataSheet1.docx]

**Association of PIRCHE Scores and Allograft Injury in Kidney Transplant Recipients**

**Supplementary Materials**

**Supplementary Tables**

**Table S1:** Patient characteristics based on PIRCHE-T2 score

**Table S2:** Patient characteristics based on PIRCHE-B score

**Table S3:** Performance characteristics table of PIRCHE-T2 for primary outcome

**Table S4:** Performance characteristics table of PIRCHE-B for primary outcome

**Table S5:** Patient characteristics of the DSA cohort based on PIRCHE-T2 score

**Table S6:** Patient characteristics of the DSA cohort based on PIRCHE-B score

**Table S7:** Performance characteristics table of PIRCHE-T2 for DSA

**Table S8:** Performance characteristics table of PIRCHE-B for DSA

**Table S9:** Patient characteristics of the rejection cohort based on PIRCHE-T2 score

**Table S10:** Patient characteristics of the rejection cohort based on PIRCHE-B score

**Table S11:** Performance characteristics table of PIRCHE-T2 for rejection

**Table S12:** Performance characteristics table of PIRCHE-B for rejection

**Table S13:** Patient characteristics of the donor-derived cell-free DNA cohort based on PIRCHE-T2 score

**Table S14:** Patient characteristics of the donor-derived cell-free DNA cohort based on PIRCHE-B score

**Table S15:** Performance characteristics table of PIRCHE-T2 for donor-derived cell-free DNA

**Table S16:** Performance characteristics table of PIRCHE-B for donor-derived cell-free DNA

**Table S17:** Performance characteristics table of six-locus PIRCHE-T2 for primary outcome

**Table S18:** Performance characteristics table of six-locus PIRCHE-T2 for DSA

**Table S19:** Performance characteristics table of six-locus PIRCHE-T2 for rejection

**Table S20:** Performance characteristics table of six-locus PIRCHE-T2 for donor-derived cell-free DNA

**Legend of Supplementary Figures**

**Figure S1:** Histogram of PIRCHE scores (Panel A: Five-locus PIRCHE-T2; Panel B: PIRCHE-B; Panel C: Six-locus PIRCHE-T2)

**Figure S2:** Receiver Operating Characteristic curves of PIRCHE scores for primary outcome (Panel A: Five-locus PIRCHE-T2; Panel B: PIRCHE-B; Panel C: Six-locus PIRCHE-T2)

**Figure S3:** Distribution of PIRCHE scores by DSA outcome (Panel A: Five-locus PIRCHE-T2; Panel B: PIRCHE-B; Panel C: Six-locus PIRCHE-T2)

**Figure S4:** Receiver Operating Characteristic curves of PIRCHE scores for DSA (Panel A: Five-locus PIRCHE-T2; Panel B: PIRCHE-B; Panel C: Six-locus PIRCHE-T2)

**Figure S5:** Association of PIRCHE scores and DSA using Cox proportional regression models

**Figure S6:** Kaplan-Meier curves by PIRCHE risk groups for DSA development (Panel A: Five-locus PIRCHE-T2; Panel B: PIRCHE-B; Panel C: Six-locus PIRCHE-T2)

**Figure S7:** Distribution of PIRCHE scores by rejection outcome (Panel A: Five-locus PIRCHE-T2; Panel B: PIRCHE-B; Panel C: Six-locus PIRCHE-T2)

**Figure S8:** Receiver Operating Characteristic curves of PIRCHE scores for rejection (Panel A: Five-locus PIRCHE-T2; Panel B: PIRCHE-B; Panel C: Six-locus PIRCHE-T2)

**Figure S9:** Association of PIRCHE scores and rejection using Cox proportional regression models

**Figure S10:** Kaplan-Meier curves by PIRCHE risk groups for rejection (Panel A: Five-locus PIRCHE-T2; Panel B: PIRCHE-B; Panel C: Six-locus PIRCHE-T2)

**Figure S11:** Distribution of PIRCHE scores by donor-derived cell-free DNA outcome (Panel A: Five-locus PIRCHE-T2; Panel B: PIRCHE-B; Panel C: Six-locus PIRCHE-T2)

**Figure S12:** Receiver Operating Characteristic curves of PIRCHE scores for donor-derived cell-free DNA (Panel A: Five-locus PIRCHE-T2; Panel B: PIRCHE-B; Panel C: Six-locus PIRCHE-T2)

**Figure S13:** Association of PIRCHE scores and donor-derived cell-free DNA using Cox proportional regression models

**Figure S14:** Kaplan-Meier curves by PIRCHE risk groups for donor-derived cell-free DNA (Panel A: Five-locus PIRCHE-T2; Panel B: PIRCHE-B; Panel C: Six-locus PIRCHE-T2)

**Figure S15:** Performances of models with PIRCHE scores and without PIRCHE scores to

detect DSA outcome (Panel A: Five-locus PIRCHE-T2; Panel B: PIRCHE-B; Panel C: Six-locus PIRCHE-T2)

**Figure S16:** Performances of models with PIRCHE scores and without PIRCHE scores to

detect rejection outcome (Panel A: Five-locus PIRCHE-T2; Panel B: PIRCHE-B; Panel C: Six-locus PIRCHE-T2)

**Figure S17:** Performances of models with PIRCHE scores and without PIRCHE scores to

detect donor-derived cell-free DNA outcome (Panel A: Five-locus PIRCHE-T2; Panel B: PIRCHE-B; Panel C: Six-locus PIRCHE-T2)

**Table S1.** Patient characteristics based on PIRCHE-T2 score

|  | **Low Risk PIRCHE-T2 Score Group** | **High Risk PIRCHE-T2 Score Group** | **p-value** |
| --- | --- | --- | --- |
| **N** | 292 (43%) | 391 (57%) |  |
| **Recipients’ characteristics** |  |  |  |
| **Age (years), mean (SD)** | 50.5 (15.8) | 49.5 (14.3) | 0.39 |
| **Gender (N (%))** |  |  | 0.14 |
| male | 169 (57.9%) | 248 (63.4%) |  |
| female | 123 (42.1%) | 143 (36.6%) |  |
| **Race/ethnicity (N (%))** |  |  | <0.001 |
| White | 230 (78.8%) | 230 (58.8%) |  |
| Hispanic | 40 (13.7%) | 87 (22.3%) |  |
| African American | 1 (0.3%) | 14 (3.6%) |  |
| Asian | 6 (2.1%) | 20 (5.1%) |  |
| Other | 15 (5.1%) | 39 (10.0%) |  |
| Unknown | 0 (0.0%) | 1 (0.3%) |  |
| **Body Mass Index (kg/m2), mean (SD)** | 28.4 (5.5) | 28.0 (5.8) | 0.22 |
| **Dialysis vintage (months), median (IQR)** | 29.1 (14.4-47.5) | 35.0 (16.7-59.8) | 0.032 |
| **Cause of kidney failure (N (%))** |  |  | 0.035 |
| Diabetes mellitus | 73 (25%) | 139 (36%) |  |
| Hypertension/Vascular disease | 41 (14%) | 46 (12%) |  |
| Cystic kidney disease | 22 (7%) | 33 (8%) |  |
| Glomerulonephritis | 72 (25%) | 87 (22%) |  |
| Other/unknown | 84 (29%) | 86 (22%) |  |
| **Prior kidney transplant (N (%))** |  |  | 0.002 |
| No | 251 (86.0%) | 364 (93.1%) |  |
| Yes | 41 (14.0%) | 27 (6.9%) |  |
| **Multi-organ transplantation (N (%))** |  |  | 0.37 |
| Liver + kidney | 6 (2.1%) | 9 (2.3%) |  |
| Pancreas + kidney | 5 (1.7%) | 13 (3.3%) |  |
| Heart + kidney | 4 (1.4%) | 7 (1.8%) |  |
| None | 277 (94.9%) | 359 (91.8%) |  |
| Liver +Heart+Kidney | 0 (0.0%) | 3 (0.8%) |  |
| **CMV risk categories (N (%))** |  |  | 0.19 |
| Low | 69 (23.6%) | 103 (26.3%) |  |
| Intermediate | 120 (41.1%) | 178 (45.5%) |  |
| High | 91 (31.2%) | 101 (25.8%) |  |
| Unknow | 12 (4.1%) | 9 (2.3%) |  |
| **Was the donor organ pumped? (N (%))** |  |  | <0.001 |
| No | 99 (36.5%) | 88 (23.7%) |  |
| Yes | 172 (63.5%) | 284 (76.3%) |  |
| **En block kidney (N (%))** | 4 (1.4%) | 10 (2.6%) | 0.27 |
| **Dual kidney (N (%))** | 6 (2.1%) | 9 (2.3%) | 0.85 |
| **Transplantation characteristics** |  |  |  |
| **Cold ischemic time (hours), median (IQR)** | 11.9 (3.3-20.7) | 14.0 (6.8- 20.0) | 0.029 |
| **cPRA (%), median (IQR)** | 0 (0-13.5) | 0 (0-0) | 0.008 |
| **Donors’ characteristics** |  |  |  |
| **Age (years), mean (SD)** | 37.9 (15.5) | 37.0 (15.9) | 0.46 |
| **Gender (N (%))** |  |  | 0.006 |
| male | 146 (50.0%) | 237 (60.6%) |  |
| female | 146 (50.0%) | 154 (39.4%) |  |
| **Race/ethnicity** |  |  | 0.064 |
| White | 248 (84.9%) | 307 (78.5%) |  |
| African American | 5 (1.7%) | 16 (4.1%) |  |
| Asian | 3 (1.0%) | 10 (2.6%) |  |
| Other | 6 (2.1%) | 4 (1.0%) |  |
| Unknown | 30 (10.3%) | 54 (13.8%) |  |
| **Donor type (N (%))** |  |  | <0.001 |
| Living | 112 (38.4%) | 101 (25.8%) |  |
| Deceased | 180 (61.6%) | 290 (74.2%) |  |
| **Donor DCD (N (%))** |  |  | 0.84 |
| No | 102 (56.7%) | 167 (57.6%) |  |
| Yes | 78 (43.3%) | 123 (42.4%) |  |
| **KDPI, median (IQR)** | 28 (13-51) | 27 (12-55) | 0.44 |
| **Donor cause of death (N (%))** |  |  | 0.69 |
| Anoxia | 83 (46.1%) | 149 (51.4%) |  |
| Cerebrovascular/stroke | 27 (15.0%) | 39 (13.4%) |  |
| Head trauma | 63 (35.0%) | 92 (31.7%) |  |
| Central nervous system tumor | 2 (1.1%) | 1 (0.3%) |  |
| Other | 5 (2.8%) | 9 (3.1%) |  |
| **Donor terminal creatinine (mg/dl), median (IQR)** | 0.80 (0.65-1.01) | 0.83 (0.66-1.03) | 0.32 |
| **Donors diabetes (N (%))** |  |  | 0.10 |
| No | 283 (96.9%) | 362 (94.3%) |  |
| Yes | 9 (3.1%) | 22 (5.7%) |  |
| **Donors hypertension (N (%))** |  |  | 0.015 |
| No | 258 (89.0%) | 314 (82.2%) |  |
| Yes | 32 (11.0%) | 68 (17.8%) |  |
| **Donor malignancy (N (%))** |  |  | 0.88 |
| No | 284 (97.3%) | 381 (97.4%) |  |
| Yes | 8 (2.7%) | 10 (2.6%) |  |
| **Immunological characteristics** |  |  |  |
| **Number of HLA mismatches (HLA A,B and DR) (N (%))** |  |  | <0.001 |
| 0 | 26 (8.9%) | 0 (0.0%) |  |
| 1 | 17 (5.8%) | 0 (0.0%) |  |
| 2 | 36 (12.3%) | 4 (1.0%) |  |
| 3 | 91 (31.2%) | 31 (7.9%) |  |
| 4 | 63 (21.6%) | 100 (25.6%) |  |
| 5 | 46 (15.8%) | 166 (42.5%) |  |
| 6 | 13 (4.5%) | 90 (23.0%) |  |
| **HLA mismatches A (N (%))** |  |  | <0.001 |
| 0 | 20 (28%) | 4 (4%) |  |
| 1 | 39 (54%) | 36 (39%) |  |
| 2 | 13 (18%) | 53 (57%) |  |
| **HLA mismatches B (N (%))** |  |  | <0.001 |
| 0 | 11 (15%) | 2 (2%) |  |
| 1 | 26 (36%) | 20 (22%) |  |
| 2 | 35 (49%) | 71 (76%) |  |
| **HLA mismatches DR (N (%))** |  |  | <0.001 |
| 0 | 37 (24.5%) | 12 (6.3%) |  |
| 1 | 88 (58.3%) | 81 (42.9%) |  |
| 2 | 26 (17.2%) | 96 (50.8%) |  |
| **HLA mismatches DQ (N (%))** |  |  | <0.001 |
| 0 | 25 (35%) | 9 (10%) |  |
| 1 | 34 (47%) | 46 (50%) |  |
| 2 | 13 (18%) | 37 (40%) |  |
| **Current flow B cell (N (%))** |  |  | 0.21 |
| < 2 SD compatible | 141 (92.2%) | 182 (91.9%) |  |
| 2-3 SD compatible | 8 (5.2%) | 7 (3.5%) |  |
| weak positive | 0 (0.0%) | 5 (2.5%) |  |
| positive | 4 (2.6%) | 4 (2.0%) |  |
| **Current flow T cell (N (%))** |  |  | 0.089 |
| < 2 SD compatible | 139 (90.8%) | 188 (94.9%) |  |
| 2-3 SD compatible | 7 (4.6%) | 5 (2.5%) |  |
| weak positive | 4 (2.6%) | 0 (0.0%) |  |
| positive | 3 (2.0%) | 5 (2.5%) |  |
| **Induction treatment (N (%))** |  |  |  |
| No Induction | 0 (0%) | 0 (0%) | 1.000 |
| Basiliximab | 42 (14.4%) | 40 (10.2%) | 0.099 |
| Thymoglobulin | 232 (79.5%) | 336 (85.9%) | 0.025 |
| Steroid | 292 (100.0%) | 390 (99.7%) | 0.39 |
| Alemtuzumab | 9 (3.1%) | 12 (3.1%) | 0.99 |
| **Outcomes** |  |  |  |
| **Proportion of Antibody Mediated Rejection of the subset of patient underwent biopsy (N (%))** |  |  | 0.35 |
| No | 73 (78.5%) | 112 (73.2%) |  |
| Yes | 20 (21.5%) | 41 (26.8%) |  |
| **Proportion of rejection of the subset of patient underwent biopsy (N (%))** |  |  | 0.046 |
| No | 63 (67.7%) | 84 (54.9%) |  |
| Yes | 30 (32.3%) | 69 (45.1%) |  |
| **Proportion of T-cell Mediated Rejection of the subset of patient underwent biopsy (N (%))** |  |  | 0.094 |
| No | 68 (73.1%) | 96 (62.7%) |  |
| Yes | 25 (26.9%) | 57 (37.3%) |  |
| **Post-Transplant Donor Specific Antibodies (N (%))** |  |  | 0.023 |
| No | 230 (81.0%) | 285 (73.5%) |  |
| Yes | 54 (19.0%) | 103 (26.5%) |  |
| **Dd-cf-DNA elevation (N (%))** |  |  | <0.001 |
| No | 192 (86.1%) | 227 (74.2%) |  |
| Yes | 31 (13.9%) | 79 (25.8%) |  |
| **Primary outcome (N (%))** |  |  | <0.001 |
| No | 208 (71.2%) | 225 (57.5%) |  |
| Yes | 84 (28.8%) | 166 (42.5%) |  |
| **Delayed Graft Function (N (%))** |  |  | 0.54 |
| No | 269 (92.1%) | 355 (90.8%) |  |
| Yes | 23 (7.9%) | 36 (9.2%) |  |
| **Death (N (%))** |  |  | 0.64 |
| No | 280 (95.9%) | 372 (95.1%) |  |
| Yes | 12 (4.1%) | 19 (4.9%) |  |
| **Graft Loss (N (%))** |  |  | 0.93 |
| No | 285 (97.6%) | 382 (97.7%) |  |
| Yes | 7 (2.4%) | 9 (2.3%) |  |

Values are expressed as mean (standard deviation), median (interquartile range), or number (%). Continuous variables were compared via t-tests or Mann-Whitney U tests. Categorical variables were compared via Chi-square tests.

**Table S2.** Patient characteristics based on PIRCHE-B score

|  | **Low Risk PIRCHE-B Score Group** | **High Risk PIRCHE-B Score Group** | **p-value** |
| --- | --- | --- | --- |
| **N** | 251 (37%) | 432 (63%) |  |
| **Recipients’ characteristics** |  |  |  |
| **Age (years), mean (SD)** | 51.3 (15.4) | 49.2 (14.7) | 0.073 |
| **Gender (N (%))** |  |  | 0.13 |
| male | 144 (57.4%) | 273 (63.2%) |  |
| female | 107 (42.6%) | 159 (36.8%) |  |
| **Race/ethnicity (N (%))** |  |  | 0.001 |
| White | 188 (74.9%) | 272 (63.0%) |  |
| Hispanic | 45 (17.9%) | 82 (19.0%) |  |
| African American | 2 (0.8%) | 13 (3.0%) |  |
| Asian | 6 (2.4%) | 20 (4.6%) |  |
| Other | 9 (3.6%) | 45 (10.4%) |  |
| Unknown | 1 (0.4%) | 0 (0.0%) |  |
| **Body Mass Index (kg/m2), mean (SD)** | 28.4 (5.4) | 28.8 (5.8) | 0.34 |
| **Dialysis vintage (months), median (IQR)** | 28.8 (14.8-51.8) | 35.0 (16.3-58.5) | 0.039 |
| **Cause of kidney failure (N (%))** |  |  | 0.512 |
| Diabetes mellitus | 69 (27%) | 143 (33%) |  |
| Hypertension/Vascular disease | 33 (13%) | 54 (13%) |  |
| Cystic kidney disease | 19 (8%) | 36 (8%) |  |
| Glomerulonephritis | 60 (24%) | 99 (23%) |  |
| Other/unknown | 70 (28%) | 100 (23%) |  |
| **Prior kidney transplant (N (%))** |  |  | <0.001 |
| No | 206 (82.1%) | 409 (94.7%) |  |
| Yes | 45 (17.9%) | 23 (5.3%) |  |
| **Multi-organ transplantation (N (%))** |  |  | 0.022 |
| Liver + kidney | 6 (2.4%) | 9 (2.1%) |  |
| Pancreas + kidney | 1 (0.4%) | 17 (3.9%) |  |
| Heart + kidney | 2 (0.8%) | 9 (2.1%) |  |
| None | 242 (96.4%) | 394 (91.2%) |  |
| Liver +Heart+Kidney | 0 (0.0%) | 3 (0.7%) |  |
| **CMV risk categories (N (%))** |  |  | 0.65 |
| Low | 61 (24.3%) | 111 (25.7%) |  |
| Intermediate | 113 (45.0%) | 185 (42.8%) |  |
| High | 67 (26.7%) | 125 (28.9%) |  |
| Unknow | 10 (4.0%) | 11 (2.5%) |  |
| **Was the donor organ pumped? (N (%))** |  |  | 0.014 |
| No | 81 (34.9%) | 106 (25.8%) |  |
| Yes | 151 (65.1%) | 305 (74.2%) |  |
| **En block kidney (N (%))** | 1 (2%) | 13 (17%) | 0.022 |
| **Dual kidney (N (%))** | 4 (10%) | 11 (14%) | 0.49 |
| **Transplantation characteristics** |  |  |  |
| **Cold ischemic time (hours), median (IQR)** | 12.2 (3.6-20.4) | 13.8 (6.2-20.2) | 0.095 |
| **cPRA (%), median (IQR)** | 0 (0-31) | 0 (0-0) | <0.001 |
| **Donors’ characteristics** |  |  |  |
| **Age (years), mean (SD)** | 38.5 (15.3) | 36.7 (15.9) | 0.16 |
| **Gender (N (%))** |  |  | 0.12 |
| male | 131 (52.2%) | 252 (58.3%) |  |
| female | 120 (47.8%) | 180 (41.7%) |  |
| **Race/ethnicity** |  |  | 0.81 |
| White | 201 (80.1%) | 354 (81.9%) |  |
| African American | 6 (2.4%) | 15 (3.5%) |  |
| Asian | 5 (2.0%) | 8 (1.9%) |  |
| Other | 4 (1.6%) | 6 (1.4%) |  |
| Unknown | 35 (13.9%) | 49 (11.3%) |  |
| **Donor type (N (%))** |  |  | 0.007 |
| Living | 94 (37.5%) | 119 (27.5%) |  |
| Deceased | 157 (62.5%) | 313 (72.5%) |  |
| **Donor DCD (N (%))** |  |  | 0.18 |
| No | 83 (52.9%) | 186 (59.4%) |  |
| Yes | 74 (47.1%) | 127 (40.6%) |  |
| **KDPI, median (IQR)** | 28 (15-53) | 27 (12-53) | 0.72 |
| **Donor cause of death (N (%))** |  |  | 0.95 |
| Anoxia | 76 (48.4%) | 156 (49.8%) |  |
| Cerebrovascular/stroke | 23 (14.6%) | 43 (13.7%) |  |
| Head trauma | 51 (32.5%) | 104 (33.2%) |  |
| Central nervous system tumor | 1 (0.6%) | 2 (0.6%) |  |
| Other | 6 (3.8%) | 8 (2.6%) |  |
| **Donor terminal creatinine (mg/dl), median (IQR)** | 0.8 (0.64-1.07) | 0.82 (0.66-1.00) | 0.77 |
| **Donors diabetes (N (%))** |  |  | 0.57 |
| No | 238 (94.8%) | 407 (95.8%) |  |
| Yes | 13 (5.2%) | 18 (4.2%) |  |
| **Donors hypertension (N (%))** |  |  | 0.27 |
| No | 216 (87.1%) | 356 (84.0%) |  |
| Yes | 32 (12.9%) | 68 (16.0%) |  |
| **Donor malignancy (N (%))** |  |  | 0.85 |
| No | 244 (97.2%) | 421 (97.5%) |  |
| Yes | 7 (2.8%) | 11 (2.5%) |  |
| **Immunological characteristics** |  |  |  |
| **Number of HLA mismatches (HLA A,B and DR) (N (%))** |  |  | <0.001 |
| 0 | 26 (10.4%) | 0 (0.0%) |  |
| 1 | 15 (6.0%) | 2 (0.5%) |  |
| 2 | 36 (14.3%) | 4 (0.9%) |  |
| 3 | 75 (29.9%) | 47 (10.9%) |  |
| 4 | 61 (24.3%) | 102 (23.6%) |  |
| 5 | 27 (10.8%) | 185 (42.8%) |  |
| 6 | 11 (4.4%) | 92 (21.3%) |  |
| **HLA mismatches A (N (%))** |  |  | <0.001 |
| 0 | 18 (26%) | 6 (6%) |  |
| 1 | 37 (54%) | 38 (40%) |  |
| 2 | 14 (20%) | 52 (54%) |  |
| **HLA mismatches B (N (%))** |  |  | 0.002 |
| 0 | 9 (13%) | 4 (4%) |  |
| 1 | 26 (38%) | 20 (21%) |  |
| 2 | 34 (49%) | 72 (75%) |  |
| **HLA mismatches DR (N (%))** |  |  | <0.001 |
| 0 | 41 (31.8%) | 8 (3.8%) |  |
| 1 | 71 (55.0%) | 98 (46.4%) |  |
| 2 | 17 (13.2%) | 105 (49.8%) |  |
| **HLA mismatches DQ (N (%))** |  |  | <0.001 |
| 0 | 31 (45%) | 3 (3%) |  |
| 1 | 34 (49%) | 46 (48%) |  |
| 2 | 4 (6%) | 46 (48%) |  |
| **Current flow B cell (N (%))** |  |  | 0.87 |
| < 2 SD compatible | 123 (93.2%) | 200 (91.3%) |  |
| 2-3 SD compatible | 5 (3.8%) | 10 (4.6%) |  |
| weak positive | 2 (1.5%) | 3 (1.4%) |  |
| positive | 2 (1.5%) | 6 (2.7%) |  |
| **Current flow T cell (N (%))** |  |  | 0.83 |
| < 2 SD compatible | 123 (93.2%) | 204 (93.2%) |  |
| 2-3 SD compatible | 5 (3.8%) | 7 (3.2%) |  |
| weak positive | 2 (1.5%) | 2 (0.9%) |  |
| positive | 2 (1.5%) | 6 (2.7%) |  |
| **Induction treatment (N (%))** |  |  |  |
| No Induction | 0 (0%) | 0 (0%) | 1.000 |
| Basiliximab | 43 (17.1%) | 39 (9.0%) | 0.002 |
| Thymoglobulin | 191 (76.1%) | 377 (87.3%) | <0.001 |
| Steroid | 251 (100.0%) | 431 (99.8%) | 0.446 |
| Alemtuzumab | 9 (3.6%) | 12 (2.8%) | 0.555 |
| **Outcomes** |  |  |  |
| **Proportion of Antibody Mediated Rejection of the subset of patient underwent biopsy (N (%))** |  |  | 0.010 |
| No | 73 (84.9%) | 112 (70.0%) |  |
| Yes | 13 (15.1%) | 48 (30.0%) |  |
| **Proportion of rejection of the subset of patient underwent biopsy (N (%))** |  |  | 0.009 |
| No | 61 (70.9%) | 86 (53.8%) |  |
| Yes | 25 (29.1%) | 74 (46.2%) |  |
| **Proportion of T-cell Mediated Rejection of the subset of patient underwent biopsy (N (%))** |  |  | 0.11 |
| No | 63 (73.3%) | 101 (63.1%) |  |
| Yes | 23 (26.7%) | 59 (36.9%) |  |
| **Post-Transplant Donor Specific Antibodies (N (%))** |  |  | <0.001 |
| No | 209 (86.0%) | 306 (71.3%) |  |
| Yes | 34 (14.0%) | 123 (28.7%) |  |
| **Dd-cf-DNA elevation (N (%))** |  |  | 0.004 |
| No | 161 (86.1%) | 258 (75.4%) |  |
| Yes | 26 (13.9%) | 84 (24.6%) |  |
| **Primary outcome (N (%))** |  |  | <0.001 |
| No | 192 (76.5%) | 241 (55.8%) |  |
| Yes | 59 (23.5%) | 191 (44.2%) |  |
| **Delayed Graft Function (N (%))** |  |  | 0.51 |
| No | 227 (90.4%) | 397 (91.9%) |  |
| Yes | 24 (9.6%) | 35 (8.1%) |  |
| **Death (N (%))** |  |  | 0.82 |
| No | 239 (95.2%) | 413 (95.6%) |  |
| Yes | 12 (4.8%) | 19 (4.4%) |  |
| **Graft Loss (N (%))** |  |  | 0.95 |
| No | 245 (97.6%) | 422 (97.7%) |  |
| Yes | 6 (2.4%) | 10 (2.3%) |  |

Values are expressed as mean (standard deviation), median (interquartile range), or number (%). Continuous variables were compared via t-tests or Mann-Whitney U tests. Categorical variables were compared via Chi-square tests.

**Table S3:** Performance characteristics table of PIRCHE-T2 for primary outcome

| Cutpoint | Sensitivity | Specificity | Overall % correctly classified | LR+ | LR- |
| --- | --- | --- | --- | --- | --- |
| ( >= 0 ) | 100.00% | 0.00% | 36.60% | 1 |  |
| ( >= 1 ) | 99.60% | 4.85% | 39.53% | 1.0468 | 0.0825 |
| ( >= 5 ) | 99.60% | 5.08% | 39.68% | 1.0493 | 0.0787 |
| ( >= 7 ) | 99.60% | 5.31% | 39.82% | 1.0519 | 0.0753 |
| ( >= 8 ) | 99.20% | 5.31% | 39.68% | 1.0476 | 0.1506 |
| ( >= 9 ) | 99.20% | 6.00% | 40.12% | 1.0554 | 0.1332 |
| ( >= 10 ) | 98.80% | 6.00% | 39.97% | 1.0511 | 0.1998 |
| ( >= 11 ) | 98.40% | 6.24% | 39.97% | 1.0494 | 0.2566 |
| ( >= 12 ) | 98.40% | 6.93% | 40.41% | 1.0573 | 0.2309 |
| ( >= 13 ) | 98.40% | 7.39% | 40.70% | 1.0625 | 0.2165 |
| ( >= 14 ) | 98.40% | 7.62% | 40.85% | 1.0652 | 0.2099 |
| ( >= 15 ) | 98.40% | 7.85% | 41.00% | 1.0678 | 0.2038 |
| ( >= 16 ) | 98.00% | 8.31% | 41.14% | 1.0689 | 0.2406 |
| ( >= 17 ) | 98.00% | 8.55% | 41.29% | 1.0716 | 0.2341 |
| ( >= 18 ) | 97.60% | 9.47% | 41.73% | 1.0781 | 0.2535 |
| ( >= 18.26 ) | 97.60% | 9.70% | 41.87% | 1.0808 | 0.2474 |
| ( >= 19 ) | 97.60% | 9.93% | 42.02% | 1.0836 | 0.2417 |
| ( >= 19.04 ) | 96.80% | 10.39% | 42.02% | 1.0803 | 0.3079 |
| ( >= 20 ) | 96.40% | 10.39% | 41.87% | 1.0758 | 0.3464 |
| ( >= 21 ) | 95.60% | 11.09% | 42.02% | 1.0752 | 0.3969 |
| ( >= 22 ) | 95.20% | 12.01% | 42.46% | 1.0819 | 0.3997 |
| ( >= 23 ) | 95.20% | 12.93% | 43.05% | 1.0934 | 0.3711 |
| ( >= 24 ) | 93.60% | 13.16% | 42.61% | 1.0779 | 0.4862 |
| ( >= 24.96 ) | 93.60% | 13.63% | 42.90% | 1.0837 | 0.4697 |
| ( >= 25 ) | 93.20% | 13.63% | 42.75% | 1.079 | 0.4991 |
| ( >= 26 ) | 92.80% | 13.86% | 42.75% | 1.0773 | 0.5196 |
| ( >= 27 ) | 92.40% | 14.32% | 42.90% | 1.0784 | 0.5308 |
| ( >= 28 ) | 91.60% | 14.78% | 42.90% | 1.0749 | 0.5683 |
| ( >= 29 ) | 90.80% | 15.47% | 43.05% | 1.0742 | 0.5946 |
| ( >= 30 ) | 90.00% | 15.70% | 42.90% | 1.0677 | 0.6368 |
| ( >= 31 ) | 88.80% | 16.40% | 42.90% | 1.0622 | 0.683 |
| ( >= 31.71 ) | 88.40% | 18.01% | 43.78% | 1.0782 | 0.6439 |
| ( >= 32 ) | 88.40% | 18.24% | 43.92% | 1.0813 | 0.6358 |
| ( >= 33 ) | 87.20% | 19.63% | 44.36% | 1.085 | 0.652 |
| ( >= 33.22 ) | 86.40% | 20.79% | 44.80% | 1.0907 | 0.6543 |
| ( >= 34 ) | 86.40% | 21.02% | 44.95% | 1.0939 | 0.6471 |
| ( >= 35 ) | 85.20% | 21.94% | 45.10% | 1.0915 | 0.6746 |
| ( >= 35.65 ) | 83.60% | 22.40% | 44.80% | 1.0773 | 0.7321 |
| ( >= 36 ) | 83.60% | 22.63% | 44.95% | 1.0806 | 0.7246 |
| ( >= 36.47 ) | 83.20% | 24.48% | 45.97% | 1.1017 | 0.6863 |
| ( >= 37 ) | 83.20% | 24.71% | 46.12% | 1.1051 | 0.6799 |
| ( >= 38 ) | 82.00% | 26.79% | 47.00% | 1.1201 | 0.6719 |
| ( >= 38.22 ) | 81.60% | 27.48% | 47.29% | 1.1252 | 0.6695 |
| ( >= 38.54 ) | 81.60% | 27.71% | 47.44% | 1.1288 | 0.6639 |
| ( >= 39 ) | 81.20% | 27.71% | 47.29% | 1.1233 | 0.6784 |
| ( >= 39.24 ) | 80.80% | 29.79% | 48.46% | 1.1509 | 0.6445 |
| ( >= 40 ) | 80.80% | 30.02% | 48.61% | 1.1547 | 0.6395 |
| ( >= 40.25 ) | 80.40% | 30.95% | 49.05% | 1.1643 | 0.6333 |
| ( >= 41 ) | 80.40% | 31.18% | 49.19% | 1.1682 | 0.6287 |
| ( >= 41.11 ) | 78.80% | 31.64% | 48.90% | 1.1527 | 0.67 |
| ( >= 42 ) | 78.80% | 31.87% | 49.05% | 1.1566 | 0.6652 |
| ( >= 43 ) | 78.40% | 33.49% | 49.93% | 1.1787 | 0.645 |
| ( >= 44 ) | 76.80% | 34.18% | 49.78% | 1.1668 | 0.6788 |
| ( >= 45 ) | 76.00% | 34.41% | 49.63% | 1.1587 | 0.6974 |
| ( >= 45.35 ) | 74.40% | 35.57% | 49.78% | 1.1547 | 0.7198 |
| ( >= 45.78 ) | 74.40% | 35.80% | 49.93% | 1.1588 | 0.7151 |
| ( >= 46 ) | 74.00% | 35.80% | 49.78% | 1.1526 | 0.7263 |
| ( >= 47 ) | 72.80% | 37.18% | 50.22% | 1.1589 | 0.7315 |
| ( >= 48 ) | 72.80% | 38.34% | 50.95% | 1.1806 | 0.7095 |
| ( >= 48.19 ) | 72.40% | 39.26% | 51.39% | 1.192 | 0.703 |
| ( >= 48.23 ) | 72.40% | 39.49% | 51.54% | 1.1965 | 0.6989 |
| ( >= 49 ) | 72.40% | 39.72% | 51.68% | 1.2011 | 0.6948 |
| ( >= 49.32 ) | 71.20% | 41.80% | 52.56% | 1.2234 | 0.689 |
| ( >= 50 ) | 70.80% | 41.80% | 52.42% | 1.2165 | 0.6985 |
| ( >= 50.08 ) | 68.40% | 44.11% | 53.00% | 1.2239 | 0.7164 |
| ( >= 51 ) | 68.40% | 44.34% | 53.15% | 1.2289 | 0.7126 |
| ( >= 51.43 ) | 67.60% | 46.19% | 54.03% | 1.2563 | 0.7015 |
| ( >= 52 ) | 67.60% | 46.42% | 54.17% | 1.2617 | 0.698 |
| ( >= 53 ) | 66.40% | 48.04% | 54.76% | 1.2778 | 0.6995 |
| ( >= 54 ) | 64.40% | 48.73% | 54.47% | 1.2561 | 0.7306 |
| ( >= 54.3 ) | 63.60% | 49.65% | 54.76% | 1.2632 | 0.7331 |
| ( >= 55 ) | 63.20% | 49.65% | 54.61% | 1.2553 | 0.7411 |
| ( >= 55.59 ) | 61.20% | 51.27% | 54.90% | 1.2559 | 0.7568 |
| ( >= 55.89 ) | 60.80% | 51.27% | 54.76% | 1.2477 | 0.7646 |
| ( >= 56 ) | 60.80% | 51.50% | 54.90% | 1.2536 | 0.7611 |
| ( >= 57 ) | 60.00% | 53.12% | 55.64% | 1.2798 | 0.753 |
| ( >= 57.04 ) | 56.00% | 54.27% | 54.90% | 1.2246 | 0.8107 |
| ( >= 57.83 ) | 55.60% | 54.27% | 54.76% | 1.2159 | 0.8181 |
| ( >= 58 ) | 55.20% | 54.27% | 54.61% | 1.2072 | 0.8255 |
| ( >= 58.94 ) | 53.60% | 55.43% | 54.76% | 1.2025 | 0.8371 |
| ( >= 59 ) | 53.60% | 55.66% | 54.90% | 1.2088 | 0.8337 |
| ( >= 59.83 ) | 52.40% | 57.27% | 55.49% | 1.2264 | 0.8311 |
| ( >= 60 ) | 52.40% | 57.51% | 55.64% | 1.2331 | 0.8277 |
| ( >= 61 ) | 50.80% | 59.12% | 56.08% | 1.2427 | 0.8322 |
| ( >= 61.79 ) | 49.60% | 61.66% | 57.25% | 1.2938 | 0.8173 |
| ( >= 61.96 ) | 49.60% | 61.89% | 57.39% | 1.3016 | 0.8143 |
| ( >= 62 ) | 49.20% | 61.89% | 57.25% | 1.2911 | 0.8208 |
| ( >= 63 ) | 47.20% | 62.82% | 57.10% | 1.2694 | 0.8405 |
| ( >= 64 ) | 46.40% | 63.51% | 57.25% | 1.2716 | 0.844 |
| ( >= 64.81 ) | 46.00% | 63.97% | 57.39% | 1.2768 | 0.8441 |
| ( >= 65 ) | 46.00% | 64.20% | 57.54% | 1.285 | 0.8411 |
| ( >= 66 ) | 44.40% | 65.36% | 57.69% | 1.2817 | 0.8507 |
| ( >= 66.94 ) | 42.40% | 66.28% | 57.54% | 1.2575 | 0.869 |
| ( >= 67 ) | 42.40% | 66.51% | 57.69% | 1.2662 | 0.866 |
| ( >= 67.07 ) | 41.20% | 67.67% | 57.98% | 1.2743 | 0.869 |
| ( >= 68 ) | 40.80% | 67.67% | 57.83% | 1.2619 | 0.8749 |
| ( >= 68.08 ) | 38.40% | 69.28% | 57.98% | 1.2502 | 0.8891 |
| ( >= 68.79 ) | 38.00% | 69.28% | 57.83% | 1.2371 | 0.8949 |
| ( >= 69 ) | 38.00% | 69.52% | 57.98% | 1.2465 | 0.8919 |
| ( >= 69.96 ) | 37.60% | 70.67% | 58.57% | 1.282 | 0.883 |
| ( >= 70 ) | 37.60% | 70.90% | 58.71% | 1.2921 | 0.8801 |
| ( >= 70.5 ) | 36.40% | 72.29% | 59.15% | 1.3134 | 0.8798 |
| ( >= 71 ) | 36.40% | 72.52% | 59.30% | 1.3245 | 0.877 |
| ( >= 71.3 ) | 35.60% | 73.21% | 59.44% | 1.3289 | 0.8797 |
| ( >= 71.68 ) | 35.60% | 73.44% | 59.59% | 1.3404 | 0.8769 |
| ( >= 72 ) | 35.20% | 73.44% | 59.44% | 1.3254 | 0.8823 |
| ( >= 72.47 ) | 34.40% | 73.67% | 59.30% | 1.3066 | 0.8904 |
| ( >= 73 ) | 34.40% | 73.90% | 59.44% | 1.3182 | 0.8877 |
| ( >= 73.8 ) | 32.40% | 75.06% | 59.44% | 1.299 | 0.9006 |
| ( >= 73.94 ) | 32.40% | 75.29% | 59.59% | 1.3111 | 0.8979 |
| ( >= 74 ) | 32.40% | 75.52% | 59.74% | 1.3235 | 0.8951 |
| ( >= 75 ) | 31.60% | 76.67% | 60.18% | 1.3547 | 0.8921 |
| ( >= 76 ) | 30.40% | 77.14% | 60.03% | 1.3296 | 0.9023 |
| ( >= 77 ) | 28.00% | 77.83% | 59.59% | 1.2629 | 0.9251 |
| ( >= 78 ) | 26.40% | 79.21% | 59.88% | 1.2701 | 0.9291 |
| ( >= 78.02 ) | 25.20% | 80.14% | 60.03% | 1.2688 | 0.9334 |
| ( >= 79 ) | 24.80% | 80.14% | 59.88% | 1.2487 | 0.9384 |
| ( >= 80 ) | 24.40% | 81.29% | 60.47% | 1.3043 | 0.93 |
| ( >= 81 ) | 22.00% | 81.99% | 60.03% | 1.2213 | 0.9514 |
| ( >= 81.83 ) | 19.60% | 83.60% | 60.18% | 1.1953 | 0.9617 |
| ( >= 82 ) | 19.60% | 83.83% | 60.32% | 1.2124 | 0.959 |
| ( >= 83 ) | 18.00% | 84.53% | 60.18% | 1.1633 | 0.9701 |
| ( >= 83.34 ) | 17.60% | 85.45% | 60.61% | 1.2097 | 0.9643 |
| ( >= 83.69 ) | 17.60% | 85.68% | 60.76% | 1.2292 | 0.9617 |
| ( >= 84 ) | 17.60% | 85.91% | 60.91% | 1.2493 | 0.9591 |
| ( >= 85 ) | 16.40% | 86.14% | 60.61% | 1.1835 | 0.9705 |
| ( >= 85.33 ) | 15.20% | 87.07% | 60.76% | 1.1753 | 0.974 |
| ( >= 86 ) | 14.80% | 87.07% | 60.61% | 1.1444 | 0.9786 |
| ( >= 86.17 ) | 14.00% | 87.30% | 60.47% | 1.1022 | 0.9851 |
| ( >= 87 ) | 13.60% | 87.30% | 60.32% | 1.0707 | 0.9897 |
| ( >= 88 ) | 13.60% | 88.22% | 60.91% | 1.1547 | 0.9794 |
| ( >= 89 ) | 13.60% | 88.68% | 61.20% | 1.2018 | 0.9742 |
| ( >= 89.31 ) | 11.60% | 89.38% | 60.91% | 1.0919 | 0.9891 |
| ( >= 90 ) | 11.60% | 89.61% | 61.05% | 1.1162 | 0.9865 |
| ( >= 91 ) | 10.40% | 90.53% | 61.20% | 1.0983 | 0.9897 |
| ( >= 91.07 ) | 10.00% | 91.22% | 61.49% | 1.1395 | 0.9866 |
| ( >= 92 ) | 9.60% | 91.22% | 61.35% | 1.0939 | 0.991 |
| ( >= 93 ) | 9.60% | 91.69% | 61.64% | 1.1547 | 0.986 |
| ( >= 94 ) | 9.20% | 92.61% | 62.08% | 1.2449 | 0.9805 |
| ( >= 95 ) | 9.20% | 92.84% | 62.23% | 1.285 | 0.978 |
| ( >= 95.94 ) | 8.40% | 93.30% | 62.23% | 1.2542 | 0.9818 |
| ( >= 96 ) | 8.00% | 93.30% | 62.08% | 1.1945 | 0.986 |
| ( >= 97 ) | 8.00% | 93.53% | 62.23% | 1.2371 | 0.9836 |
| ( >= 98 ) | 6.40% | 93.76% | 61.79% | 1.0264 | 0.9982 |
| ( >= 99 ) | 6.00% | 94.92% | 62.37% | 1.1809 | 0.9903 |
| ( >= 100 ) | 5.60% | 95.61% | 62.66% | 1.2762 | 0.9873 |
| ( >= 101 ) | 5.60% | 95.84% | 62.81% | 1.3471 | 0.9849 |
| ( >= 101.13 ) | 5.60% | 96.30% | 63.10% | 1.5155 | 0.9802 |
| ( >= 102 ) | 5.60% | 96.54% | 63.25% | 1.6165 | 0.9779 |
| ( >= 103 ) | 4.40% | 96.77% | 62.96% | 1.3609 | 0.9879 |
| ( >= 105 ) | 4.00% | 96.77% | 62.81% | 1.2371 | 0.9921 |
| ( >= 106 ) | 4.00% | 97.00% | 62.96% | 1.3323 | 0.9897 |
| ( >= 107 ) | 3.60% | 97.23% | 62.96% | 1.299 | 0.9915 |
| ( >= 108 ) | 3.20% | 97.23% | 62.81% | 1.1547 | 0.9956 |
| ( >= 109 ) | 3.20% | 97.46% | 62.96% | 1.2596 | 0.9932 |
| ( >= 110 ) | 2.40% | 97.46% | 62.66% | 0.9447 | 1.0014 |
| ( >= 111 ) | 2.00% | 97.69% | 62.66% | 0.866 | 1.0032 |
| ( >= 112 ) | 1.60% | 98.15% | 62.81% | 0.866 | 1.0025 |
| ( >= 113 ) | 1.20% | 98.38% | 62.81% | 0.7423 | 1.0042 |
| ( >= 114 ) | 1.20% | 98.85% | 63.10% | 1.0392 | 0.9995 |
| ( >= 116 ) | 1.20% | 99.08% | 63.25% | 1.299 | 0.9972 |
| ( >= 118 ) | 1.20% | 99.31% | 63.40% | 1.732 | 0.9949 |
| ( >= 120.6 ) | 0.80% | 99.31% | 63.25% | 1.1547 | 0.9989 |
| ( >= 121 ) | 0.80% | 99.54% | 63.40% | 1.732 | 0.9966 |
| ( >= 128 ) | 0.80% | 99.77% | 63.54% | 3.4641 | 0.9943 |
| ( >= 132 ) | 0.40% | 99.77% | 63.40% | 1.732 | 0.9983 |
| ( >= 137 ) | 0.40% | 100.00% | 63.54% |  | 0.996 |
| ( > 137 ) | 0.00% | 100.00% | 63.40% |  | 1 |

LR: Likelihood ratio

**Table S4:** Performance characteristics table of PIRCHE-B for primary outcome

| Cutpoint | Sensitivity | Specificity | Overall % correctly classified | LR+ | LR- |
| --- | --- | --- | --- | --- | --- |
| ( >= 0 ) | 100.00% | 0.00% | 36.60% | 1 |  |
| ( >= 1 ) | 99.60% | 5.31% | 39.82% | 1.0519 | 0.0753 |
| ( >= 2 ) | 98.00% | 6.47% | 39.97% | 1.0478 | 0.3093 |
| ( >= 3 ) | 97.20% | 8.08% | 40.70% | 1.0575 | 0.3464 |
| ( >= 4 ) | 95.60% | 10.85% | 41.87% | 1.0724 | 0.4054 |
| ( >= 5 ) | 94.80% | 13.16% | 43.05% | 1.0917 | 0.395 |
| ( >= 6 ) | 92.80% | 16.40% | 44.36% | 1.11 | 0.4391 |
| ( >= 6.69 ) | 91.20% | 21.71% | 47.14% | 1.1649 | 0.4054 |
| ( >= 7 ) | 91.20% | 21.94% | 47.29% | 1.1683 | 0.4011 |
| ( >= 8 ) | 88.00% | 27.25% | 49.49% | 1.2097 | 0.4403 |
| ( >= 9 ) | 84.80% | 31.64% | 51.10% | 1.2405 | 0.4804 |
| ( >= 9.02 ) | 80.00% | 38.57% | 53.73% | 1.3023 | 0.5186 |
| ( >= 9.51 ) | 79.60% | 38.57% | 53.59% | 1.2957 | 0.5289 |
| ( >= 10 ) | 79.60% | 38.80% | 53.73% | 1.3006 | 0.5258 |
| ( >= 10.71 ) | 76.40% | 44.11% | 55.93% | 1.367 | 0.535 |
| ( >= 11 ) | 76.40% | 44.34% | 56.08% | 1.3727 | 0.5322 |
| ( >= 12 ) | 71.60% | 48.96% | 57.25% | 1.4028 | 0.5801 |
| ( >= 13 ) | 66.00% | 53.58% | 58.13% | 1.4218 | 0.6346 |
| ( >= 13.17 ) | 59.60% | 58.43% | 58.86% | 1.4337 | 0.6914 |
| ( >= 14 ) | 59.20% | 58.43% | 58.71% | 1.4241 | 0.6983 |
| ( >= 15 ) | 51.60% | 61.89% | 58.13% | 1.3541 | 0.782 |
| ( >= 16 ) | 45.60% | 66.74% | 59.00% | 1.3712 | 0.8151 |
| ( >= 17 ) | 40.40% | 71.13% | 59.88% | 1.3995 | 0.8379 |
| ( >= 17.24 ) | 34.40% | 75.06% | 60.18% | 1.3792 | 0.874 |
| ( >= 18 ) | 34.00% | 75.06% | 60.03% | 1.3631 | 0.8793 |
| ( >= 19 ) | 32.00% | 80.14% | 62.52% | 1.6112 | 0.8485 |
| ( >= 20 ) | 28.80% | 82.68% | 62.96% | 1.6627 | 0.8612 |
| ( >= 21 ) | 24.00% | 85.68% | 63.10% | 1.6761 | 0.887 |
| ( >= 21.07 ) | 18.00% | 89.61% | 63.40% | 1.732 | 0.9151 |
| ( >= 22 ) | 17.60% | 89.61% | 63.25% | 1.6935 | 0.9196 |
| ( >= 23 ) | 14.40% | 91.45% | 63.25% | 1.6852 | 0.936 |
| ( >= 24 ) | 10.80% | 92.84% | 62.81% | 1.5085 | 0.9608 |
| ( >= 25 ) | 8.80% | 94.69% | 63.25% | 1.6567 | 0.9632 |
| ( >= 26 ) | 6.80% | 96.54% | 63.69% | 1.9629 | 0.9654 |
| ( >= 27 ) | 4.80% | 97.92% | 63.84% | 2.3093 | 0.9722 |
| ( >= 28 ) | 4.40% | 98.61% | 64.13% | 3.1753 | 0.9694 |
| ( >= 30 ) | 2.80% | 99.08% | 63.84% | 3.031 | 0.9811 |
| ( >= 31 ) | 2.00% | 99.08% | 63.54% | 2.165 | 0.9891 |
| ( >= 32 ) | 1.60% | 99.54% | 63.69% | 3.464 | 0.9886 |
| ( >= 33 ) | 1.20% | 99.54% | 63.54% | 2.598 | 0.9926 |
| ( >= 34 ) | 0.80% | 99.77% | 63.54% | 3.4641 | 0.9943 |
| ( >= 50 ) | 0.40% | 99.77% | 63.40% | 1.732 | 0.9983 |
| ( >= 54 ) | 0.00% | 99.77% | 63.25% | 0 | 1.0023 |
| ( > 54 ) | 0.00% | 100.00% | 63.40% |  | 1 |

LR: Likelihood ratio

**Table S5:** Patient characteristics of the DSA cohort based on PIRCHE-T2 score

|  | **Low Risk PIRCHE-T2 Score Group** | **High Risk PIRCHE-T2 Score Group** | **p-value** |
| --- | --- | --- | --- |
| **N** | 475 (71%) | 198 (29%) |  |
| **Recipients’ characteristics** |  |  |  |
| **Age (years), mean (SD)** | 49.6 (15.3) | 51.1 (14.2) | 0.23 |
| **Gender (N (%))** |  |  | 0.061 |
| male | 280 (58.9%) | 132 (66.7%) |  |
| female | 195 (41.1%) | 66 (33.3%) |  |
| **Race/ethnicity (N (%))** |  |  | 0.025 |
| White | 336 (70.7%) | 115 (58.1%) |  |
| Hispanic | 83 (17.5%) | 43 (21.7%) |  |
| African American | 8 (1.7%) | 7 (3.5%) |  |
| Asian | 16 (3.4%) | 10 (5.1%) |  |
| Other | 31 (6.5%) | 23 (11.6%) |  |
| Unknown | 1 (0.2%) | 0 (0.0%) |  |
| **Body Mass Index (kg/m2), mean (SD)** | 28.6 (5.8) | 29.05586 (5.378603) | 0.32 |
| **Dialysis vintage (months), median (IQR)** | 30.5 (14.4-54.0) | 36.2 (18.7-62.7) | 0.033 |
| **Prior kidney transplant (N (%))** |  |  | 0.005 |
| No | 417 (87.8%) | 188 (94.9%) |  |
| Yes | 58 (12.2%) | 10 (5.1%) |  |
| **Multi-organ transplantation (N (%))** |  |  | 0.37 |
| Liver + kidney | 13 (2.7%) | 2 (1.0%) |  |
| Pancreas + kidney | 12 (2.5%) | 6 (3.0%) |  |
| Heart + kidney | 7 (1.5%) | 4 (2.0%) |  |
| None | 442 (93.1%) | 184 (92.9%) |  |
| Liver +Heart+Kidney | 1 (0.2%) | 2 (1.0%) |  |
| **CMV risk categories (N (%))** |  |  | 0.34 |
| Low | 119 (25.1%) | 49 (24.7%) |  |
| Intermediate | 200 (42.1%) | 94 (47.5%) |  |
| High | 139 (29.3%) | 52 (26.3%) |  |
| Unknow | 17 (3.6%) | 3 (1.5%) |  |
| **Was the donor organ pumped? (N (%))** |  |  | 0.013 |
| No | 139 (31.3%) | 41 (21.6%) |  |
| Yes | 305 (68.7%) | 149 (78.4%) |  |
| **En block kidney (N (%))** | 7 (9%) | 7 (20%) | 0.090 |
| **Dual kidney (N (%))** | 9 (11%) | 6 (17%) | 0.39 |
| **Transplantation characteristics** |  |  |  |
| **Cold ischemic time (hours), median (IQR)** | 12.7 (4.4-20.6) | 14.3 (7.2-19.1) | 0.22 |
| **cPRA (%), median (IQR)** | 0 (0-2) | 0 (0-0) | 0.042 |
| **Donors’ characteristics** |  |  |  |
| **Age (years), mean (SD)** | 37.6 (15.7) | 36.8 (16.1) | 0.58 |
| **Gender (N (%))** |  |  | 0.082 |
| male | 258 (54.3%) | 122 (61.6%) |  |
| female | 217 (45.7%) | 76 (38.4%) |  |
| **Race/ethnicity** |  |  | 0.58 |
| White | 387 (81.5%) | 158 (79.8%) |  |
| African American | 12 (2.5%) | 9 (4.5%) |  |
| Asian | 8 (1.7%) | 5 (2.5%) |  |
| Other | 8 (1.7%) | 2 (1.0%) |  |
| Unknown | 60 (12.6%) | 24 (12.1%) |  |
| **Donor type (N (%))** |  |  | 0.014 |
| Living | 158 (33.3%) | 47 (23.7%) |  |
| Deceased | 317 (66.7%) | 151 (76.3%) |  |
| **Donor DCD (N (%))** |  |  | 0.27 |
| No | 187 (59.0%) | 81 (53.6%) |  |
| Yes | 130 (41.0%) | 70 (46.4%) |  |
| **KDPI, median (IQR)** | 27 (12-51) | 28 (13-59) | 0.23 |
| **Donor cause of death (N (%))** |  |  | 0.88 |
| Anoxia | 156 (49.2%) | 74 (49.0%) |  |
| Cerebrovascular/stroke | 43 (13.6%) | 23 (15.2%) |  |
| Head trauma | 108 (34.1%) | 47 (31.1%) |  |
| Central nervous system tumor | 2 (0.6%) | 1 (0.7%) |  |
| Other | 8 (2.5%) | 6 (4.0%) |  |
| **Donor terminal creatinine (mg/dl), median (IQR)** | 0.82 (0.65-1.03) | 0.81 (0.62-1.02) | 0.50 |
| **Donors diabetes (N (%))** |  |  | 0.46 |
| No | 449 (95.7%) | 186 (94.4%) |  |
| Yes | 20 (4.3%) | 11 (5.6%) |  |
| **Donors hypertension (N (%))** |  |  | 0.021 |
| No | 406 (87.1%) | 157 (80.1%) |  |
| Yes | 60 (12.9%) | 39 (19.9%) |  |
| **Donor malignancy (N (%))** |  |  | 0.37 |
| No | 464 (97.7%) | 191 (96.5%) |  |
| Yes | 11 (2.3%) | 7 (3.5%) |  |
| **Immunological characteristics** |  |  |  |
| **Number of HLA mismatches (HLA A,B and DR) (N (%))** |  |  | <0.001 |
| 0 | 20 (4.2%) | 0 (0.0%) |  |
| 1 | 17 (3.6%) | 0 (0.0%) |  |
| 2 | 40 (8.4%) | 0 (0.0%) |  |
| 3 | 112 (23.6%) | 9 (4.5%) |  |
| 4 | 122 (25.7%) | 39 (19.7%) |  |
| 5 | 116 (24.4%) | 95 (48.0%) |  |
| 6 | 48 (10.1%) | 55 (27.8%) |  |
| **HLA mismatches A (N (%))** |  |  | <0.001 |
| 0 | 22 (18.2%) | 0 (0.0%) |  |
| 1 | 61 (50.4%) | 14 (33.3%) |  |
| 2 | 38 (31.4%) | 28 (66.7%) |  |
| **HLA mismatches B (N (%))** |  |  | 0.002 |
| 0 | 13 (10.7%) | 0 (0.0%) |  |
| 1 | 40 (33.1%) | 6 (14.3%) |  |
| 2 | 68 (56.2%) | 36 (85.7%) |  |
| **HLA mismatches DR (N (%))** |  |  | <0.001 |
| 0 | 42 (17.3%) | 6 (6.5%) |  |
| 1 | 134 (55.1%) | 35 (38.0%) |  |
| 2 | 67 (27.6%) | 51 (55.4%) |  |
| **HLA mismatches DQ (N (%))** |  |  | 0.003 |
| 0 | 32 (26.4%) | 2 (4.9%) |  |
| 1 | 60 (49.6%) | 20 (48.8%) |  |
| 2 | 29 (24.0%) | 19 (46.3%) |  |
| **Current flow B cell (N (%))** |  |  | 0.80 |
| < 2 SD compatible | 229 (92.3%) | 90 (91.8%) |  |
| 2-3 SD compatible | 11 (4.4%) | 4 (4.1%) |  |
| weak positive | 2 (0.8%) | 2 (2.0%) |  |
| positive | 6 (2.4%) | 2 (2.0%) |  |
| **Current flow T cell (N (%))** |  |  | 0.63 |
| < 2 SD compatible | 229 (92.3%) | 93 (94.9%) |  |
| 2-3 SD compatible | 9 (3.6%) | 3 (3.1%) |  |
| weak positive | 4 (1.6%) | 0 (0.0%) |  |
| positive | 6 (2.4%) | 2 (2.0%) |  |
| **Induction treatment (N (%))** |  |  |  |
| No Induction | 0 (0%) | 0 (0%) | 1.000 |
| Basiliximab | 58 (12.2%) | 19 (9.6%) | 0.33 |
| Thymoglobulin | 390 (82.1%) | 173 (87.4%) | 0.092 |
| Steroid | 475 (100.0%) | 197 (99.5%) | 0.12 |
| Alemtuzumab | 15 (3.2%) | 6 (3.0%) | 0.93 |
| **Outcomes** |  |  |  |
| **Proportion of Antibody Mediated Rejection of the subset of patient underwent biopsy (N (%))** |  |  | 0.10 |
| No | 132 (78.1%) | 52 (68.4%) |  |
| Yes | 37 (21.9%) | 24 (31.6%) |  |
| **Proportion of rejection of the subset of patient underwent biopsy (N (%))** |  |  | 0.14 |
| No | 106 (62.7%) | 40 (52.6%) |  |
| Yes | 63 (37.3%) | 36 (47.4%) |  |
| **Proportion of T-cell Mediated Rejection of the subset of patient underwent biopsy (N (%))** |  |  | 0.055 |
| No | 119 (70.4%) | 44 (57.9%) |  |
| Yes | 50 (29.6%) | 32 (42.1%) |  |
| **Post-Transplant Donor Specific Antibodies (N (%))** |  |  | 0.003 |
| No | 378 (79.7%) | 137 (69.2%) |  |
| Yes | 96 (20.3%) | 61 (30.8%) |  |
| **Dd-cf-DNA elevation (N (%))** |  |  | 0.13 |
| No | 291 (80.6%) | 118 (74.7%) |  |
| Yes | 70 (19.4%) | 40 (25.3%) |  |
| **Primary outcome (N (%))** |  |  | 0.029 |
| No | 311 (65.5%) | 112 (56.6%) |  |
| Yes | 164 (34.5%) | 86 (43.4%) |  |
| **Delayed Graft Function (N (%))** |  |  | 0.78 |
| No | 435 (91.6%) | 180 (90.9%) |  |
| Yes | 40 (8.4%) | 18 (9.1%) |  |
| **Death (N (%))** |  |  | 0.12 |
| No | 457 (96.2%) | 185 (93.4%) |  |
| Yes | 18 (3.8%) | 13 (6.6%) |  |
| **Graft Loss (N (%))** |  |  | 0.81 |
| No | 464 (97.7%) | 194 (98.0%) |  |
| Yes | 11 (2.3%) | 4 (2.0%) |  |

Values are expressed as mean (standard deviation), median (interquartile range), or number (%). Continuous variables were compared via t-tests or Mann-Whitney U tests. Categorical variables were compared via Chi-square tests.

**Table S6:** Patient characteristics of the DSA cohort based on PIRCHE-B score

|  | **Low Risk PIRCHE-B Score Group** | **High Risk PIRCHE-B Score Group** | **p-value** |
| --- | --- | --- | --- |
| **N** | 310 (46%) | 363 (54%) |  |
| **Recipients’ characteristics** |  |  |  |
| **Age (years), mean (SD)** | 50.6 (15.4) | 49.6 (14.7) | 0.38 |
| **Gender (N (%))** |  |  | 0.087 |
| male | 179 (57.7%) | 233 (64.2%) |  |
| female | 131 (42.3%) | 130 (35.8%) |  |
| **Race/ethnicity (N (%))** |  |  | 0.051 |
| White | 223 (71.9%) | 228 (62.8%) |  |
| Hispanic | 55 (17.7%) | 71 (19.6%) |  |
| African American | 6 (1.9%) | 9 (2.5%) |  |
| Asian | 9 (2.9%) | 17 (4.7%) |  |
| Other | 16 (5.2%) | 38 (10.5%) |  |
| Unknown | 1 (0.3%) | 0 (0.0%) |  |
| **Body Mass Index (kg/m2), mean (SD)** | 28.4 (5.5) | 29.0 (5.8) | 0.25 |
| **Dialysis vintage (months), median (IQR)** | 29.1 (13.9-52.9) | 35.0 (17.9-58.2) | 0.021 |
| **Prior kidney transplant (N (%))** |  |  | <0.001 |
| No | 261 (84.2%) | 344 (94.8%) |  |
| Yes | 49 (15.8%) | 19 (5.2%) |  |
| **Multi-organ transplantation (N (%))** |  |  | 0.10 |
| Liver + kidney | 6 (1.9%) | 9 (2.5%) |  |
| Pancreas + kidney | 4 (1.3%) | 14 (3.9%) |  |
| Heart + kidney | 4 (1.3%) | 7 (1.9%) |  |
| None | 296 (95.5%) | 330 (90.9%) |  |
| Liver +Heart+Kidney | 0 (0.0%) | 3 (0.8%) |  |
| **CMV risk categories (N (%))** |  |  | 0.31 |
| Low | 80 (25.8%) | 88 (24.2%) |  |
| Intermediate | 139 (44.8%) | 155 (42.7%) |  |
| High | 79 (25.5%) | 112 (30.9%) |  |
| Unknow | 12 (3.9%) | 8 (2.2%) |  |
| **Was the donor organ pumped? (N (%))** |  |  | 0.092 |
| No | 91 (31.7%) | 89 (25.6%) |  |
| Yes | 196 (68.3%) | 258 (74.4%) |  |
| **En block kidney (N (%))** | 1 (2%) | 13 (20%) | 0.003 |
| **Dual kidney (N (%))** | 5 (10%) | 10 (15%) | 0.40 |
| **Transplantation characteristics** |  |  |  |
| **Cold ischemic time (hours), median (IQR)** | 12.6 (4.4-21.0) | 13.7 (6.3-19.8) | 0.74 |
| **cPRA (%), median (IQR)** | 0 (0-27) | 0 (0-0) | <0.001 |
| **Donors’ characteristics** |  |  |  |
| **Age (years), mean (SD)** | 38.5 (15.4) | 36.4 (16.0) | 0.082 |
| **Gender (N (%))** |  |  | 0.12 |
| male | 165 (53.2%) | 215 (59.2%) |  |
| female | 145 (46.8%) | 148 (40.8%) |  |
| **Race/ethnicity** |  |  | 0.95 |
| White | 250 (80.6%) | 295 (81.3%) |  |
| African American | 9 (2.9%) | 12 (3.3%) |  |
| Asian | 5 (1.6%) | 8 (2.2%) |  |
| Other | 5 (1.6%) | 5 (1.4%) |  |
| Unknown | 41 (13.2%) | 43 (11.8%) |  |
| **Donor type (N (%))** |  |  | 0.035 |
| Living | 107 (34.5%) | 98 (27.0%) |  |
| Deceased | 203 (65.5%) | 265 (73.0%) |  |
| **Donor DCD (N (%))** |  |  | 0.24 |
| No | 110 (54.2%) | 158 (59.6%) |  |
| Yes | 93 (45.8%) | 107 (40.4%) |  |
| **KDPI, median (IQR)** | 28 (13-53) | 28 (12-55) | 0.80 |
| **Donor cause of death (N (%))** |  |  | 0.91 |
| Anoxia | 100 (49.3%) | 130 (49.1%) |  |
| Cerebrovascular/stroke | 31 (15.3%) | 35 (13.2%) |  |
| Head trauma | 64 (31.5%) | 91 (34.3%) |  |
| Central nervous system tumor | 1 (0.5%) | 2 (0.8%) |  |
| Other | 7 (3.4%) | 7 (2.6%) |  |
| **Donor terminal creatinine (mg/dl), median (IQR)** | 0.80 (0.64-1.04) | 0.83 (0.66-1.02) | 0.46 |
| **Donors diabetes (N (%))** |  |  | 0.82 |
| No | 294 (95.1%) | 341 (95.5%) |  |
| Yes | 15 (4.9%) | 16 (4.5%) |  |
| **Donors hypertension (N (%))** |  |  | 0.39 |
| No | 265 (86.3%) | 298 (83.9%) |  |
| Yes | 42 (13.7%) | 57 (16.1%) |  |
| **Donor malignancy (N (%))** |  |  | 0.54 |
| No | 303 (97.7%) | 352 (97.0%) |  |
| Yes |  |  |  |
| **Immunological characteristics** | 7 (2.3%) | 11 (3.0%) |  |
| **Number of HLA mismatches (HLA A,B and DR) (N (%))** |  |  | <0.001 |
| 0 | 20 (6.5%) | 0 (0.0%) |  |
| 1 | 17 (5.5%) | 0 (0.0%) |  |
| 2 | 36 (11.6%) | 4 (1.1%) |  |
| 3 | 88 (28.4%) | 33 (9.1%) |  |
| 4 | 83 (26.8%) | 78 (21.5%) |  |
| 5 | 45 (14.5%) | 166 (45.7%) |  |
| 6 | 21 (6.8%) | 82 (22.6%) |  |
| **HLA mismatches A (N (%))** |  |  | <0.001 |
| 0 | 19 (22%) | 3 (4%) |  |
| 1 | 44 (51%) | 31 (40%) |  |
| 2 | 23 (27%) | 43 (56%) |  |
| **HLA mismatches B (N (%))** |  |  | 0.005 |
| 0 | 10 (12%) | 3 (4%) |  |
| 1 | 31 (36%) | 15 (19%) |  |
| 2 | 45 (52%) | 59 (77%) |  |
| **HLA mismatches DR (N (%))** |  |  | <0.001 |
| 0 | 43 (26.9%) | 5 (2.9%) |  |
| 1 | 90 (56.2%) | 79 (45.1%) |  |
| 2 | 27 (16.9%) | 91 (52.0%) |  |
| **HLA mismatches DQ (N (%))** |  |  | <0.001 |
| 0 | 33 (38%) | 1 (1%) |  |
| 1 | 44 (51%) | 36 (47%) |  |
| 2 | 9 (10%) | 39 (51%) |  |
| **Current flow B cell (N (%))** |  |  | 0.77 |
| < 2 SD compatible | 152 (93.3%) | 167 (91.3%) |  |
| 2-3 SD compatible | 7 (4.3%) | 8 (4.4%) |  |
| weak positive | 1 (0.6%) | 3 (1.6%) |  |
| positive | 3 (1.8%) | 5 (2.7%) |  |
| **Current flow T cell (N (%))** |  |  | 0.82 |
| < 2 SD compatible | 151 (92.6%) | 171 (93.4%) |  |
| 2-3 SD compatible | 7 (4.3%) | 5 (2.7%) |  |
| weak positive | 2 (1.2%) | 2 (1.1%) |  |
| positive | 3 (1.8%) | 5 (2.7%) |  |
| **Induction treatment (N (%))** |  |  |  |
| No Induction | 0 (0%) | 0 (0%) | 1.000 |
| Basiliximab | 41 (13.2%) | 36 (9.9%) | 0.18 |
| Thymoglobulin | 250 (80.6%) | 313 (86.2%) | 0.051 |
| Steroid | 310 (100.0%) | 362 (99.7%) | 0.36 |
| Alemtuzumab | 10 (3.2%) | 11 (3.0%) | 0.88 |
| **Outcomes** |  |  |  |
| **Proportion of Antibody Mediated Rejection of the subset of patient underwent biopsy (N (%))** |  |  | 0.008 |
| No | 90 (83.3%) | 94 (68.6%) |  |
| Yes | 18 (16.7%) | 43 (31.4%) |  |
| **Proportion of rejection of the subset of patient underwent biopsy (N (%))** |  |  | 0.023 |
| No | 73 (67.6%) | 73 (53.3%) |  |
| Yes | 35 (32.4%) | 64 (46.7%) |  |
| **Proportion of T-cell Mediated Rejection of the subset of patient underwent biopsy (N (%))** |  |  | 0.094 |
| No | 78 (72.2%) | 85 (62.0%) |  |
| Yes | 30 (27.8%) | 52 (38.0%) |  |
| **Post-Transplant Donor Specific Antibodies (N (%))** |  |  | <0.001 |
| No | 263 (85.1%) | 252 (69.4%) |  |
| Yes | 46 (14.9%) | 111 (30.6%) |  |
| **Dd-cf-DNA elevation (N (%))** |  |  | 0.052 |
| No | 195 (82.6%) | 214 (75.6%) |  |
| Yes | 41 (17.4%) | 69 (24.4%) |  |
| **Primary outcome (N (%))** |  |  | <0.001 |
| No | 225 (72.6%) | 198 (54.5%) |  |
| Yes | 85 (27.4%) | 165 (45.5%) |  |
| **Delayed Graft Function (N (%))** |  |  | 0.53 |
| No | 281 (90.6%) | 334 (92.0%) |  |
| Yes | 29 (9.4%) | 29 (8.0%) |  |
| **Death (N (%))** |  |  | 0.53 |
| No | 294 (94.8%) | 348 (95.9%) |  |
| Yes | 16 (5.2%) | 15 (4.1%) |  |
| **Graft Loss (N (%))** |  |  | 0.57 |
| No | 302 (97.4%) | 356 (98.1%) |  |
| Yes | 8 (2.6%) | 7 (1.9%) |  |

Values are expressed as mean (standard deviation), median (interquartile range), or number (%). Continuous variables were compared via t-tests or Mann-Whitney U tests. Categorical variables were compared via Chi-square tests.

**Table S7:** Performance characteristics table of PIRCHE-T2 for DSA

| Cutpoint | Sensitivity | Specificity | Overall % correctly classified | LR+ | LR- |
| --- | --- | --- | --- | --- | --- |
| ( >= 0 ) | 100.00% | 0.00% | 23.36% | 1 |  |
| ( >= 1 ) | 99.36% | 2.72% | 25.30% | 1.0214 | 0.2343 |
| ( >= 5 ) | 99.36% | 2.91% | 25.45% | 1.0234 | 0.2187 |
| ( >= 7 ) | 99.36% | 3.11% | 25.60% | 1.0255 | 0.205 |
| ( >= 8 ) | 98.73% | 3.11% | 25.45% | 1.0189 | 0.41 |
| ( >= 9 ) | 98.73% | 3.69% | 25.89% | 1.0251 | 0.3453 |
| ( >= 10 ) | 98.09% | 3.69% | 25.74% | 1.0185 | 0.5179 |
| ( >= 11 ) | 97.45% | 3.88% | 25.74% | 1.0139 | 0.6561 |
| ( >= 12 ) | 97.45% | 4.47% | 26.19% | 1.0201 | 0.5705 |
| ( >= 13 ) | 97.45% | 4.85% | 26.49% | 1.0242 | 0.5248 |
| ( >= 14 ) | 97.45% | 5.05% | 26.64% | 1.0263 | 0.5047 |
| ( >= 15 ) | 97.45% | 5.24% | 26.79% | 1.0284 | 0.486 |
| ( >= 16 ) | 97.45% | 5.83% | 27.23% | 1.0348 | 0.4374 |
| ( >= 17 ) | 97.45% | 6.02% | 27.38% | 1.0369 | 0.4233 |
| ( >= 18 ) | 97.45% | 6.99% | 28.12% | 1.0478 | 0.3645 |
| ( >= 18.26 ) | 97.45% | 7.18% | 28.27% | 1.05 | 0.3546 |
| ( >= 19 ) | 97.45% | 7.38% | 28.42% | 1.0522 | 0.3453 |
| ( >= 19.04 ) | 96.82% | 7.96% | 28.72% | 1.0519 | 0.4 |
| ( >= 20 ) | 96.82% | 8.16% | 28.87% | 1.0541 | 0.3905 |
| ( >= 21 ) | 96.18% | 8.93% | 29.32% | 1.0561 | 0.4279 |
| ( >= 22 ) | 95.54% | 9.71% | 29.76% | 1.0581 | 0.4592 |
| ( >= 23 ) | 95.54% | 10.49% | 30.36% | 1.0673 | 0.4252 |
| ( >= 24 ) | 95.54% | 11.46% | 31.10% | 1.079 | 0.3892 |
| ( >= 24.96 ) | 95.54% | 11.84% | 31.40% | 1.0838 | 0.3764 |
| ( >= 25 ) | 94.90% | 11.84% | 31.25% | 1.0766 | 0.4302 |
| ( >= 26 ) | 94.27% | 12.04% | 31.25% | 1.0717 | 0.4762 |
| ( >= 27 ) | 94.27% | 12.62% | 31.70% | 1.0788 | 0.4542 |
| ( >= 28 ) | 93.63% | 13.20% | 31.99% | 1.0787 | 0.4824 |
| ( >= 29 ) | 92.99% | 13.98% | 32.44% | 1.0811 | 0.5012 |
| ( >= 30 ) | 92.36% | 14.37% | 32.59% | 1.0785 | 0.5319 |
| ( >= 31 ) | 91.08% | 14.95% | 32.74% | 1.071 | 0.5964 |
| ( >= 31.71 ) | 90.45% | 16.31% | 33.63% | 1.0807 | 0.5858 |
| ( >= 32 ) | 90.45% | 16.50% | 33.78% | 1.0832 | 0.5789 |
| ( >= 33 ) | 89.17% | 17.86% | 34.52% | 1.0857 | 0.6061 |
| ( >= 33.22 ) | 87.90% | 18.83% | 34.97% | 1.083 | 0.6425 |
| ( >= 34 ) | 87.90% | 19.03% | 35.12% | 1.0856 | 0.636 |
| ( >= 35 ) | 86.62% | 20.00% | 35.57% | 1.0828 | 0.6688 |
| ( >= 35.65 ) | 84.08% | 20.39% | 35.27% | 1.0561 | 0.781 |
| ( >= 36 ) | 84.08% | 20.58% | 35.42% | 1.0587 | 0.7736 |
| ( >= 36.47 ) | 84.08% | 22.33% | 36.76% | 1.0825 | 0.7131 |
| ( >= 37 ) | 84.08% | 22.52% | 36.90% | 1.0852 | 0.707 |
| ( >= 38 ) | 82.80% | 24.47% | 38.10% | 1.0962 | 0.7029 |
| ( >= 38.22 ) | 82.17% | 25.05% | 38.39% | 1.0963 | 0.712 |
| ( >= 38.54 ) | 82.17% | 25.24% | 38.54% | 1.0991 | 0.7065 |
| ( >= 39 ) | 81.53% | 25.24% | 38.39% | 1.0906 | 0.7317 |
| ( >= 39.24 ) | 80.89% | 26.99% | 39.58% | 1.108 | 0.708 |
| ( >= 40 ) | 80.89% | 27.18% | 39.73% | 1.1109 | 0.7029 |
| ( >= 40.25 ) | 80.25% | 27.96% | 40.18% | 1.114 | 0.7062 |
| ( >= 41 ) | 80.25% | 28.16% | 40.33% | 1.1171 | 0.7013 |
| ( >= 41.11 ) | 77.71% | 28.54% | 40.03% | 1.0875 | 0.781 |
| ( >= 42 ) | 77.71% | 28.74% | 40.18% | 1.0904 | 0.7757 |
| ( >= 43 ) | 77.71% | 30.29% | 41.37% | 1.1147 | 0.736 |
| ( >= 44 ) | 76.43% | 31.26% | 41.82% | 1.112 | 0.7538 |
| ( >= 45 ) | 75.16% | 31.46% | 41.67% | 1.0965 | 0.7897 |
| ( >= 45.35 ) | 73.89% | 32.82% | 42.41% | 1.0997 | 0.7958 |
| ( >= 45.78 ) | 73.89% | 33.01% | 42.56% | 1.1029 | 0.7911 |
| ( >= 46 ) | 73.25% | 33.01% | 42.41% | 1.0934 | 0.8104 |
| ( >= 47 ) | 71.34% | 34.17% | 42.86% | 1.0837 | 0.8387 |
| ( >= 48 ) | 71.34% | 35.15% | 43.60% | 1.1 | 0.8155 |
| ( >= 48.19 ) | 70.70% | 35.92% | 44.05% | 1.1034 | 0.8156 |
| ( >= 48.23 ) | 70.70% | 36.12% | 44.20% | 1.1067 | 0.8112 |
| ( >= 49 ) | 70.70% | 36.31% | 44.35% | 1.1101 | 0.8069 |
| ( >= 49.32 ) | 69.43% | 38.25% | 45.54% | 1.1244 | 0.7992 |
| ( >= 50 ) | 69.43% | 38.45% | 45.68% | 1.1279 | 0.7952 |
| ( >= 50.08 ) | 66.88% | 40.78% | 46.88% | 1.1293 | 0.8123 |
| ( >= 51 ) | 66.88% | 40.97% | 47.02% | 1.133 | 0.8084 |
| ( >= 51.43 ) | 66.24% | 42.72% | 48.21% | 1.1564 | 0.7902 |
| ( >= 52 ) | 66.24% | 42.91% | 48.36% | 1.1604 | 0.7867 |
| ( >= 53 ) | 65.61% | 44.66% | 49.55% | 1.1855 | 0.7701 |
| ( >= 54 ) | 63.69% | 45.63% | 49.85% | 1.1715 | 0.7956 |
| ( >= 54.3 ) | 63.69% | 46.80% | 50.74% | 1.1972 | 0.7758 |
| ( >= 55 ) | 63.06% | 46.80% | 50.60% | 1.1852 | 0.7894 |
| ( >= 55.59 ) | 61.15% | 48.54% | 51.49% | 1.1883 | 0.8004 |
| ( >= 55.89 ) | 60.51% | 48.54% | 51.34% | 1.1759 | 0.8135 |
| ( >= 56 ) | 60.51% | 48.74% | 51.49% | 1.1804 | 0.8103 |
| ( >= 57 ) | 59.87% | 50.29% | 52.53% | 1.2045 | 0.7979 |
| ( >= 57.04 ) | 55.41% | 51.84% | 52.68% | 1.1507 | 0.86 |
| ( >= 57.83 ) | 54.78% | 51.84% | 52.53% | 1.1375 | 0.8723 |
| ( >= 58 ) | 54.14% | 51.84% | 52.38% | 1.1243 | 0.8846 |
| ( >= 58.94 ) | 52.23% | 53.01% | 52.83% | 1.1115 | 0.9012 |
| ( >= 59 ) | 52.23% | 53.20% | 52.98% | 1.1161 | 0.8979 |
| ( >= 59.83 ) | 50.96% | 54.76% | 53.87% | 1.1263 | 0.8957 |
| ( >= 60 ) | 50.96% | 54.95% | 54.02% | 1.1311 | 0.8925 |
| ( >= 61 ) | 49.04% | 56.50% | 54.76% | 1.1276 | 0.9018 |
| ( >= 61.79 ) | 48.41% | 59.03% | 56.55% | 1.1815 | 0.874 |
| ( >= 61.96 ) | 48.41% | 59.22% | 56.70% | 1.1871 | 0.8711 |
| ( >= 62 ) | 48.41% | 59.42% | 56.85% | 1.1928 | 0.8683 |
| ( >= 63 ) | 47.13% | 60.78% | 57.59% | 1.2017 | 0.8698 |
| ( >= 64 ) | 46.50% | 61.55% | 58.04% | 1.2094 | 0.8692 |
| ( >= 64.81 ) | 46.50% | 62.14% | 58.48% | 1.228 | 0.8611 |
| ( >= 65 ) | 46.50% | 62.33% | 58.63% | 1.2343 | 0.8584 |
| ( >= 66 ) | 45.86% | 63.88% | 59.67% | 1.2698 | 0.8475 |
| ( >= 66.94 ) | 44.59% | 65.05% | 60.27% | 1.2757 | 0.8519 |
| ( >= 67 ) | 44.59% | 65.24% | 60.42% | 1.2828 | 0.8494 |
| ( >= 67.07 ) | 43.31% | 66.21% | 60.86% | 1.2819 | 0.8561 |
| ( >= 68 ) | 43.31% | 66.41% | 61.01% | 1.2893 | 0.8536 |
| ( >= 68.08 ) | 41.40% | 68.35% | 62.05% | 1.3081 | 0.8573 |
| ( >= 68.79 ) | 40.76% | 68.35% | 61.90% | 1.288 | 0.8667 |
| ( >= 69 ) | 40.76% | 68.54% | 62.05% | 1.2959 | 0.8642 |
| ( >= 69.96 ) | 40.76% | 69.71% | 62.95% | 1.3457 | 0.8498 |
| ( >= 70 ) | 40.76% | 69.90% | 63.10% | 1.3544 | 0.8474 |
| ( >= 70.5 ) | 39.49% | 71.26% | 63.84% | 1.3742 | 0.8491 |
| ( >= 71 ) | 39.49% | 71.46% | 63.99% | 1.3835 | 0.8468 |
| ( >= 71.3 ) | 39.49% | 72.43% | 64.73% | 1.4322 | 0.8355 |
| ( >= 71.68 ) | 39.49% | 72.62% | 64.88% | 1.4424 | 0.8332 |
| ( >= 72 ) | 38.85% | 72.62% | 64.73% | 1.4191 | 0.842 |
| ( >= 72.47 ) | 38.85% | 73.20% | 65.18% | 1.45 | 0.8353 |
| ( >= 73 ) | 38.85% | 73.40% | 65.33% | 1.4606 | 0.8331 |
| ( >= 73.8 ) | 35.67% | 74.37% | 65.33% | 1.3916 | 0.865 |
| ( >= 73.94 ) | 35.67% | 74.56% | 65.48% | 1.4022 | 0.8628 |
| ( >= 74 ) | 35.67% | 74.76% | 65.62% | 1.413 | 0.8605 |
| ( >= 75 ) | 34.39% | 75.73% | 66.07% | 1.4171 | 0.8663 |
| ( >= 76 ) | 33.12% | 76.31% | 66.22% | 1.3981 | 0.8764 |
| ( >= 77 ) | 29.94% | 77.09% | 66.07% | 1.3065 | 0.9089 |
| ( >= 78 ) | 28.66% | 78.64% | 66.96% | 1.3419 | 0.9071 |
| ( >= 78.02 ) | 26.75% | 79.42% | 67.11% | 1.2997 | 0.9223 |
| ( >= 79 ) | 26.75% | 79.61% | 67.26% | 1.3121 | 0.9201 |
| ( >= 80 ) | 26.11% | 80.58% | 67.86% | 1.3449 | 0.9169 |
| ( >= 81 ) | 24.20% | 81.75% | 68.30% | 1.3261 | 0.9272 |
| ( >= 81.83 ) | 21.02% | 83.30% | 68.75% | 1.2587 | 0.9481 |
| ( >= 82 ) | 21.02% | 83.50% | 68.90% | 1.2735 | 0.9459 |
| ( >= 83 ) | 19.75% | 84.47% | 69.35% | 1.2711 | 0.9501 |
| ( >= 83.34 ) | 19.11% | 85.05% | 69.64% | 1.278 | 0.9511 |
| ( >= 83.69 ) | 19.11% | 85.24% | 69.79% | 1.2948 | 0.949 |
| ( >= 84 ) | 19.11% | 85.44% | 69.94% | 1.3121 | 0.9468 |
| ( >= 85 ) | 17.83% | 85.83% | 69.94% | 1.2582 | 0.9574 |
| ( >= 85.33 ) | 17.20% | 86.99% | 70.68% | 1.3219 | 0.9519 |
| ( >= 86 ) | 16.56% | 86.99% | 70.54% | 1.2729 | 0.9592 |
| ( >= 86.17 ) | 15.92% | 87.38% | 70.68% | 1.2616 | 0.9622 |
| ( >= 87 ) | 15.29% | 87.38% | 70.54% | 1.2112 | 0.9695 |
| ( >= 88 ) | 15.29% | 88.16% | 71.13% | 1.2906 | 0.961 |
| ( >= 89 ) | 15.29% | 88.54% | 71.43% | 1.3343 | 0.9567 |
| ( >= 89.31 ) | 13.38% | 89.51% | 71.73% | 1.2757 | 0.9677 |
| ( >= 90 ) | 13.38% | 89.71% | 71.88% | 1.2997 | 0.9656 |
| ( >= 91 ) | 12.10% | 90.68% | 72.32% | 1.2984 | 0.9693 |
| ( >= 91.07 ) | 11.46% | 91.26% | 72.62% | 1.3121 | 0.9701 |
| ( >= 92 ) | 11.46% | 91.46% | 72.77% | 1.3419 | 0.9681 |
| ( >= 93 ) | 11.46% | 91.84% | 73.07% | 1.4058 | 0.964 |
| ( >= 94 ) | 11.46% | 92.82% | 73.81% | 1.5958 | 0.9539 |
| ( >= 95 ) | 11.46% | 93.01% | 73.96% | 1.6401 | 0.9519 |
| ( >= 95.94 ) | 10.19% | 93.40% | 73.96% | 1.5436 | 0.9616 |
| ( >= 96 ) | 9.55% | 93.40% | 73.81% | 1.4472 | 0.9684 |
| ( >= 97 ) | 9.55% | 93.59% | 73.96% | 1.491 | 0.9664 |
| ( >= 98 ) | 8.28% | 94.17% | 74.11% | 1.4214 | 0.9739 |
| ( >= 99 ) | 7.64% | 95.15% | 74.70% | 1.5745 | 0.9707 |
| ( >= 100 ) | 7.64% | 95.92% | 75.30% | 1.8744 | 0.9628 |
| ( >= 101 ) | 7.64% | 96.12% | 75.45% | 1.9682 | 0.9609 |
| ( >= 101.13 ) | 7.64% | 96.50% | 75.74% | 2.1868 | 0.957 |
| ( >= 102 ) | 7.64% | 96.70% | 75.89% | 2.3155 | 0.9551 |
| ( >= 103 ) | 6.37% | 97.09% | 75.89% | 2.1868 | 0.9644 |
| ( >= 105 ) | 5.73% | 97.09% | 75.74% | 1.9682 | 0.971 |
| ( >= 106 ) | 5.73% | 97.28% | 75.89% | 2.1087 | 0.969 |
| ( >= 107 ) | 5.10% | 97.48% | 75.89% | 2.0186 | 0.9736 |
| ( >= 108 ) | 4.46% | 97.48% | 75.74% | 1.7663 | 0.9802 |
| ( >= 109 ) | 4.46% | 97.67% | 75.89% | 1.9135 | 0.9782 |
| ( >= 110 ) | 3.18% | 97.67% | 75.60% | 1.3668 | 0.9912 |
| ( >= 111 ) | 3.18% | 98.06% | 75.89% | 1.6401 | 0.9873 |
| ( >= 112 ) | 2.55% | 98.45% | 76.04% | 1.6401 | 0.9899 |
| ( >= 113 ) | 1.91% | 98.64% | 76.04% | 1.4058 | 0.9944 |
| ( >= 114 ) | 1.91% | 99.03% | 76.34% | 1.9681 | 0.9905 |
| ( >= 116 ) | 1.91% | 99.22% | 76.49% | 2.4602 | 0.9886 |
| ( >= 118 ) | 1.91% | 99.42% | 76.64% | 3.2803 | 0.9866 |
| ( >= 120.6 ) | 1.27% | 99.42% | 76.49% | 2.1869 | 0.993 |
| ( >= 121 ) | 1.27% | 99.61% | 76.64% | 3.2802 | 0.9911 |
| ( >= 128 ) | 1.27% | 99.81% | 76.79% | 6.5605 | 0.9892 |
| ( >= 132 ) | 0.64% | 99.81% | 76.64% | 3.2802 | 0.9956 |
| ( >= 137 ) | 0.64% | 100.00% | 76.79% |  | 0.9936 |
| ( > 137 ) | 0.00% | 100.00% | 76.64% |  | 1 |

LR: Likelihood ratio

**Table S8:** Performance characteristics table of PIRCHE-B for DSA

| Cutpoint | Sensitivity | Specificity | Overall % correctly classified | LR+ | LR- |
| --- | --- | --- | --- | --- | --- |
| ( >= 0 ) | 100.00% | 0.00% | 23.36% | 1 |  |
| ( >= 1 ) | 99.36% | 3.11% | 25.60% | 1.0255 | 0.205 |
| ( >= 2 ) | 96.82% | 4.08% | 25.74% | 1.0093 | 0.781 |
| ( >= 3 ) | 96.18% | 5.63% | 26.79% | 1.0192 | 0.6787 |
| ( >= 4 ) | 94.90% | 8.35% | 28.57% | 1.0355 | 0.6103 |
| ( >= 5 ) | 93.63% | 10.29% | 29.76% | 1.0437 | 0.6189 |
| ( >= 6 ) | 91.72% | 13.40% | 31.70% | 1.0591 | 0.618 |
| ( >= 6.69 ) | 91.72% | 18.64% | 35.71% | 1.1273 | 0.4442 |
| ( >= 7 ) | 91.72% | 18.83% | 35.86% | 1.13 | 0.4396 |
| ( >= 8 ) | 90.45% | 24.27% | 39.73% | 1.1943 | 0.3936 |
| ( >= 9 ) | 87.26% | 28.54% | 42.26% | 1.2212 | 0.4463 |
| ( >= 9.02 ) | 82.80% | 35.34% | 46.43% | 1.2806 | 0.4866 |
| ( >= 9.51 ) | 82.80% | 35.53% | 46.58% | 1.2844 | 0.484 |
| ( >= 10 ) | 82.80% | 35.73% | 46.73% | 1.2883 | 0.4813 |
| ( >= 10.71 ) | 78.34% | 40.39% | 49.26% | 1.3142 | 0.5362 |
| ( >= 11 ) | 78.34% | 40.58% | 49.40% | 1.3185 | 0.5336 |
| ( >= 12 ) | 74.52% | 45.63% | 52.38% | 1.3707 | 0.5583 |
| ( >= 13 ) | 70.70% | 51.07% | 55.65% | 1.4449 | 0.5737 |
| ( >= 13.17 ) | 64.33% | 56.31% | 58.18% | 1.4725 | 0.6334 |
| ( >= 14 ) | 63.69% | 56.31% | 58.04% | 1.4579 | 0.6447 |
| ( >= 15 ) | 55.41% | 60.39% | 59.23% | 1.3989 | 0.7383 |
| ( >= 16 ) | 50.96% | 66.02% | 62.50% | 1.4995 | 0.7429 |
| ( >= 17 ) | 44.59% | 70.29% | 64.29% | 1.5008 | 0.7883 |
| ( >= 17.24 ) | 36.94% | 73.98% | 65.33% | 1.4198 | 0.8523 |
| ( >= 18 ) | 36.31% | 73.98% | 65.18% | 1.3953 | 0.861 |
| ( >= 19 ) | 35.67% | 79.03% | 68.90% | 1.7009 | 0.814 |
| ( >= 20 ) | 31.85% | 81.55% | 69.94% | 1.7264 | 0.8357 |
| ( >= 21 ) | 27.39% | 84.85% | 71.43% | 1.8083 | 0.8557 |
| ( >= 21.07 ) | 19.75% | 88.74% | 72.62% | 1.7532 | 0.9044 |
| ( >= 22 ) | 19.11% | 88.74% | 72.47% | 1.6967 | 0.9116 |
| ( >= 23 ) | 15.29% | 90.49% | 72.92% | 1.6067 | 0.9362 |
| ( >= 24 ) | 11.46% | 92.23% | 73.36% | 1.4761 | 0.9599 |
| ( >= 25 ) | 9.55% | 94.17% | 74.40% | 1.6401 | 0.9604 |
| ( >= 26 ) | 7.64% | 96.12% | 75.45% | 1.9682 | 0.9609 |
| ( >= 27 ) | 6.37% | 97.86% | 76.49% | 2.9821 | 0.9567 |
| ( >= 28 ) | 6.37% | 98.64% | 77.08% | 4.6861 | 0.9492 |
| ( >= 30 ) | 4.46% | 99.22% | 77.08% | 5.7405 | 0.9629 |
| ( >= 31 ) | 3.18% | 99.22% | 76.79% | 4.1003 | 0.9757 |
| ( >= 32 ) | 2.55% | 99.61% | 76.93% | 6.5605 | 0.9783 |
| ( >= 33 ) | 1.91% | 99.61% | 76.79% | 4.9204 | 0.9847 |
| ( >= 34 ) | 1.27% | 99.81% | 76.79% | 6.5605 | 0.9892 |
| ( >= 50 ) | 0.64% | 99.81% | 76.64% | 3.2802 | 0.9956 |
| ( >= 54 ) | 0.00% | 99.81% | 76.49% | 0 | 1.0019 |
| ( > 54 ) | 0.00% | 100.00% | 76.64% |  | 1 |

LR: Likelihood ratio

**Table S9:** Patient characteristics of the rejection cohort based on PIRCHE-T2 score

|  | **Low Risk PIRCHE-T2 Score Group** | **High Risk PIRCHE-T2 Score Group** | **p-value** |
| --- | --- | --- | --- |
| **N** | 142 (58%) | 104 (42%) |  |
| **Recipients’ characteristics** |  |  |  |
| **Age (years), mean (SD)** | 47.8 (15.5) | 50.4 (14.0) | 0.17 |
| **Gender (N (%))** |  |  | 0.69 |
| male | 88 (62.0%) | 67 (64.4%) |  |
| female | 54 (38.0%) | 37 (35.6%) |  |
| **Race/ethnicity (N (%))** |  |  | 0.13 |
| White | 97 (68.3%) | 62 (59.6%) |  |
| Hispanic | 28 (19.7%) | 19 (18.3%) |  |
| African American | 1 (0.7%) | 4 (3.8%) |  |
| Asian | 3 (2.1%) | 7 (6.7%) |  |
| Other | 13 (9.2%) | 12 (11.5%) |  |
| Unknown | 0 (0%) | 0 (0%) |  |
| **Body Mass Index (kg/m2), mean (SD)** | 28.8 (6.1) | 29.3 (5.9) | 0.52 |
| **Dialysis vintage (months), median (IQR)** | 32.9 (14.4-55.7) | 41.0 (24.5-65.9) | 0.036 |
| **Prior kidney transplant (N (%))** |  |  | 0.012 |
| No | 115 (81.0%) | 96 (92.3%) |  |
| Yes | 27 (19.0%) | 8 (7.7%) |  |
| **Multi-organ transplantation (N (%))** |  |  | 0.28 |
| Liver + kidney | 2 (1.4%) | 1 (1.0%) |  |
| Pancreas + kidney | 2 (1.4%) | 1 (1.0%) |  |
| Heart + kidney | 1 (0.7%) | 2 (1.9%) |  |
| None | 137 (96.5%) | 97 (93.3%) |  |
| Liver +Heart+Kidney | 0 (0.0%) | 3 (2.9%) |  |
| **CMV risk categories (N (%))** |  |  | 0.41 |
| Low | 34 (23.9%) | 24 (23.1%) |  |
| Intermediate | 57 (40.1%) | 47 (45.2%) |  |
| High | 50 (35.2%) | 30 (28.8%) |  |
| Unknow | 1 (0.7%) | 3 (2.9%) |  |
| **Was the donor organ pumped? (N (%))** |  |  | 0.11 |
| No | 38 (28.1%) | 19 (19.2%) |  |
| Yes | 97 (71.9%) | 80 (80.8%) |  |
| **En block kidney (N (%))** | 3 (13%) | 4 (22%) | 0.44 |
| **Dual kidney (N (%))** | 1 (4%) | 4 (22%) | 0.083 |
| **Transplantation characteristics** |  |  |  |
| **Cold ischemic time (hours), median (IQR)** | 14.6 (4.7-22.2) | 15.9 (8.4-20.9) | 0.74 |
| **cPRA (%), median (IQR)** | 0 (0-65) | 0 (0-0) | <0.001 |
| **Donors’ characteristics** |  |  |  |
| **Age (years), mean (SD)** | 39.1 (16.2) | 37.5 (16.4) | 0.43 |
| **Gender (N (%))** |  |  | 0.38 |
| male | 78 (54.9%) | 63 (60.6%) |  |
| female | 64 (45.1%) | 41 (39.4%) |  |
| **Race/ethnicity** |  |  | 0.22 |
| White | 108 (76.1%) | 90 (86.5%) |  |
| African American | 6 (4.2%) | 3 (2.9%) |  |
| Asian | 3 (2.1%) | 3 (2.9%) |  |
| Other | 4 (2.8%) | 1 (1.0%) |  |
| Unknown | 21 (14.8%) | 7 (6.7%) |  |
| **Donor type (N (%))** |  |  | 0.21 |
| Living | 40 (28.2%) | 22 (21.2%) |  |
| Deceased | 102 (71.8%) | 82 (78.8%) |  |
| **Donor DCD (N (%))** |  |  | 0.034 |
| No | 62 (60.8%) | 37 (45.1%) |  |
| Yes | 40 (39.2%) | 45 (54.9%) |  |
| **KDPI, median (IQR)** | 33 (15-53) | 34 (20-63) | 0.24 |
| **Donor cause of death (N (%))** |  |  | 0.79 |
| Anoxia | 51 (50.0%) | 43 (52.4%) |  |
| Cerebrovascular/stroke | 13 (12.7%) | 14 (17.1%) |  |
| Head trauma | 34 (33.3%) | 23 (28.0%) |  |
| Central nervous system tumor | 1 (1.0%) | 1 (1.2%) |  |
| Other | 3 (2.9%) | 1 (1.2%) |  |
| **Donor terminal creatinine (mg/dl), median (IQR)** | 0.82 (0.64-1.05) | 0.81 (0.63-1.03) | 0.97 |
| **Donors diabetes (N (%))** |  |  | 0.67 |
| No | 129 (92.8%) | 97 (94.2%) |  |
| Yes | 10 (7.2%) | 6 (5.8%) |  |
| **Donors hypertension (N (%))** |  |  | 0.23 |
| No | 116 (83.5%) | 78 (77.2%) |  |
| Yes | 23 (16.5%) | 23 (22.8%) |  |
| **Donor malignancy (N (%))** |  |  | 0.65 |
| No | 138 (97.2%) | 102 (98.1%) |  |
| Yes | 4 (2.8%) | 2 (1.9%) |  |
| **Immunological characteristics** |  |  |  |
| **Number of HLA mismatches (HLA A,B and DR) (N (%))** |  |  | <0.001 |
| 0 | 9 (6.3%) | 0 (0.0%) |  |
| 1 | 6 (4.2%) | 0 (0.0%) |  |
| 2 | 12 (8.5%) | 1 (1.0%) |  |
| 3 | 38 (26.8%) | 2 (1.9%) |  |
| 4 | 30 (21.1%) | 27 (26.0%) |  |
| 5 | 32 (22.5%) | 45 (43.3%) |  |
| 6 | 15 (10.6%) | 29 (27.9%) |  |
| **HLA mismatches A (N (%))** |  |  | <0.001 |
| 0 | 3 (10%) | 1 (6%) |  |
| 1 | 20 (67%) | 1 (6%) |  |
| 2 | 7 (23%) | 15 (88%) |  |
| **HLA mismatches B (N (%))** |  |  | 0.11 |
| 0 | 3 (10%) | 0 (0%) |  |
| 1 | 9 (30%) | 2 (12%) |  |
| 2 | 18 (60%) | 15 (88%) |  |
| **HLA mismatches DR (N (%))** |  |  | 0.018 |
| 0 | 13 (21%) | 4 (9%) |  |
| 1 | 29 (47%) | 15 (33%) |  |
| 2 | 20 (32%) | 27 (59%) |  |
| **HLA mismatches DQ (N (%))** |  |  | 0.51 |
| 0 | 10 (33%) | 3 (18%) |  |
| 1 | 13 (43%) | 9 (53%) |  |
| 2 | 7 (23%) | 5 (29%) |  |
| **Current flow B cell (N (%))** |  |  | 0.28 |
| < 2 SD compatible | 58 (91%) | 47 (92%) |  |
| 2-3 SD compatible | 4 (6%) | 1 (2%) |  |
| weak positive | 0 (0%) | 2 (4%) |  |
| positive | 2 (3%) | 1 (2%) |  |
| **Current flow T cell (N (%))** |  |  | 0.72 |
| < 2 SD compatible | 57 (89%) | 48 (94%) |  |
| 2-3 SD compatible | 4 (6%) | 2 (4%) |  |
| weak positive | 1 (2%) | 0 (0%) |  |
| positive | 2 (3%) | 1 (2%) |  |
| **Induction treatment (N (%))** |  |  |  |
| No Induction | 0 (0%) | 0 (0%) | 1.000 |
| Basiliximab | 17 (12.0%) | 11 (10.6%) | 0.73 |
| Thymoglobulin | 120 (84.5%) | 92 (88.5%) | 0.37 |
| Steroid | 142 (100.0%) | 104 (100.0%) | 1.000 |
| Alemtuzumab | 4 (2.8%) | 2 (1.9%) | 0.65 |
| **Outcomes** |  |  |  |
| **Proportion of Antibody Mediated Rejection of the subset of patient underwent biopsy (N (%))** |  |  | 0.063 |
| No | 113 (79.6%) | 72 (69.2%) |  |
| Yes | 29 (20.4%) | 32 (30.8%) |  |
| **Proportion of rejection of the subset of patient underwent biopsy (N (%))** |  |  | 0.016 |
| No | 94 (66.2%) | 53 (51.0%) |  |
| Yes | 48 (33.8%) | 51 (49.0%) |  |
| **Proportion of T-cell Mediated Rejection of the subset of patient underwent biopsy (N (%))** |  |  | 0.045 |
| No | 102 (71.8%) | 62 (59.6%) |  |
| Yes | 40 (28.2%) | 42 (40.4%) |  |
| **Post-Transplant Donor Specific Antibodies (N (%))** |  |  | 0.12 |
| No | 95 (67.4%) | 60 (57.7%) |  |
| Yes | 46 (32.6%) | 44 (42.3%) |  |
| **Dd-cf-DNA elevation (N (%))** |  |  | 0.020 |
| No | 71 (68.9%) | 40 (51.9%) |  |
| Yes | 32 (31.1%) | 37 (48.1%) |  |
| **Primary outcome (N (%))** |  |  | 0.034 |
| No | 64 (45.1%) | 33 (31.7%) |  |
| Yes | 78 (54.9%) | 71 (68.3%) |  |
| **Delayed Graft Function (N (%))** |  |  | 0.64 |
| No | 117 (82.4%) | 88 (84.6%) |  |
| Yes | 25 (17.6%) | 16 (15.4%) |  |
| **Death (N (%))** |  |  | 0.90 |
| No | 133 (93.7%) | 97 (93.3%) |  |
| Yes | 9 (6.3%) | 7 (6.7%) |  |
| **Graft Loss (N (%))** |  |  | 0.77 |
| No | 134 (94.4%) | 99 (95.2%) |  |
| Yes | 8 (5.6%) | 5 (4.8%) |  |

Values are expressed as mean (standard deviation), median (interquartile range), or number (%). Continuous variables were compared via t-tests or Mann-Whitney U tests. Categorical variables were compared via Chi-square tests.

**Table S10:** Patient characteristics of the rejection cohort based on PIRCHE-B score

|  | **Low Risk PIRCHE-B Score Group** | **High Risk PIRCHE-B Score Group** | **p-value** |
| --- | --- | --- | --- |
| **N** | 97 (39%) | 149 (61%) |  |
| **Recipients’ characteristics** |  |  |  |
| **Age (years), mean (SD)** | 48.7 (15.0) | 49.0 (15.0) | 0.85 |
| **Gender (N (%))** |  |  | 0.76 |
| male | 60 (61.9%) | 95 (63.8%) |  |
| female | 37 (38.1%) | 54 (36.2%) |  |
| **Race/ethnicity (N (%))** |  |  | 0.038 |
| White | 64 (66.0%) | 95 (63.8%) |  |
| Hispanic | 24 (24.7%) | 23 (15.4%) |  |
| African American | 0 (0.0%) | 5 (3.4%) |  |
| Asian | 1 (1.0%) | 9 (6.0%) |  |
| Other | 8 (8.2%) | 17 (11.4%) |  |
| Unknown | 0 (0%) | 0 (0%) |  |
| **Body Mass Index (kg/m2), mean (SD)** | 28.8 (6.0) | 29.1 (6.0) | 0.67 |
| **Dialysis vintage (months), median (IQR)** | 28.8 (13.2-51.9) | 42.5 (23.8-63.2) | 0.004 |
| **Prior kidney transplant (N (%))** |  |  | <0.001 |
| No | 73 (75.3%) | 138 (92.6%) |  |
| Yes | 24 (24.7%) | 11 (7.4%) |  |
| **Multi-organ transplantation (N (%))** |  |  | 0.29 |
| Liver + kidney | 2 (2.1%) | 1 (0.7%) |  |
| Pancreas + kidney | 1 (1.0%) | 2 (1.3%) |  |
| Heart + kidney | 0 (0.0%) | 3 (2.0%) |  |
| None | 94 (96.9%) | 140 (94.0%) |  |
| Liver +Heart+Kidney | 0 (0.0%) | 3 (2.0%) |  |
| **CMV risk categories (N (%))** |  |  | 0.40 |
| Low | 22 (22.7%) | 36 (24.2%) |  |
| Intermediate | 47 (48.5%) | 57 (38.3%) |  |
| High | 27 (27.8%) | 53 (35.6%) |  |
| Unknow | 1 (1.0%) | 3 (2.0%) |  |
| **Was the donor organ pumped? (N (%))** |  |  | 0.42 |
| No | 25 (27.2%) | 32 (22.5%) |  |
| Yes | 67 (72.8%) | 110 (77.5%) |  |
| **En block kidney (N (%))** | 0 (0%) | 7 (27%) | 0.027 |
| **Dual kidney (N (%))** | 1 (7%) | 4 (15%) | 0.41 |
| **Transplantation characteristics** |  |  |  |
| **Cold ischemic time (hours), median (IQR)** | 17.2 (7.4-22.7) | 14.8 (7.2-21.0) | 0.41 |
| **cPRA (%), median (IQR)** | 0 (0-76) | 0 (0-0) | <0.001 |
| **Donors’ characteristics** |  |  |  |
| **Age (years), mean (SD)** | 39.1 (15.9) | 38.0 (16.6) | 0.62 |
| **Gender (N (%))** |  |  | 0.49 |
| male | 53 (54.6%) | 88 (59.1%) |  |
| female | 44 (45.4%) | 61 (40.9%) |  |
| **Race/ethnicity** |  |  | 0.71 |
| White | 77 (79.4%) | 121 (81.2%) |  |
| African American | 5 (5.2%) | 4 (2.7%) |  |
| Asian | 2 (2.1%) | 4 (2.7%) |  |
| Other | 3 (3.1%) | 2 (1.3%) |  |
| Unknown | 10 (10.3%) | 18 (12.1%) |  |
| **Donor type (N (%))** |  |  | 0.44 |
| Living | 27 (27.8%) | 35 (23.5%) |  |
| Deceased | 70 (72.2%) | 114 (76.5%) |  |
| **Donor DCD (N (%))** |  |  | 0.92 |
| No | 38 (54.3%) | 61 (53.5%) |  |
| Yes | 32 (45.7%) | 53 (46.5%) |  |
| **KDPI, median (IQR)** | 27 (14-51) | 35 (21-63) | 0.058 |
| **Donor cause of death (N (%))** |  |  | 0.96 |
| Anoxia | 34 (48.6%) | 60 (52.6%) |  |
| Cerebrovascular/stroke | 10 (14.3%) | 17 (14.9%) |  |
| Head trauma | 23 (32.9%) | 34 (29.8%) |  |
| Central nervous system tumor | 1 (1.4%) | 1 (0.9%) |  |
| Other | 2 (2.9%) | 2 (1.8%) |  |
| **Donor terminal creatinine (mg/dl), median (IQR)** | 0.80 (0.61-1.02) | 0.84 (0.65-1.05) | 0.37 |
| **Donors diabetes (N (%))** |  |  | 0.40 |
| No | 89 (91.8%) | 137 (94.5%) |  |
| Yes | 8 (8.2%) | 8 (5.5%) |  |
| **Donors hypertension (N (%))** |  |  | 0.26 |
| No | 81 (84.4%) | 113 (78.5%) |  |
| Yes | 15 (15.6%) | 31 (21.5%) |  |
| **Donor malignancy (N (%))** |  |  | 0.59 |
| No | 94 (96.9%) | 146 (98.0%) |  |
| Yes | 3 (3.1%) | 3 (2.0%) |  |
| **Immunological characteristics** |  |  |  |
| **Number of HLA mismatches (HLA A,B and DR) (N (%))** |  |  | <0.001 |
| 0 | 9 (9.3%) | 0 (0.0%) |  |
| 1 | 6 (6.2%) | 0 (0.0%) |  |
| 2 | 10 (10.3%) | 3 (2.0%) |  |
| 3 | 26 (26.8%) | 14 (9.4%) |  |
| 4 | 24 (24.7%) | 33 (22.1%) |  |
| 5 | 16 (16.5%) | 61 (40.9%) |  |
| 6 | 6 (6.2%) | 38 (25.5%) |  |
| **HLA mismatches A (N (%))** |  |  | 0.006 |
| 0 | 2 (9%) | 2 (8%) |  |
| 1 | 15 (68%) | 6 (24%) |  |
| 2 | 5 (23%) | 17 (68%) |  |
| **HLA mismatches B (N (%))** |  |  | 0.14 |
| 0 | 1 (5%) | 2 (8%) |  |
| 1 | 8 (36%) | 3 (12%) |  |
| 2 | 13 (59%) | 20 (80%) |  |
| **HLA mismatches DR (N (%))** |  |  | <0.001 |
| 0 | 14 (34%) | 3 (4%) |  |
| 1 | 18 (44%) | 26 (39%) |  |
| 2 | 9 (22%) | 38 (57%) |  |
| **HLA mismatches DQ (N (%))** |  |  | <0.001 |
| 0 | 12 (55%) | 1 (4%) |  |
| 1 | 10 (45%) | 12 (48%) |  |
| 2 | 0 (0%) | 12 (48%) |  |
| **Current flow B cell (N (%))** |  |  | 0.14 |
| < 2 SD compatible | 36 (84%) | 69 (96%) |  |
| 2-3 SD compatible | 4 (9%) | 1 (1%) |  |
| weak positive | 1 (2%) | 1 (1%) |  |
| positive | 2 (5%) | 1 (1%) |  |
| **Current flow T cell (N (%))** |  |  | 0.14 |
| < 2 SD compatible | 36 (84%) | 69 (96%) |  |
| 2-3 SD compatible | 4 (9%) | 2 (3%) |  |
| weak positive | 1 (2%) | 0 (0%) |  |
| positive | 2 (5%) | 1 (1%) |  |
| **Induction treatment (N (%))** |  |  |  |
| No Induction | 0 (0%) | 0 (0%) | 1.000 |
| Basiliximab | 11 (11.3%) | 17 (11.4%) | 0.99 |
| Thymoglobulin | 80 (82.5%) | 132 (88.6%) | 0.17 |
| Steroid | 97 (100.0%) | 149 (100.0%) | 1.000 |
| Alemtuzumab | 5 (5.2%) | 1 (0.7%) | 0.026 |
| **Outcomes** |  |  |  |
| **Proportion of Antibody Mediated Rejection of the subset of patient underwent biopsy (N (%))** |  |  | 0.002 |
| No | 83 (85.6%) | 102 (68.5%) |  |
| Yes | 14 (14.4%) | 47 (31.5%) |  |
| **Proportion of rejection of the subset of patient underwent biopsy (N (%))** |  |  | 0.008 |
| No | 68 (70.1%) | 79 (53.0%) |  |
| Yes | 29 (29.9%) | 70 (47.0%) |  |
| **Proportion of T-cell Mediated Rejection of the subset of patient underwent biopsy (N (%))** |  |  | 0.080 |
| No | 71 (73.2%) | 93 (62.4%) |  |
| Yes | 26 (26.8%) | 56 (37.6%) |  |
| **Post-Transplant Donor Specific Antibodies (N (%))** |  |  | 0.002 |
| No | 72 (75.0%) | 83 (55.7%) |  |
| Yes | 24 (25.0%) | 66 (44.3%) |  |
| **Dd-cf-DNA elevation (N (%))** |  |  | 0.071 |
| No | 47 (70.1%) | 64 (56.6%) |  |
| Yes | 20 (29.9%) | 49 (43.4%) |  |
| **Primary outcome (N (%))** |  |  | <0.001 |
| No | 52 (53.6%) | 45 (30.2%) |  |
| Yes | 45 (46.4%) | 104 (69.8%) |  |
| **Delayed Graft Function (N (%))** |  |  | 0.32 |
| No | 78 (80.4%) | 127 (85.2%) |  |
| Yes | 19 (19.6%) | 22 (14.8%) |  |
| **Death (N (%))** |  |  | 0.37 |
| No | 89 (91.8%) | 141 (94.6%) |  |
| Yes | 8 (8.2%) | 8 (5.4%) |  |
| **Graft Loss (N (%))** |  |  | 0.61 |
| No | 91 (93.8%) | 142 (95.3%) |  |
| Yes | 6 (6.2%) | 7 (4.7%) |  |

Values are expressed as mean (standard deviation), median (interquartile range), or number (%). Continuous variables were compared via t-tests or Mann-Whitney U tests. Categorical variables were compared via Chi-square tests.

**Table S11:** Performance characteristics table of PIRCHE-T2 for rejection

| Cutpoint | Sensitivity | Specificity | Overall % correctly classified | LR+ | LR- |
| --- | --- | --- | --- | --- | --- |
| ( >= 0 ) | 100.00% | 0.00% | 40.24% | 1 |  |
| ( >= 7 ) | 100.00% | 4.76% | 43.09% | 1.05 | 0 |
| ( >= 8 ) | 98.99% | 4.76% | 42.68% | 1.0394 | 0.2121 |
| ( >= 9 ) | 98.99% | 5.44% | 43.09% | 1.0469 | 0.1856 |
| ( >= 11 ) | 98.99% | 6.12% | 43.50% | 1.0545 | 0.165 |
| ( >= 15 ) | 98.99% | 7.48% | 44.31% | 1.07 | 0.135 |
| ( >= 17 ) | 97.98% | 7.48% | 43.90% | 1.059 | 0.27 |
| ( >= 19 ) | 97.98% | 8.16% | 44.31% | 1.0669 | 0.2475 |
| ( >= 19.04 ) | 96.97% | 8.16% | 43.90% | 1.0559 | 0.3712 |
| ( >= 20 ) | 95.96% | 8.16% | 43.50% | 1.0449 | 0.4949 |
| ( >= 21 ) | 95.96% | 8.84% | 43.90% | 1.0527 | 0.4569 |
| ( >= 22 ) | 94.95% | 10.88% | 44.72% | 1.0655 | 0.464 |
| ( >= 23 ) | 94.95% | 11.56% | 45.12% | 1.0737 | 0.4367 |
| ( >= 26 ) | 92.93% | 12.24% | 44.72% | 1.059 | 0.5774 |
| ( >= 28 ) | 91.92% | 12.24% | 44.31% | 1.0475 | 0.6599 |
| ( >= 29 ) | 90.91% | 14.29% | 45.12% | 1.0606 | 0.6364 |
| ( >= 30 ) | 90.91% | 14.97% | 45.53% | 1.0691 | 0.6074 |
| ( >= 31 ) | 89.90% | 15.65% | 45.53% | 1.0657 | 0.6456 |
| ( >= 32 ) | 88.89% | 16.33% | 45.53% | 1.0623 | 0.6806 |
| ( >= 33 ) | 87.88% | 17.01% | 45.53% | 1.0589 | 0.7127 |
| ( >= 34 ) | 87.88% | 17.69% | 45.93% | 1.0676 | 0.6853 |
| ( >= 35.65 ) | 85.86% | 18.37% | 45.53% | 1.0518 | 0.7699 |
| ( >= 36 ) | 85.86% | 19.05% | 45.93% | 1.0606 | 0.7424 |
| ( >= 36.47 ) | 84.85% | 21.77% | 47.15% | 1.0846 | 0.696 |
| ( >= 37 ) | 84.85% | 22.45% | 47.56% | 1.0941 | 0.6749 |
| ( >= 38.54 ) | 83.84% | 24.49% | 48.37% | 1.1103 | 0.6599 |
| ( >= 39 ) | 82.83% | 24.49% | 47.97% | 1.0969 | 0.7012 |
| ( >= 40 ) | 82.83% | 25.17% | 48.37% | 1.1069 | 0.6822 |
| ( >= 41 ) | 82.83% | 26.53% | 49.19% | 1.1274 | 0.6472 |
| ( >= 43 ) | 81.82% | 27.89% | 49.59% | 1.1346 | 0.6519 |
| ( >= 44 ) | 80.81% | 29.25% | 50.00% | 1.1422 | 0.6561 |
| ( >= 45 ) | 79.80% | 29.25% | 49.59% | 1.1279 | 0.6906 |
| ( >= 45.35 ) | 78.79% | 31.97% | 50.81% | 1.1582 | 0.6634 |
| ( >= 45.78 ) | 78.79% | 32.65% | 51.22% | 1.1699 | 0.6496 |
| ( >= 46 ) | 78.79% | 33.33% | 51.63% | 1.1818 | 0.6364 |
| ( >= 48 ) | 77.78% | 34.01% | 51.63% | 1.1787 | 0.6533 |
| ( >= 49 ) | 77.78% | 36.05% | 52.85% | 1.2163 | 0.6164 |
| ( >= 50 ) | 75.76% | 37.41% | 52.85% | 1.2105 | 0.6479 |
| ( >= 51 ) | 73.74% | 41.50% | 54.47% | 1.2604 | 0.6329 |
| ( >= 52 ) | 71.72% | 42.18% | 54.07% | 1.2403 | 0.6706 |
| ( >= 53 ) | 69.70% | 42.86% | 53.66% | 1.2197 | 0.7071 |
| ( >= 54.3 ) | 67.68% | 43.54% | 53.25% | 1.1986 | 0.7424 |
| ( >= 55 ) | 66.67% | 43.54% | 52.85% | 1.1807 | 0.7656 |
| ( >= 55.89 ) | 64.65% | 46.94% | 54.07% | 1.2183 | 0.7532 |
| ( >= 56 ) | 64.65% | 47.62% | 54.47% | 1.2342 | 0.7424 |
| ( >= 57 ) | 64.65% | 48.98% | 55.28% | 1.2671 | 0.7218 |
| ( >= 57.04 ) | 59.60% | 51.70% | 54.88% | 1.2339 | 0.7815 |
| ( >= 58 ) | 58.59% | 51.70% | 54.47% | 1.213 | 0.801 |
| ( >= 59 ) | 56.57% | 51.70% | 53.66% | 1.1711 | 0.8401 |
| ( >= 60 ) | 54.55% | 51.70% | 52.85% | 1.1293 | 0.8792 |
| ( >= 61 ) | 54.55% | 55.10% | 54.88% | 1.2149 | 0.8249 |
| ( >= 62 ) | 53.54% | 57.82% | 56.10% | 1.2693 | 0.8036 |
| ( >= 63 ) | 52.53% | 58.50% | 56.10% | 1.2658 | 0.8115 |
| ( >= 64 ) | 52.53% | 60.54% | 57.32% | 1.3312 | 0.7841 |
| ( >= 65 ) | 52.53% | 61.22% | 57.72% | 1.3546 | 0.7754 |
| ( >= 66 ) | 51.52% | 63.95% | 58.94% | 1.4288 | 0.7582 |
| ( >= 66.94 ) | 48.48% | 65.31% | 58.54% | 1.3975 | 0.7888 |
| ( >= 67 ) | 48.48% | 65.99% | 58.94% | 1.4255 | 0.7807 |
| ( >= 67.07 ) | 46.46% | 67.35% | 58.94% | 1.423 | 0.7949 |
| ( >= 68 ) | 46.46% | 68.03% | 59.35% | 1.4533 | 0.787 |
| ( >= 69 ) | 43.43% | 70.75% | 59.76% | 1.4848 | 0.7995 |
| ( >= 70 ) | 42.42% | 71.43% | 59.76% | 1.4848 | 0.8061 |
| ( >= 71 ) | 39.39% | 71.43% | 58.54% | 1.3788 | 0.8485 |
| ( >= 72 ) | 37.37% | 72.79% | 58.54% | 1.3735 | 0.8604 |
| ( >= 73 ) | 36.36% | 72.79% | 58.13% | 1.3364 | 0.8743 |
| ( >= 74 ) | 32.32% | 73.47% | 56.91% | 1.2183 | 0.9212 |
| ( >= 75 ) | 31.31% | 74.15% | 56.91% | 1.2113 | 0.9263 |
| ( >= 76 ) | 30.30% | 75.51% | 57.32% | 1.2374 | 0.923 |
| ( >= 77 ) | 27.27% | 76.87% | 56.91% | 1.1791 | 0.9461 |
| ( >= 78 ) | 24.24% | 78.91% | 56.91% | 1.1496 | 0.96 |
| ( >= 79 ) | 23.23% | 78.91% | 56.50% | 1.1017 | 0.9728 |
| ( >= 80 ) | 22.22% | 79.59% | 56.50% | 1.0889 | 0.9772 |
| ( >= 81 ) | 20.20% | 80.95% | 56.50% | 1.0606 | 0.9857 |
| ( >= 82 ) | 17.17% | 82.31% | 56.10% | 0.9709 | 1.0063 |
| ( >= 83 ) | 17.17% | 83.67% | 56.91% | 1.0518 | 0.9899 |
| ( >= 83.34 ) | 17.17% | 84.35% | 57.32% | 1.0975 | 0.9819 |
| ( >= 83.69 ) | 17.17% | 85.03% | 57.72% | 1.1474 | 0.9741 |
| ( >= 84 ) | 17.17% | 85.71% | 58.13% | 1.202 | 0.9663 |
| ( >= 85 ) | 16.16% | 85.71% | 57.72% | 1.1313 | 0.9781 |
| ( >= 86 ) | 15.15% | 85.71% | 57.32% | 1.0606 | 0.9899 |
| ( >= 87 ) | 15.15% | 86.39% | 57.72% | 1.1136 | 0.9821 |
| ( >= 88 ) | 15.15% | 87.07% | 58.13% | 1.1722 | 0.9744 |
| ( >= 89 ) | 15.15% | 87.76% | 58.54% | 1.2374 | 0.9669 |
| ( >= 89.31 ) | 12.12% | 89.80% | 58.54% | 1.1879 | 0.9787 |
| ( >= 90 ) | 12.12% | 90.48% | 58.94% | 1.2727 | 0.9713 |
| ( >= 91 ) | 12.12% | 92.52% | 60.16% | 1.6198 | 0.9499 |
| ( >= 91.07 ) | 11.11% | 93.20% | 60.16% | 1.6333 | 0.9538 |
| ( >= 93 ) | 10.10% | 93.20% | 59.76% | 1.4848 | 0.9646 |
| ( >= 95 ) | 9.09% | 94.56% | 60.16% | 1.6705 | 0.9614 |
| ( >= 96 ) | 8.08% | 94.56% | 59.76% | 1.4848 | 0.9721 |
| ( >= 97 ) | 8.08% | 95.24% | 60.16% | 1.697 | 0.9652 |
| ( >= 98 ) | 6.06% | 95.24% | 59.35% | 1.2727 | 0.9864 |
| ( >= 99 ) | 6.06% | 95.92% | 59.76% | 1.4848 | 0.9794 |
| ( >= 100 ) | 6.06% | 96.60% | 60.16% | 1.7818 | 0.9725 |
| ( >= 102 ) | 6.06% | 97.28% | 60.57% | 2.2273 | 0.9657 |
| ( >= 103 ) | 5.05% | 97.96% | 60.57% | 2.4747 | 0.9693 |
| ( >= 106 ) | 4.04% | 97.96% | 60.16% | 1.9798 | 0.9796 |
| ( >= 109 ) | 4.04% | 98.64% | 60.57% | 2.9697 | 0.9728 |
| ( >= 110 ) | 3.03% | 99.32% | 60.57% | 4.4545 | 0.9763 |
| ( >= 118 ) | 3.03% | 100.00% | 60.98% |  | 0.9697 |
| ( >= 128 ) | 2.02% | 100.00% | 60.57% |  | 0.9798 |
| ( >= 137 ) | 1.01% | 100.00% | 60.16% |  | 0.9899 |
| ( > 137 ) | 0.00% | 100.00% | 59.76% |  | 1 |

LR: Likelihood ratio

**Table S12:** Performance characteristics table of PIRCHE-B for rejection

| Cutpoint | Sensitivity | Specificity | Overall % correctly classified | LR+ | LR- |
| --- | --- | --- | --- | --- | --- |
| ( >= 0 ) | 100.00% | 0.00% | 40.24% | 1 |  |
| ( >= 1 ) | 100.00% | 4.76% | 43.09% | 1.05 | 0 |
| ( >= 2 ) | 98.99% | 6.12% | 43.50% | 1.0545 | 0.165 |
| ( >= 3 ) | 98.99% | 8.16% | 44.72% | 1.0779 | 0.1237 |
| ( >= 4 ) | 95.96% | 8.84% | 43.90% | 1.0527 | 0.4569 |
| ( >= 5 ) | 95.96% | 10.88% | 45.12% | 1.0768 | 0.3712 |
| ( >= 6 ) | 95.96% | 14.97% | 47.56% | 1.1285 | 0.27 |
| ( >= 7 ) | 91.92% | 22.45% | 50.41% | 1.1853 | 0.36 |
| ( >= 8 ) | 88.89% | 27.21% | 52.03% | 1.2212 | 0.4083 |
| ( >= 9 ) | 84.85% | 30.61% | 52.44% | 1.2228 | 0.4949 |
| ( >= 9.02 ) | 78.79% | 35.37% | 52.85% | 1.2191 | 0.5997 |
| ( >= 10 ) | 77.78% | 35.37% | 52.44% | 1.2035 | 0.6282 |
| ( >= 10.71 ) | 74.75% | 40.82% | 54.47% | 1.263 | 0.6187 |
| ( >= 11 ) | 74.75% | 41.50% | 54.88% | 1.2777 | 0.6085 |
| ( >= 12 ) | 70.71% | 46.26% | 56.10% | 1.3157 | 0.6332 |
| ( >= 13 ) | 64.65% | 50.34% | 56.10% | 1.3018 | 0.7023 |
| ( >= 13.17 ) | 55.56% | 52.38% | 53.66% | 1.1667 | 0.8485 |
| ( >= 14 ) | 54.55% | 52.38% | 53.25% | 1.1455 | 0.8678 |
| ( >= 15 ) | 41.41% | 57.14% | 50.81% | 0.9663 | 1.0253 |
| ( >= 16 ) | 34.34% | 61.90% | 50.81% | 0.9015 | 1.0606 |
| ( >= 17 ) | 32.32% | 65.99% | 52.44% | 0.9503 | 1.0256 |
| ( >= 18 ) | 29.29% | 70.75% | 54.07% | 1.0014 | 0.9994 |
| ( >= 19 ) | 26.26% | 75.51% | 55.69% | 1.0724 | 0.9765 |
| ( >= 20 ) | 25.25% | 78.23% | 56.91% | 1.16 | 0.9555 |
| ( >= 21 ) | 18.18% | 81.63% | 56.10% | 0.9899 | 1.0023 |
| ( >= 22 ) | 11.11% | 85.71% | 55.69% | 0.7778 | 1.037 |
| ( >= 23 ) | 11.11% | 88.44% | 57.32% | 0.9608 | 1.0051 |
| ( >= 24 ) | 8.08% | 90.48% | 57.32% | 0.8485 | 1.0159 |
| ( >= 25 ) | 8.08% | 91.16% | 57.72% | 0.9138 | 1.0084 |
| ( >= 26 ) | 7.07% | 93.88% | 58.94% | 1.1549 | 0.9899 |
| ( >= 27 ) | 5.05% | 94.56% | 58.54% | 0.928 | 1.0041 |
| ( >= 28 ) | 4.04% | 96.60% | 59.35% | 1.1879 | 0.9934 |
| ( >= 30 ) | 3.03% | 98.64% | 60.16% | 2.2273 | 0.9831 |
| ( >= 32 ) | 2.02% | 99.32% | 60.16% | 2.9697 | 0.9865 |
| ( >= 34 ) | 2.02% | 100.00% | 60.57% |  | 0.9798 |
| ( >= 50 ) | 1.01% | 100.00% | 60.16% |  | 0.9899 |
| ( > 50 ) | 0.00% | 100.00% | 59.76% |  | 1 |

LR: Likelihood ratio

**Table S13:** Patient characteristics of the donor-derived cell-free DNA cohort based on PIRCHE-T2 score

|  | **Low Risk PIRCHE-T2 Score Group** | **High Risk PIRCHE-T2 Score Group** | **p-value** |
| --- | --- | --- | --- |
| **N** | 192 (36%) | 337 (64%) |  |
| **Recipients’ characteristics** |  |  |  |
| **Age (years), mean (SD)** | 52.0 (15.5) | 50.0 (14.7) | 0.13 |
| **Gender (N (%))** |  |  | 0.028 |
| male | 105 (54.7%) | 217 (64.4%) |  |
| female | 87 (45.3%) | 120 (35.6%) |  |
| **Race/ethnicity (N (%))** |  |  | 0.002 |
| White | 147 (76.6%) | 199 (59.1%) |  |
| Hispanic | 28 (14.6%) | 76 (22.6%) |  |
| African American | 1 (0.5%) | 11 (3.3%) |  |
| Asian | 4 (2.1%) | 16 (4.7%) |  |
| Other | 12 (6.2%) | 34 (10.1%) |  |
| Unknown | 0 (0.0%) | 1 (0.3%) |  |
| **Body Mass Index (kg/m2), mean (SD)** | 28.8 (5.6) | 29.2 (5.5) | 0.43 |
| **Dialysis vintage (months), median (IQR)** | 28.4 (13.7-45.7) | 36.7 (17.7-62.1) | 0.006 |
| **Prior kidney transplant (N (%))** |  |  | 0.57 |
| No | 190 (99.0%) | 335 (99.4%) |  |
| Yes | 2 (1.0%) | 2 (0.6%) |  |
| **Multi-organ transplantation (N (%))** |  |  | 0.28 |
| Liver + kidney | 0 (0.0%) | 0 (0.0%) |  |
| Pancreas + kidney | 0 (0.0%) | 2 (0.6%) |  |
| Heart + kidney | 0 (0.0%) | 0 (0.0%) |  |
| None | 192 (100.0%) | 335 (99.4%) |  |
| Liver +Heart+Kidney | 0 (0.0%) | 0 (0.0%) |  |
| **CMV risk categories (N (%))** |  |  | 0.069 |
| Low | 46 (24.0%) | 87 (25.8%) |  |
| Intermediate | 77 (40.1%) | 157 (46.6%) |  |
| High | 57 (29.7%) | 85 (25.2%) |  |
| Unknow | 12 (6.2%) | 8 (2.4%) |  |
| **Was the donor organ pumped? (N (%))** |  |  | <0.001 |
| No | 78 (43.6%) | 89 (28.1%) |  |
| Yes | 101 (56.4%) | 228 (71.9%) |  |
| **En block kidney (N (%))** | 0 (0%) | 0 (0%) | 1.000 |
| **Dual kidney (N (%))** | 0 (0%) | 0 (0%) | 1.000 |
| **Transplantation characteristics** |  |  |  |
| **Cold ischemic time (hours), median (IQR)** | 10.2 (3.1-20.3) | 13.7 (5.4-19.4) | 0.017 |
| **cPRA (%), median (IQR)** | 0 (0-0) | 0 (0-0) | 0.77 |
| **Donors’ characteristics** |  |  |  |
| **Age (years), mean (SD)** | 39.1 (14.3) | 37.9 (14.5) | 0.37 |
| **Gender (N (%))** |  |  | 0.002 |
| male | 82 (42.7%) | 192 (57.0%) |  |
| female | 110 (57.3%) | 145 (43.0%) |  |
| **Race/ethnicity** |  |  | 0.50 |
| White | 164 (85.4%) | 272 (80.7%) |  |
| African American | 3 (1.6%) | 8 (2.4%) |  |
| Asian | 3 (1.6%) | 7 (2.1%) |  |
| Other | 4 (2.1%) | 4 (1.2%) |  |
| Unknown | 18 (9.4%) | 46 (13.6%) |  |
| **Donor type (N (%))** |  |  | 0.001 |
| Living | 87 (45.3%) | 106 (31.5%) |  |
| Deceased | 105 (54.7%) | 231 (68.5%) |  |
| **Donor DCD (N (%))** |  |  | 0.62 |
| No | 59 (56.2%) | 123 (53.2%) |  |
| Yes | 46 (43.8%) | 108 (46.8%) |  |
| **KDPI, median (IQR)** | 31 (15-46) | 26 (13-51) | 0.83 |
| **Donor cause of death (N (%))** |  |  | 0.95 |
| Anoxia | 53 (50.5%) | 120 (51.9%) |  |
| Cerebrovascular/stroke | 18 (17.1%) | 34 (14.7%) |  |
| Head trauma | 30 (28.6%) | 70 (30.3%) |  |
| Central nervous system tumor | 1 (1.0%) | 1 (0.4%) |  |
| Other | 3 (2.9%) | 6 (2.6%) |  |
| **Donor terminal creatinine (mg/dl), median (IQR)** | 0.81 (0.66-1.01) | 0.83 (0.67-1.02) | 0.54 |
| **Donors diabetes (N (%))** |  |  | 0.63 |
| No | 185 (96.4%) | 316 (95.5%) |  |
| Yes | 7 (3.6%) | 15 (4.5%) |  |
| **Donors hypertension (N (%))** |  |  | 0.033 |
| No | 172 (90.1%) | 274 (83.3%) |  |
| Yes | 19 (9.9%) | 55 (16.7%) |  |
| **Donor malignancy (N (%))** |  |  | 0.70 |
| No | 187 (97.4%) | 330 (97.9%) |  |
| Yes | 5 (2.6%) | 7 (2.1%) |  |
| **Immunological characteristics** |  |  |  |
| **Number of HLA mismatches (HLA A,B and DR) (N (%))** |  |  | <0.001 |
| 0 | 17 (8.9%) | 0 (0.0%) |  |
| 1 | 11 (5.7%) | 0 (0.0%) |  |
| 2 | 25 (13.0%) | 4 (1.2%) |  |
| 3 | 63 (32.8%) | 34 (10.1%) |  |
| 4 | 41 (21.4%) | 82 (24.3%) |  |
| 5 | 28 (14.6%) | 141 (41.8%) |  |
| 6 | 7 (3.6%) | 76 (22.6%) |  |
| **HLA mismatches A (N (%))** |  |  | <0.001 |
| 0 | 11 (26%) | 6 (7%) |  |
| 1 | 21 (50%) | 31 (36%) |  |
| 2 | 10 (24%) | 50 (57%) |  |
| **HLA mismatches B (N (%))** |  |  | 0.002 |
| 0 | 6 (14%) | 1 (1%) |  |
| 1 | 14 (33%) | 21 (24%) |  |
| 2 | 22 (52%) | 65 (75%) |  |
| **HLA mismatches DR (N (%))** |  |  | <0.001 |
| 0 | 24 (24.5%) | 11 (6.6%) |  |
| 1 | 57 (58.2%) | 76 (45.8%) |  |
| 2 | 17 (17.3%) | 79 (47.6%) |  |
| **HLA mismatches DQ (N (%))** |  |  | 0.002 |
| 0 | 15 (36%) | 9 (10%) |  |
| 1 | 17 (40%) | 41 (48%) |  |
| 2 | 10 (24%) | 36 (42%) |  |
| **Current flow B cell (N (%))** |  |  | 0.73 |
| < 2 SD compatible | 92 (94.8%) | 163 (94.2%) |  |
| 2-3 SD compatible | 3 (3.1%) | 4 (2.3%) |  |
| weak positive | 0 (0.0%) | 2 (1.2%) |  |
| positive | 2 (2.1%) | 4 (2.3%) |  |
| **Current flow T cell (N (%))** |  |  | 0.18 |
| < 2 SD compatible | 91 (93.8%) | 167 (96.5%) |  |
| 2-3 SD compatible | 4 (4.1%) | 2 (1.2%) |  |
| weak positive | 1 (1.0%) | 0 (0.0%) |  |
| positive | 1 (1.0%) | 4 (2.3%) |  |
| **Induction treatment (N (%))** |  |  |  |
| No Induction | 0 (0%) | 0 (0%) | 1.000 |
| Basiliximab | 33 (17.2%) | 34 (10.1%) | 0.018 |
| Thymoglobulin | 152 (79.2%) | 300 (89.0%) | 0.002 |
| Steroid | 192 (100.0%) | 336 (99.7%) | 0.45 |
| Alemtuzumab | 2 (1.0%) | 4 (1.2%) | 0.88 |
| **Outcomes** |  |  |  |
| **Proportion of Antibody Mediated Rejection of the subset of patient underwent biopsy (N (%))** |  |  | 0.51 |
| No | 41 (78.8%) | 95 (74.2%) |  |
| Yes | 11 (21.2%) | 33 (25.8%) |  |
| **Proportion of rejection of the subset of patient underwent biopsy (N (%))** |  |  | 0.064 |
| No | 37 (71.2%) | 72 (56.2%) |  |
| Yes | 15 (28.8%) | 56 (43.8%) |  |
| **Proportion of T-cell Mediated Rejection of the subset of patient underwent biopsy (N (%))** |  |  | 0.063 |
| No | 40 (76.9%) | 80 (62.5%) |  |
| Yes | 12 (23.1%) | 48 (37.5%) |  |
| **Post-Transplant Donor Specific Antibodies (N (%))** |  |  | 0.011 |
| No | 155 (84.2%) | 249 (74.6%) |  |
| Yes | 29 (15.8%) | 85 (25.4%) |  |
| **Dd-cf-DNA elevation (N (%))** |  |  | <0.001 |
| No | 171 (89.1%) | 248 (73.6%) |  |
| Yes | 21 (10.9%) | 89 (26.4%) |  |
| **Primary outcome (N (%))** |  |  | <0.001 |
| No | 144 (75.0%) | 192 (57.0%) |  |
| Yes | 48 (25.0%) | 145 (43.0%) |  |
| **Delayed Graft Function (N (%))** |  |  | 0.90 |
| No | 180 (93.8%) | 315 (93.5%) |  |
| Yes | 12 (6.2%) | 22 (6.5%) |  |
| **Death (N (%))** |  |  | 0.37 |
| No | 186 (96.9%) | 321 (95.3%) |  |
| Yes | 6 (3.1%) | 16 (4.7%) |  |
| **Graft Loss (N (%))** |  |  | 0.88 |
| No | 190 (99.0%) | 333 (98.8%) |  |
| Yes | 2 (1.0%) | 4 (1.2%) |  |

Values are expressed as mean (standard deviation), median (interquartile range), or number (%). Continuous variables were compared via t-tests or Mann-Whitney U tests. Categorical variables were compared via Chi-square tests.

**Table S14:** Patient characteristics of the donor-derived cell-free DNA cohort based on PIRCHE-B score

|  | **Low Risk PIRCHE-B Score Group** | **High Risk PIRCHE-B Score Group** | **p-value** |
| --- | --- | --- | --- |
| **N** | 163 (31%) | 366 (69%) |  |
| **Recipients’ characteristics** |  |  |  |
| **Age (years), mean (SD)** | 53.2 (15.2) | 49.6 (14.8) | 0.012 |
| **Gender (N (%))** |  |  | 0.23 |
| male | 93 (57.1%) | 229 (62.6%) |  |
| female | 70 (42.9%) | 137 (37.4%) |  |
| **Race/ethnicity (N (%))** |  |  | 0.007 |
| White | 124 (76.1%) | 222 (60.7%) |  |
| Hispanic | 28 (17.2%) | 76 (20.8%) |  |
| African American | 1 (0.6%) | 11 (3.0%) |  |
| Asian | 4 (2.5%) | 16 (4.4%) |  |
| Other | 6 (3.7%) | 40 (10.9%) |  |
| Unknown | 0 (0.0%) | 1 (0.3%) |  |
| **Body Mass Index (kg/m2), mean (SD)** | 28.9 (5.4) | 29.1 (5.6) | 0.63 |
| **Dialysis vintage (months), median (IQR)** | 26.4 (14.0-47.9) | 36.0 (17.7-61.5) | 0.003 |
| **Prior kidney transplant (N (%))** |  |  | 0.80 |
| No | 162 (99.4%) | 363 (99.2%) |  |
| Yes | 1 (0.6%) | 3 (0.8%) |  |
| **Multi-organ transplantation (N (%))** |  |  | 0.34 |
| Liver + kidney | 0 (0.0%) | 0 (0.0%) |  |
| Pancreas + kidney | 0 (0.0%) | 2 (0.5%) |  |
| Heart + kidney | 0 (0.0%) | 0 (0.0%) |  |
| None | 163 (100.0%) | 364 (99.5%) |  |
| Liver +Heart+Kidney | 0 (0.0%) | 0 (0.0%) |  |
| **CMV risk categories (N (%))** |  |  | 1.00 |
| Low | 41 (25.2%) | 92 (25.1%) |  |
| Intermediate | 72 (44.2%) | 162 (44.3%) |  |
| High | 44 (27.0%) | 98 (26.8%) |  |
| Unknow | 6 (3.7%) | 14 (3.8%) |  |
| **Was the donor organ pumped? (N (%))** |  |  | 0.059 |
| No | 60 (39.7%) | 107 (31.0%) |  |
| Yes | 91 (60.3%) | 238 (69.0%) |  |
| **En block kidney (N (%))** | 0 (0%) | 0 (0%) | 1.000 |
| **Dual kidney (N (%))** | 0 (0%) | 0 (0%) | 1.000 |
| **Transplantation characteristics** |  |  |  |
| **Cold ischemic time (hours), median (IQR)** | 10.8 (3.2-19.5) | 13.7 (4.5-19.9) | 0.082 |
| **cPRA (%), median (IQR)** | 0 (0-0) | 0 (0-0) | 0.79 |
| **Donors’ characteristics** |  |  |  |
| **Age (years), mean (SD)** | 39.2 (14.8) | 38.0 (14.3) | 0.37 |
| **Gender (N (%))** |  |  | 0.011 |
| male | 71 (43.6%) | 203 (55.5%) |  |
| female | 92 (56.4%) | 163 (44.5%) |  |
| **Race/ethnicity** |  |  | 0.90 |
| White | 135 (82.8%) | 301 (82.2%) |  |
| African American | 2 (1.2%) | 9 (2.5%) |  |
| Asian | 3 (1.8%) | 7 (1.9%) |  |
| Other | 2 (1.2%) | 6 (1.6%) |  |
| Unknown | 21 (12.9%) | 43 (11.7%) |  |
| **Donor type (N (%))** |  |  | 0.062 |
| Living | 69 (42.3%) | 124 (33.9%) |  |
| Deceased | 94 (57.7%) | 242 (66.1%) |  |
| **Donor DCD (N (%))** |  |  | 0.23 |
| No | 46 (48.9%) | 136 (56.2%) |  |
| Yes | 48 (51.1%) | 106 (43.8%) |  |
| **KDPI, median (IQR)** | 30 (15-54) | 27 (13-48) | 0.55 |
| **Donor cause of death (N (%))** |  |  | 0.69 |
| Anoxia | 49 (52.1%) | 124 (51.2%) |  |
| Cerebrovascular/stroke | 13 (13.8%) | 39 (16.1%) |  |
| Head trauma | 28 (29.8%) | 72 (29.8%) |  |
| Central nervous system tumor | 0 (0.0%) | 2 (0.8%) |  |
| Other | 4 (4.3%) | 5 (2.1%) |  |
| **Donor terminal creatinine (mg/dl), median (IQR)** | 0.78 (0.61-1.05) | 0.84 (0.69-1.01) | 0.089 |
| **Donors diabetes (N (%))** |  |  | 0.95 |
| No | 156 (95.7%) | 345 (95.8%) |  |
| Yes | 7 (4.3%) | 15 (4.2%) |  |
| **Donors hypertension (N (%))** |  |  | 0.43 |
| No | 141 (87.6%) | 305 (85.0%) |  |
| Yes | 20 (12.4%) | 54 (15.0%) |  |
| **Donor malignancy (N (%))** |  |  | 0.66 |
| No | 160 (98.2%) | 357 (97.5%) |  |
| Yes | 3 (1.8%) | 9 (2.5%) |  |
| **Immunological characteristics** |  |  |  |
| **Number of HLA mismatches (HLA A,B and DR) (N (%))** |  |  | <0.001 |
| 0 | 17 (10.4%) | 0 (0.0%) |  |
| 1 | 10 (6.1%) | 1 (0.3%) |  |
| 2 | 26 (16.0%) | 3 (0.8%) |  |
| 3 | 51 (31.3%) | 46 (12.6%) |  |
| 4 | 38 (23.3%) | 85 (23.2%) |  |
| 5 | 16 (9.8%) | 153 (41.8%) |  |
| 6 | 5 (3.1%) | 78 (21.3%) |  |
| **HLA mismatches A (N (%))** |  |  | 0.006 |
| 0 | 10 (24%) | 7 (8%) |  |
| 1 | 19 (46%) | 33 (38%) |  |
| 2 | 12 (29%) | 48 (55%) |  |
| **HLA mismatches B (N (%))** |  |  | <0.001 |
| 0 | 5 (12%) | 2 (2%) |  |
| 1 | 18 (44%) | 17 (19%) |  |
| 2 | 18 (44%) | 69 (78%) |  |
| **HLA mismatches DR (N (%))** |  |  | <0.001 |
| 0 | 27 (32.1%) | 8 (4.4%) |  |
| 1 | 46 (54.8%) | 87 (48.3%) |  |
| 2 | 11 (13.1%) | 85 (47.2%) |  |
| **HLA mismatches DQ (N (%))** |  |  | <0.001 |
| 0 | 20 (49%) | 4 (5%) |  |
| 1 | 19 (46%) | 39 (45%) |  |
| 2 | 2 (5%) | 44 (51%) |  |
| **Current flow B cell (N (%))** |  |  | 0.61 |
| < 2 SD compatible | 80 (96.4%) | 175 (93.6%) |  |
| 2-3 SD compatible | 1 (1.2%) | 6 (3.2%) |  |
| weak positive | 1 (1.2%) | 1 (0.5%) |  |
| positive | 1 (1.2%) | 5 (2.7%) |  |
| **Current flow T cell (N (%))** |  |  | 0.29 |
| < 2 SD compatible | 80 (96.4%) | 178 (95.2%) |  |
| 2-3 SD compatible | 3 (3.6%) | 3 (1.6%) |  |
| weak positive | 0 (0.0%) | 1 (0.5%) |  |
| positive | 0 (0.0%) | 5 (2.7%) |  |
| **Induction treatment (N (%))** |  |  |  |
| No Induction | 0 (0%) | 0 (0%) | 1.000 |
| Basiliximab | 33 (20.2%) | 34 (9.3%) | <0.001 |
| Thymoglobulin | 127 (77.9%) | 325 (88.8%) | 0.001 |
| Steroid | 163 (100.0%) | 365 (99.7%) | 0.50 |
| Alemtuzumab | 1 (0.6%) | 5 (1.4%) | 0.45 |
| **Outcomes** |  |  |  |
| **Proportion of Antibody Mediated Rejection of the subset of patient underwent biopsy (N (%))** |  |  | 0.063 |
| No | 41 (85.4%) | 95 (72.0%) |  |
| Yes | 7 (14.6%) | 37 (28.0%) |  |
| **Proportion of rejection of the subset of patient underwent biopsy (N (%))** |  |  | 0.089 |
| No | 34 (70.8%) | 75 (56.8%) |  |
| Yes | 14 (29.2%) | 57 (43.2%) |  |
| **Proportion of T-cell Mediated Rejection of the subset of patient underwent biopsy (N (%))** |  |  | 0.28 |
| No | 35 (72.9%) | 85 (64.4%) |  |
| Yes | 13 (27.1%) | 47 (35.6%) |  |
| **Post-Transplant Donor Specific Antibodies (N (%))** |  |  | <0.001 |
| No | 139 (89.7%) | 265 (73.0%) |  |
| Yes | 16 (10.3%) | 98 (27.0%) |  |
| **Dd-cf-DNA elevation (N (%))** |  |  | 0.003 |
| No | 142 (87.1%) | 277 (75.7%) |  |
| Yes | 21 (12.9%) | 89 (24.3%) |  |
| **Primary outcome (N (%))** |  |  | <0.001 |
| No | 128 (78.5%) | 208 (56.8%) |  |
| Yes | 35 (21.5%) | 158 (43.2%) |  |
| **Delayed Graft Function (N (%))** |  |  | 0.84 |
| No | 152 (93.3%) | 343 (93.7%) |  |
| Yes | 11 (6.7%) | 23 (6.3%) |  |
| **Death (N (%))** |  |  | 0.71 |
| No | 157 (96.3%) | 350 (95.6%) |  |
| Yes | 6 (3.7%) | 16 (4.4%) |  |
| **Graft Loss (N (%))** |  |  | 0.89 |
| No | 161 (98.8%) | 362 (98.9%) |  |
| Yes | 2 (1.2%) | 4 (1.1%) |  |

Values are expressed as mean (standard deviation), median (interquartile range), or number (%). Continuous variables were compared via t-tests or Mann-Whitney U tests. Categorical variables were compared via Chi-square tests.

**Table S15:** Performance characteristics table of PIRCHE-T2 for donor-derived cell-free DNA

| Cutpoint | Sensitivity | Specificity | Overall % correctly classified | LR+ | LR- |
| --- | --- | --- | --- | --- | --- |
| ( >= 0 ) | 100.00% | 0.00% | 19.64% | 1 |  |
| ( >= 1 ) | 100.00% | 4.44% | 23.21% | 1.0465 | 0 |
| ( >= 8 ) | 100.00% | 4.76% | 23.47% | 1.05 | 0 |
| ( >= 10 ) | 100.00% | 5.71% | 24.23% | 1.0606 | 0 |
| ( >= 11 ) | 100.00% | 6.35% | 24.74% | 1.0678 | 0 |
| ( >= 12 ) | 100.00% | 6.67% | 25.00% | 1.0714 | 0 |
| ( >= 14 ) | 100.00% | 6.98% | 25.26% | 1.0751 | 0 |
| ( >= 15 ) | 100.00% | 7.30% | 25.51% | 1.0788 | 0 |
| ( >= 16 ) | 98.70% | 7.94% | 25.77% | 1.0721 | 0.1636 |
| ( >= 17 ) | 98.70% | 8.25% | 26.02% | 1.0758 | 0.1573 |
| ( >= 20 ) | 98.70% | 9.21% | 26.79% | 1.0871 | 0.1411 |
| ( >= 21 ) | 97.40% | 9.84% | 27.04% | 1.0803 | 0.2639 |
| ( >= 22 ) | 96.10% | 10.48% | 27.30% | 1.0735 | 0.3719 |
| ( >= 23 ) | 96.10% | 10.79% | 27.55% | 1.0773 | 0.361 |
| ( >= 26 ) | 93.51% | 10.79% | 27.04% | 1.0482 | 0.6016 |
| ( >= 28 ) | 93.51% | 11.11% | 27.30% | 1.0519 | 0.5844 |
| ( >= 30 ) | 93.51% | 11.43% | 27.55% | 1.0557 | 0.5682 |
| ( >= 31 ) | 93.51% | 12.06% | 28.06% | 1.0633 | 0.5383 |
| ( >= 31.71 ) | 93.51% | 13.02% | 28.83% | 1.075 | 0.4989 |
| ( >= 32 ) | 93.51% | 13.33% | 29.08% | 1.0789 | 0.487 |
| ( >= 33 ) | 92.21% | 15.24% | 30.36% | 1.0878 | 0.5114 |
| ( >= 34 ) | 92.21% | 16.19% | 31.12% | 1.1002 | 0.4813 |
| ( >= 35 ) | 92.21% | 16.51% | 31.38% | 1.1044 | 0.472 |
| ( >= 36 ) | 92.21% | 17.46% | 32.14% | 1.1171 | 0.4463 |
| ( >= 38 ) | 92.21% | 19.68% | 33.93% | 1.148 | 0.3959 |
| ( >= 39 ) | 92.21% | 21.59% | 35.46% | 1.1759 | 0.361 |
| ( >= 40 ) | 92.21% | 21.90% | 35.71% | 1.1807 | 0.3557 |
| ( >= 41 ) | 92.21% | 22.86% | 36.48% | 1.1953 | 0.3409 |
| ( >= 42 ) | 90.91% | 24.13% | 37.24% | 1.1982 | 0.3768 |
| ( >= 43 ) | 89.61% | 24.76% | 37.50% | 1.191 | 0.4196 |
| ( >= 44 ) | 89.61% | 25.08% | 37.76% | 1.1961 | 0.4143 |
| ( >= 45 ) | 89.61% | 25.40% | 38.01% | 1.2012 | 0.4091 |
| ( >= 45.35 ) | 89.61% | 27.30% | 39.54% | 1.2326 | 0.3805 |
| ( >= 46 ) | 89.61% | 27.62% | 39.80% | 1.238 | 0.3762 |
| ( >= 47 ) | 89.61% | 28.25% | 40.31% | 1.249 | 0.3677 |
| ( >= 48 ) | 89.61% | 29.21% | 41.07% | 1.2658 | 0.3557 |
| ( >= 48.19 ) | 89.61% | 29.84% | 41.58% | 1.2773 | 0.3482 |
| ( >= 49 ) | 89.61% | 30.16% | 41.84% | 1.2831 | 0.3445 |
| ( >= 50 ) | 87.01% | 30.79% | 41.84% | 1.2573 | 0.4217 |
| ( >= 51 ) | 85.71% | 31.43% | 42.09% | 1.25 | 0.4545 |
| ( >= 52 ) | 85.71% | 32.06% | 42.60% | 1.2617 | 0.4455 |
| ( >= 53 ) | 85.71% | 33.65% | 43.88% | 1.2919 | 0.4245 |
| ( >= 54 ) | 85.71% | 34.60% | 44.64% | 1.3107 | 0.4128 |
| ( >= 55 ) | 85.71% | 36.83% | 46.43% | 1.3568 | 0.3879 |
| ( >= 56 ) | 84.42% | 37.78% | 46.94% | 1.3567 | 0.4125 |
| ( >= 57 ) | 83.12% | 39.37% | 47.96% | 1.3708 | 0.4289 |
| ( >= 58 ) | 81.82% | 40.95% | 48.98% | 1.3856 | 0.444 |
| ( >= 59 ) | 79.22% | 42.54% | 49.74% | 1.3787 | 0.4885 |
| ( >= 60 ) | 79.22% | 43.49% | 50.51% | 1.4019 | 0.4778 |
| ( >= 61 ) | 77.92% | 44.76% | 51.28% | 1.4107 | 0.4932 |
| ( >= 62 ) | 77.92% | 45.08% | 51.53% | 1.4188 | 0.4898 |
| ( >= 63 ) | 77.92% | 46.03% | 52.30% | 1.4439 | 0.4796 |
| ( >= 64 ) | 76.62% | 46.03% | 52.04% | 1.4198 | 0.5078 |
| ( >= 65 ) | 75.32% | 47.30% | 52.81% | 1.4294 | 0.5217 |
| ( >= 66 ) | 72.73% | 47.30% | 52.30% | 1.3801 | 0.5766 |
| ( >= 67 ) | 70.13% | 49.21% | 53.32% | 1.3807 | 0.607 |
| ( >= 68 ) | 68.83% | 50.79% | 54.34% | 1.3988 | 0.6136 |
| ( >= 69 ) | 67.53% | 51.43% | 54.59% | 1.3904 | 0.6313 |
| ( >= 70 ) | 66.23% | 53.97% | 56.38% | 1.4389 | 0.6257 |
| ( >= 71 ) | 66.23% | 54.92% | 57.14% | 1.4693 | 0.6148 |
| ( >= 72 ) | 66.23% | 55.56% | 57.65% | 1.4903 | 0.6078 |
| ( >= 73 ) | 64.94% | 56.83% | 58.42% | 1.504 | 0.6171 |
| ( >= 73.8 ) | 63.64% | 57.46% | 58.67% | 1.4959 | 0.6328 |
| ( >= 74 ) | 63.64% | 57.78% | 58.93% | 1.5072 | 0.6294 |
| ( >= 75 ) | 59.74% | 58.41% | 58.67% | 1.4365 | 0.6892 |
| ( >= 76 ) | 57.14% | 59.05% | 58.67% | 1.3953 | 0.7258 |
| ( >= 77 ) | 55.84% | 59.68% | 58.93% | 1.3851 | 0.7398 |
| ( >= 78 ) | 54.55% | 61.27% | 59.95% | 1.4083 | 0.7419 |
| ( >= 79 ) | 50.65% | 64.76% | 61.99% | 1.4373 | 0.762 |
| ( >= 80 ) | 49.35% | 65.08% | 61.99% | 1.4132 | 0.7783 |
| ( >= 81 ) | 49.35% | 66.35% | 63.01% | 1.4666 | 0.7634 |
| ( >= 83 ) | 46.75% | 67.30% | 63.27% | 1.4298 | 0.7912 |
| ( >= 84 ) | 45.45% | 68.89% | 64.29% | 1.461 | 0.7918 |
| ( >= 84.08 ) | 42.86% | 69.52% | 64.29% | 1.4063 | 0.8219 |
| ( >= 85 ) | 41.56% | 69.52% | 64.03% | 1.3636 | 0.8406 |
| ( >= 86 ) | 41.56% | 69.84% | 64.29% | 1.378 | 0.8368 |
| ( >= 87 ) | 38.96% | 69.84% | 63.78% | 1.2919 | 0.874 |
| ( >= 87.81 ) | 38.96% | 70.79% | 64.54% | 1.334 | 0.8622 |
| ( >= 88 ) | 38.96% | 71.11% | 64.80% | 1.3487 | 0.8584 |
| ( >= 89 ) | 37.66% | 72.70% | 65.82% | 1.3795 | 0.8575 |
| ( >= 90 ) | 37.66% | 73.02% | 66.07% | 1.3957 | 0.8538 |
| ( >= 91 ) | 37.66% | 73.33% | 66.33% | 1.4123 | 0.8501 |
| ( >= 92 ) | 37.66% | 74.29% | 67.09% | 1.4646 | 0.8392 |
| ( >= 93 ) | 36.36% | 74.29% | 66.84% | 1.4141 | 0.8566 |
| ( >= 94 ) | 36.36% | 74.92% | 67.35% | 1.4499 | 0.8494 |
| ( >= 95 ) | 35.06% | 75.56% | 67.60% | 1.4345 | 0.8594 |
| ( >= 96 ) | 35.06% | 76.19% | 68.11% | 1.4727 | 0.8523 |
| ( >= 97 ) | 31.17% | 76.19% | 67.35% | 1.3091 | 0.9034 |
| ( >= 98 ) | 29.87% | 77.14% | 67.86% | 1.3068 | 0.9091 |
| ( >= 99 ) | 27.27% | 78.10% | 68.11% | 1.2451 | 0.9313 |
| ( >= 100 ) | 25.97% | 78.73% | 68.37% | 1.2212 | 0.9402 |
| ( >= 101 ) | 24.68% | 80.00% | 69.13% | 1.2338 | 0.9416 |
| ( >= 102 ) | 24.68% | 81.27% | 70.15% | 1.3174 | 0.9268 |
| ( >= 103 ) | 24.68% | 81.59% | 70.41% | 1.3401 | 0.9232 |
| ( >= 104 ) | 22.08% | 82.22% | 70.41% | 1.2419 | 0.9477 |
| ( >= 105 ) | 20.78% | 83.49% | 71.17% | 1.2587 | 0.9488 |
| ( >= 105.17 ) | 19.48% | 83.81% | 71.17% | 1.2032 | 0.9607 |
| ( >= 106 ) | 19.48% | 84.13% | 71.43% | 1.2273 | 0.9571 |
| ( >= 107 ) | 15.58% | 84.13% | 70.66% | 0.9818 | 1.0034 |
| ( >= 108 ) | 15.58% | 84.44% | 70.92% | 1.0019 | 0.9997 |
| ( >= 109 ) | 15.58% | 85.71% | 71.94% | 1.0909 | 0.9848 |
| ( >= 110 ) | 15.58% | 86.03% | 72.19% | 1.1157 | 0.9812 |
| ( >= 112 ) | 15.58% | 86.35% | 72.45% | 1.1416 | 0.9776 |
| ( >= 113 ) | 14.29% | 86.98% | 72.70% | 1.0976 | 0.9854 |
| ( >= 114 ) | 14.29% | 87.62% | 73.21% | 1.1538 | 0.9783 |
| ( >= 115 ) | 14.29% | 87.94% | 73.47% | 1.1842 | 0.9747 |
| ( >= 116 ) | 12.99% | 87.94% | 73.21% | 1.0766 | 0.9895 |
| ( >= 118 ) | 12.99% | 88.57% | 73.72% | 1.1364 | 0.9824 |
| ( >= 119 ) | 11.69% | 89.84% | 74.49% | 1.1506 | 0.983 |
| ( >= 120 ) | 11.69% | 90.79% | 75.26% | 1.2696 | 0.9727 |
| ( >= 121 ) | 11.69% | 91.43% | 75.77% | 1.3636 | 0.9659 |
| ( >= 123 ) | 11.69% | 91.75% | 76.02% | 1.4161 | 0.9626 |
| ( >= 124 ) | 10.39% | 92.06% | 76.02% | 1.3091 | 0.9734 |
| ( >= 125 ) | 9.09% | 92.06% | 75.77% | 1.1455 | 0.9875 |
| ( >= 126 ) | 7.79% | 92.06% | 75.51% | 0.9818 | 1.0016 |
| ( >= 127 ) | 5.19% | 93.33% | 76.02% | 0.7792 | 1.0158 |
| ( >= 128 ) | 5.19% | 93.65% | 76.28% | 0.8182 | 1.0123 |
| ( >= 131 ) | 5.19% | 93.97% | 76.53% | 0.8612 | 1.0089 |
| ( >= 133 ) | 5.19% | 94.29% | 76.79% | 0.9091 | 1.0055 |
| ( >= 134 ) | 5.19% | 95.24% | 77.55% | 1.0909 | 0.9955 |
| ( >= 136 ) | 5.19% | 95.87% | 78.06% | 1.2587 | 0.9889 |
| ( >= 137 ) | 5.19% | 96.51% | 78.57% | 1.4876 | 0.9824 |
| ( >= 139 ) | 5.19% | 96.83% | 78.83% | 1.6364 | 0.9791 |
| ( >= 140 ) | 3.90% | 97.14% | 78.83% | 1.3636 | 0.9893 |
| ( >= 144 ) | 2.60% | 97.14% | 78.57% | 0.9091 | 1.0027 |
| ( >= 145 ) | 2.60% | 97.46% | 78.83% | 1.0227 | 0.9994 |
| ( >= 146 ) | 2.60% | 98.10% | 79.34% | 1.3636 | 0.9929 |
| ( >= 148 ) | 2.60% | 98.41% | 79.59% | 1.6364 | 0.9897 |
| ( >= 152 ) | 2.60% | 98.73% | 79.85% | 2.0455 | 0.9866 |
| ( >= 153 ) | 2.60% | 99.05% | 80.10% | 2.7273 | 0.9834 |
| ( >= 164 ) | 2.60% | 99.37% | 80.36% | 4.0909 | 0.9802 |
| ( >= 167 ) | 1.30% | 99.37% | 80.10% | 2.0455 | 0.9933 |
| ( >= 169 ) | 1.30% | 99.68% | 80.36% | 4.0909 | 0.9902 |
| ( >= 172 ) | 1.30% | 100.00% | 80.61% |  | 0.987 |
| ( > 172 ) | 0.00% | 100.00% | 80.36% |  | 1 |

LR: Likelihood ratio

**Table S16:** Performance characteristics table of PIRCHE-B for donor-derived cell-free DNA

| Cutpoint | Sensitivity | Specificity | Overall % correctly classified | LR+ | LR- |
| --- | --- | --- | --- | --- | --- |
| ( >= 0 ) | 100.00% | 0.00% | 20.79% | 1 |  |
| ( >= 1 ) | 100.00% | 4.06% | 24.01% | 1.0423 | 0 |
| ( >= 2 ) | 100.00% | 5.01% | 24.76% | 1.0528 | 0 |
| ( >= 3 ) | 99.09% | 6.21% | 25.52% | 1.0565 | 0.1465 |
| ( >= 4 ) | 98.18% | 8.35% | 27.03% | 1.0713 | 0.2177 |
| ( >= 5 ) | 97.27% | 10.50% | 28.54% | 1.0869 | 0.2597 |
| ( >= 6 ) | 95.45% | 13.37% | 30.43% | 1.1018 | 0.3401 |
| ( >= 6.69 ) | 93.64% | 17.90% | 33.65% | 1.1405 | 0.3555 |
| ( >= 7 ) | 93.64% | 18.14% | 33.84% | 1.1438 | 0.3508 |
| ( >= 8 ) | 90.00% | 22.91% | 36.86% | 1.1675 | 0.4365 |
| ( >= 9 ) | 85.45% | 26.73% | 38.94% | 1.1663 | 0.5442 |
| ( >= 9.02 ) | 80.91% | 33.41% | 43.29% | 1.2151 | 0.5714 |
| ( >= 9.51 ) | 80.91% | 33.65% | 43.48% | 1.2195 | 0.5673 |
| ( >= 10 ) | 80.91% | 33.89% | 43.67% | 1.2239 | 0.5633 |
| ( >= 10.71 ) | 76.36% | 38.19% | 46.12% | 1.2354 | 0.619 |
| ( >= 11 ) | 76.36% | 38.42% | 46.31% | 1.2402 | 0.6151 |
| ( >= 12 ) | 70.00% | 43.44% | 48.96% | 1.2376 | 0.6907 |
| ( >= 13 ) | 62.73% | 48.21% | 51.23% | 1.2112 | 0.7731 |
| ( >= 13.17 ) | 55.45% | 52.98% | 53.50% | 1.1795 | 0.8407 |
| ( >= 14 ) | 55.45% | 53.22% | 53.69% | 1.1855 | 0.837 |
| ( >= 15 ) | 50.00% | 58.47% | 56.71% | 1.204 | 0.8551 |
| ( >= 16 ) | 44.55% | 63.01% | 59.17% | 1.2042 | 0.8801 |
| ( >= 17 ) | 39.09% | 67.06% | 61.25% | 1.1869 | 0.9082 |
| ( >= 17.24 ) | 34.55% | 72.32% | 64.46% | 1.2478 | 0.9051 |
| ( >= 18 ) | 33.64% | 72.32% | 64.27% | 1.215 | 0.9177 |
| ( >= 19 ) | 30.91% | 75.89% | 66.54% | 1.2823 | 0.9103 |
| ( >= 20 ) | 28.18% | 79.00% | 68.43% | 1.3418 | 0.9091 |
| ( >= 21 ) | 20.00% | 82.10% | 69.19% | 1.1173 | 0.9744 |
| ( >= 22 ) | 15.45% | 85.68% | 71.08% | 1.0792 | 0.9868 |
| ( >= 23 ) | 13.64% | 88.54% | 72.97% | 1.1903 | 0.9754 |
| ( >= 24 ) | 10.91% | 90.93% | 74.29% | 1.2029 | 0.9798 |
| ( >= 25 ) | 9.09% | 93.32% | 75.80% | 1.3604 | 0.9742 |
| ( >= 26 ) | 8.18% | 95.47% | 77.32% | 1.8043 | 0.9618 |
| ( >= 27 ) | 4.55% | 97.14% | 77.88% | 1.5871 | 0.9827 |
| ( >= 28 ) | 4.55% | 97.85% | 78.45% | 2.1162 | 0.9755 |
| ( >= 30 ) | 1.82% | 98.57% | 78.45% | 1.2697 | 0.9961 |
| ( >= 31 ) | 0.91% | 98.57% | 78.26% | 0.6348 | 1.0053 |
| ( >= 32 ) | 0.91% | 99.05% | 78.64% | 0.9523 | 1.0005 |
| ( >= 33 ) | 0.91% | 99.28% | 78.83% | 1.2697 | 0.9981 |
| ( >= 34 ) | 0.91% | 99.76% | 79.21% | 3.8091 | 0.9933 |
| ( >= 54 ) | 0.00% | 99.76% | 79.02% | 0 | 1.0024 |
| ( > 54 ) | 0.00% | 100.00% | 79.21% |  | 1 |

LR: Likelihood ratio

**Table S17:** Performance characteristics table of six-locus PIRCHE-T2 for primary outcome

| Cutpoint | Sensitivity | Specificity | Overall % correctly classified | LR+ | LR- |
| --- | --- | --- | --- | --- | --- |
| ( >= 0 ) | 100.00% | 0.00% | 34.13% | 1 |  |
| ( >= 1 ) | 99.42% | 5.76% | 37.72% | 1.0549 | 0.1016 |
| ( >= 5 ) | 99.42% | 6.06% | 37.92% | 1.0583 | 0.0965 |
| ( >= 7 ) | 99.42% | 6.36% | 38.12% | 1.0617 | 0.0919 |
| ( >= 8 ) | 98.83% | 6.36% | 37.92% | 1.0555 | 0.1838 |
| ( >= 9 ) | 98.83% | 7.27% | 38.52% | 1.0658 | 0.1608 |
| ( >= 10 ) | 98.25% | 7.27% | 38.32% | 1.0595 | 0.2412 |
| ( >= 11 ) | 97.66% | 7.58% | 38.32% | 1.0567 | 0.3088 |
| ( >= 12 ) | 97.66% | 7.88% | 38.52% | 1.0601 | 0.2969 |
| ( >= 14 ) | 97.66% | 8.48% | 38.92% | 1.0672 | 0.2757 |
| ( >= 15 ) | 97.66% | 8.79% | 39.12% | 1.0707 | 0.2662 |
| ( >= 16 ) | 97.08% | 9.39% | 39.32% | 1.0714 | 0.3113 |
| ( >= 17 ) | 97.08% | 9.70% | 39.52% | 1.075 | 0.3015 |
| ( >= 20 ) | 97.08% | 10.91% | 40.32% | 1.0896 | 0.268 |
| ( >= 21 ) | 96.49% | 11.52% | 40.52% | 1.0905 | 0.3047 |
| ( >= 22 ) | 95.91% | 12.42% | 40.92% | 1.0951 | 0.3295 |
| ( >= 23 ) | 95.91% | 12.73% | 41.12% | 1.0989 | 0.3216 |
| ( >= 24.96 ) | 94.74% | 12.73% | 40.72% | 1.0855 | 0.4135 |
| ( >= 25 ) | 94.15% | 12.73% | 40.52% | 1.0788 | 0.4595 |
| ( >= 26 ) | 93.57% | 12.73% | 40.32% | 1.0721 | 0.5054 |
| ( >= 28 ) | 93.57% | 13.03% | 40.52% | 1.0759 | 0.4937 |
| ( >= 29 ) | 93.57% | 13.64% | 40.92% | 1.0834 | 0.4717 |
| ( >= 30 ) | 92.98% | 13.64% | 40.72% | 1.0766 | 0.5146 |
| ( >= 31 ) | 92.40% | 14.55% | 41.12% | 1.0812 | 0.5227 |
| ( >= 31.71 ) | 92.40% | 15.45% | 41.72% | 1.0929 | 0.4919 |
| ( >= 32 ) | 92.40% | 15.76% | 41.92% | 1.0968 | 0.4825 |
| ( >= 33 ) | 91.23% | 17.27% | 42.51% | 1.1028 | 0.5078 |
| ( >= 34 ) | 91.23% | 18.18% | 43.11% | 1.115 | 0.4825 |
| ( >= 35 ) | 90.64% | 18.18% | 42.91% | 1.1079 | 0.5146 |
| ( >= 35.22 ) | 89.47% | 18.79% | 42.91% | 1.1017 | 0.5603 |
| ( >= 36 ) | 89.47% | 19.09% | 43.11% | 1.1059 | 0.5514 |
| ( >= 37 ) | 89.47% | 21.52% | 44.71% | 1.14 | 0.4893 |
| ( >= 38 ) | 89.47% | 21.82% | 44.91% | 1.1444 | 0.4825 |
| ( >= 39 ) | 88.89% | 23.33% | 45.71% | 1.1594 | 0.4762 |
| ( >= 40 ) | 88.30% | 23.64% | 45.71% | 1.1564 | 0.4948 |
| ( >= 41 ) | 87.72% | 24.24% | 45.91% | 1.1579 | 0.5066 |
| ( >= 42 ) | 85.96% | 25.15% | 45.91% | 1.1485 | 0.558 |
| ( >= 43 ) | 85.38% | 25.76% | 46.11% | 1.15 | 0.5676 |
| ( >= 44 ) | 84.80% | 26.06% | 46.11% | 1.1468 | 0.5834 |
| ( >= 45 ) | 83.04% | 26.67% | 45.91% | 1.1324 | 0.636 |
| ( >= 45.35 ) | 82.46% | 28.79% | 47.11% | 1.1579 | 0.6094 |
| ( >= 45.78 ) | 82.46% | 29.09% | 47.31% | 1.1628 | 0.6031 |
| ( >= 46 ) | 81.87% | 29.09% | 47.11% | 1.1546 | 0.6232 |
| ( >= 47 ) | 81.87% | 30.00% | 47.70% | 1.1696 | 0.6043 |
| ( >= 48 ) | 80.70% | 30.30% | 47.50% | 1.1579 | 0.6368 |
| ( >= 48.19 ) | 79.53% | 30.61% | 47.31% | 1.1461 | 0.6688 |
| ( >= 49 ) | 79.53% | 30.91% | 47.50% | 1.1511 | 0.6622 |
| ( >= 50 ) | 78.36% | 31.82% | 47.70% | 1.1493 | 0.68 |
| ( >= 51 ) | 77.78% | 32.42% | 47.90% | 1.151 | 0.6854 |
| ( >= 52 ) | 77.78% | 33.03% | 48.30% | 1.1614 | 0.6728 |
| ( >= 53 ) | 76.61% | 34.24% | 48.70% | 1.165 | 0.6831 |
| ( >= 54 ) | 76.61% | 35.15% | 49.30% | 1.1813 | 0.6655 |
| ( >= 55 ) | 76.61% | 37.27% | 50.70% | 1.2213 | 0.6276 |
| ( >= 56 ) | 75.44% | 38.48% | 51.10% | 1.2263 | 0.6382 |
| ( >= 57 ) | 74.27% | 39.70% | 51.50% | 1.2316 | 0.6482 |
| ( >= 58 ) | 73.10% | 40.91% | 51.90% | 1.2371 | 0.6576 |
| ( >= 59 ) | 71.93% | 42.73% | 52.69% | 1.2559 | 0.657 |
| ( >= 60 ) | 71.35% | 43.64% | 53.09% | 1.2658 | 0.6567 |
| ( >= 60.23 ) | 70.18% | 44.55% | 53.29% | 1.2655 | 0.6695 |
| ( >= 61 ) | 70.18% | 44.85% | 53.49% | 1.2724 | 0.665 |
| ( >= 62 ) | 70.18% | 45.15% | 53.69% | 1.2794 | 0.6605 |
| ( >= 63 ) | 70.18% | 46.36% | 54.49% | 1.3084 | 0.6433 |
| ( >= 64 ) | 69.01% | 46.67% | 54.29% | 1.2939 | 0.6642 |
| ( >= 65 ) | 67.25% | 47.88% | 54.49% | 1.2903 | 0.684 |
| ( >= 66 ) | 66.08% | 48.79% | 54.69% | 1.2904 | 0.6952 |
| ( >= 67 ) | 62.57% | 50.30% | 54.49% | 1.2591 | 0.744 |
| ( >= 68 ) | 60.82% | 51.82% | 54.89% | 1.2623 | 0.7561 |
| ( >= 69 ) | 59.65% | 52.73% | 55.09% | 1.2618 | 0.7653 |
| ( >= 70 ) | 57.89% | 55.15% | 56.09% | 1.2909 | 0.7634 |
| ( >= 71 ) | 57.31% | 56.36% | 56.69% | 1.3134 | 0.7574 |
| ( >= 72 ) | 57.31% | 56.97% | 57.09% | 1.3319 | 0.7493 |
| ( >= 73 ) | 55.56% | 57.58% | 56.89% | 1.3095 | 0.7719 |
| ( >= 73.8 ) | 54.97% | 58.18% | 57.09% | 1.3145 | 0.7739 |
| ( >= 74 ) | 54.97% | 58.48% | 57.29% | 1.3241 | 0.7699 |
| ( >= 75 ) | 52.63% | 58.79% | 56.69% | 1.2771 | 0.8058 |
| ( >= 76 ) | 51.46% | 60.30% | 57.29% | 1.2964 | 0.8049 |
| ( >= 77 ) | 50.29% | 60.61% | 57.09% | 1.2767 | 0.8202 |
| ( >= 78 ) | 49.71% | 63.03% | 58.48% | 1.3445 | 0.7979 |
| ( >= 79 ) | 46.78% | 66.06% | 59.48% | 1.3784 | 0.8056 |
| ( >= 79.79 ) | 46.20% | 66.67% | 59.68% | 1.386 | 0.807 |
| ( >= 80 ) | 46.20% | 66.97% | 59.88% | 1.3987 | 0.8034 |
| ( >= 81 ) | 45.03% | 68.18% | 60.28% | 1.4152 | 0.8062 |
| ( >= 82 ) | 43.27% | 69.09% | 60.28% | 1.4001 | 0.821 |
| ( >= 83 ) | 42.11% | 69.39% | 60.08% | 1.3757 | 0.8343 |
| ( >= 84 ) | 40.35% | 70.30% | 60.08% | 1.3588 | 0.8485 |
| ( >= 84.08 ) | 38.60% | 70.91% | 59.88% | 1.3268 | 0.8659 |
| ( >= 85 ) | 38.01% | 70.91% | 59.68% | 1.3067 | 0.8742 |
| ( >= 86 ) | 37.43% | 71.21% | 59.68% | 1.3001 | 0.8787 |
| ( >= 87 ) | 35.67% | 71.21% | 59.08% | 1.2392 | 0.9033 |
| ( >= 87.81 ) | 35.67% | 72.12% | 59.68% | 1.2796 | 0.8919 |
| ( >= 88 ) | 35.67% | 72.42% | 59.88% | 1.2936 | 0.8882 |
| ( >= 89 ) | 33.92% | 73.94% | 60.28% | 1.3015 | 0.8937 |
| ( >= 90 ) | 33.92% | 74.24% | 60.48% | 1.3168 | 0.8901 |
| ( >= 91 ) | 33.33% | 74.55% | 60.48% | 1.3095 | 0.8943 |
| ( >= 91.38 ) | 32.75% | 75.45% | 60.88% | 1.3342 | 0.8913 |
| ( >= 92 ) | 32.75% | 75.76% | 61.08% | 1.3509 | 0.8877 |
| ( >= 93 ) | 31.58% | 75.76% | 60.68% | 1.3026 | 0.9032 |
| ( >= 94 ) | 31.58% | 76.67% | 61.28% | 1.3534 | 0.8924 |
| ( >= 95 ) | 30.99% | 77.27% | 61.48% | 1.3637 | 0.893 |
| ( >= 96 ) | 30.41% | 77.58% | 61.48% | 1.3561 | 0.8971 |
| ( >= 97 ) | 28.65% | 77.58% | 60.88% | 1.2779 | 0.9197 |
| ( >= 98 ) | 28.07% | 78.48% | 61.28% | 1.3047 | 0.9165 |
| ( >= 99 ) | 26.32% | 79.39% | 61.28% | 1.2771 | 0.9281 |
| ( >= 100 ) | 25.15% | 80.00% | 61.28% | 1.2573 | 0.9357 |
| ( >= 101 ) | 23.98% | 81.21% | 61.68% | 1.2762 | 0.9361 |
| ( >= 102 ) | 23.98% | 82.42% | 62.48% | 1.3642 | 0.9223 |
| ( >= 103 ) | 23.98% | 82.73% | 62.67% | 1.3881 | 0.919 |
| ( >= 104 ) | 22.22% | 83.33% | 62.48% | 1.3333 | 0.9333 |
| ( >= 105 ) | 21.05% | 84.85% | 63.07% | 1.3895 | 0.9305 |
| ( >= 105.17 ) | 19.88% | 84.85% | 62.67% | 1.3123 | 0.9442 |
| ( >= 106 ) | 19.30% | 84.85% | 62.48% | 1.2737 | 0.9511 |
| ( >= 107 ) | 17.54% | 85.15% | 62.08% | 1.1815 | 0.9683 |
| ( >= 108 ) | 17.54% | 85.45% | 62.28% | 1.2061 | 0.9649 |
| ( >= 109 ) | 16.37% | 86.36% | 62.48% | 1.2008 | 0.9683 |
| ( >= 110 ) | 16.37% | 86.67% | 62.67% | 1.2281 | 0.9649 |
| ( >= 111 ) | 15.20% | 86.67% | 62.28% | 1.1404 | 0.9784 |
| ( >= 112 ) | 14.62% | 86.67% | 62.08% | 1.0965 | 0.9852 |
| ( >= 113 ) | 14.04% | 87.27% | 62.28% | 1.1028 | 0.985 |
| ( >= 113.07 ) | 14.04% | 87.88% | 62.67% | 1.1579 | 0.9782 |
| ( >= 114 ) | 13.45% | 87.88% | 62.48% | 1.1096 | 0.9849 |
| ( >= 115 ) | 12.87% | 88.18% | 62.48% | 1.0886 | 0.9881 |
| ( >= 116 ) | 12.28% | 88.79% | 62.67% | 1.0953 | 0.988 |
| ( >= 117 ) | 11.70% | 89.39% | 62.87% | 1.1028 | 0.9878 |
| ( >= 118 ) | 11.70% | 89.70% | 63.07% | 1.1352 | 0.9845 |
| ( >= 119 ) | 11.11% | 90.91% | 63.67% | 1.2222 | 0.9778 |
| ( >= 120 ) | 9.94% | 91.21% | 63.47% | 1.1313 | 0.9874 |
| ( >= 121 ) | 9.94% | 92.12% | 64.07% | 1.2618 | 0.9776 |
| ( >= 122 ) | 9.94% | 92.42% | 64.27% | 1.3123 | 0.9744 |
| ( >= 123 ) | 9.36% | 92.42% | 64.07% | 1.2351 | 0.9807 |
| ( >= 124 ) | 8.77% | 92.73% | 64.07% | 1.2061 | 0.9838 |
| ( >= 125 ) | 8.19% | 92.73% | 63.87% | 1.1257 | 0.9901 |
| ( >= 126 ) | 7.60% | 92.73% | 63.67% | 1.0453 | 0.9964 |
| ( >= 127 ) | 5.85% | 93.94% | 63.87% | 0.9649 | 1.0023 |
| ( >= 128 ) | 5.85% | 94.24% | 64.07% | 1.0157 | 0.999 |
| ( >= 131 ) | 5.85% | 94.55% | 64.27% | 1.0721 | 0.9958 |
| ( >= 133 ) | 5.85% | 94.85% | 64.47% | 1.1352 | 0.9927 |
| ( >= 134 ) | 5.26% | 95.76% | 64.87% | 1.2406 | 0.9893 |
| ( >= 136 ) | 4.68% | 96.06% | 64.87% | 1.1876 | 0.9923 |
| ( >= 137 ) | 4.68% | 96.67% | 65.27% | 1.4035 | 0.9861 |
| ( >= 139 ) | 4.68% | 97.27% | 65.67% | 1.7154 | 0.9799 |
| ( >= 140 ) | 3.51% | 97.27% | 65.27% | 1.2866 | 0.992 |
| ( >= 144 ) | 2.92% | 97.27% | 65.07% | 1.0721 | 0.998 |
| ( >= 145 ) | 2.34% | 97.27% | 64.87% | 0.8577 | 1.004 |
| ( >= 146 ) | 2.34% | 97.88% | 65.27% | 1.1028 | 0.9978 |
| ( >= 148 ) | 2.34% | 98.18% | 65.47% | 1.2866 | 0.9947 |
| ( >= 152 ) | 1.75% | 98.18% | 65.27% | 0.9649 | 1.0006 |
| ( >= 153 ) | 1.75% | 98.48% | 65.47% | 1.1579 | 0.9976 |
| ( >= 162 ) | 1.75% | 98.79% | 65.67% | 1.4474 | 0.9945 |
| ( >= 163 ) | 1.75% | 99.09% | 65.87% | 1.9298 | 0.9915 |
| ( >= 164 ) | 1.17% | 99.09% | 65.67% | 1.2866 | 0.9974 |
| ( >= 167 ) | 0.58% | 99.39% | 65.67% | 0.9649 | 1.0002 |
| ( >= 169 ) | 0.58% | 99.70% | 65.87% | 1.9298 | 0.9972 |
| ( >= 172 ) | 0.58% | 100.00% | 66.07% |  | 0.9942 |
| ( > 172 ) | 0.00% | 100.00% | 65.87% |  | 1 |

LR: Likelihood ratio

**Table S18:** Performance characteristics table of six-locus PIRCHE-T2 for DSA

| Cutpoint | Sensitivity | Specificity | Overall % correctly classified | LR+ | LR- |
| --- | --- | --- | --- | --- | --- |
| ( >= 0 ) | 100.00% | 0.00% | 22.15% | 1 |  |
| ( >= 1 ) | 99.08% | 3.13% | 24.39% | 1.0229 | 0.2928 |
| ( >= 5 ) | 99.08% | 3.39% | 24.59% | 1.0256 | 0.2703 |
| ( >= 7 ) | 99.08% | 3.66% | 24.80% | 1.0284 | 0.251 |
| ( >= 8 ) | 98.17% | 3.66% | 24.59% | 1.0189 | 0.502 |
| ( >= 9 ) | 98.17% | 4.44% | 25.20% | 1.0272 | 0.4134 |
| ( >= 10 ) | 97.25% | 4.44% | 25.00% | 1.0176 | 0.6201 |
| ( >= 11 ) | 96.33% | 4.70% | 25.00% | 1.0108 | 0.7808 |
| ( >= 12 ) | 96.33% | 4.96% | 25.20% | 1.0136 | 0.7397 |
| ( >= 14 ) | 96.33% | 5.48% | 25.61% | 1.0192 | 0.6693 |
| ( >= 15 ) | 96.33% | 5.74% | 25.81% | 1.022 | 0.6389 |
| ( >= 16 ) | 96.33% | 6.53% | 26.42% | 1.0306 | 0.5622 |
| ( >= 17 ) | 96.33% | 6.79% | 26.63% | 1.0335 | 0.5406 |
| ( >= 20 ) | 96.33% | 7.83% | 27.44% | 1.0452 | 0.4685 |
| ( >= 21 ) | 96.33% | 8.62% | 28.05% | 1.0541 | 0.4259 |
| ( >= 22 ) | 95.41% | 9.40% | 28.46% | 1.0531 | 0.488 |
| ( >= 23 ) | 95.41% | 9.66% | 28.66% | 1.0562 | 0.4748 |
| ( >= 24.96 ) | 95.41% | 10.18% | 29.07% | 1.0623 | 0.4505 |
| ( >= 25 ) | 94.50% | 10.18% | 28.86% | 1.0521 | 0.5406 |
| ( >= 26 ) | 93.58% | 10.18% | 28.66% | 1.0419 | 0.6307 |
| ( >= 28 ) | 93.58% | 10.44% | 28.86% | 1.0449 | 0.6149 |
| ( >= 29 ) | 93.58% | 10.97% | 29.27% | 1.051 | 0.5856 |
| ( >= 30 ) | 92.66% | 10.97% | 29.07% | 1.0407 | 0.6693 |
| ( >= 31 ) | 91.74% | 11.75% | 29.47% | 1.0396 | 0.7028 |
| ( >= 31.71 ) | 91.74% | 12.53% | 30.08% | 1.0489 | 0.6588 |
| ( >= 32 ) | 91.74% | 12.79% | 30.28% | 1.052 | 0.6454 |
| ( >= 33 ) | 90.83% | 14.36% | 31.30% | 1.0606 | 0.6389 |
| ( >= 34 ) | 90.83% | 15.14% | 31.91% | 1.0703 | 0.6058 |
| ( >= 35 ) | 89.91% | 15.14% | 31.71% | 1.0595 | 0.6664 |
| ( >= 35.22 ) | 88.07% | 15.67% | 31.71% | 1.0443 | 0.7613 |
| ( >= 36 ) | 88.07% | 15.93% | 31.91% | 1.0476 | 0.7488 |
| ( >= 37 ) | 88.07% | 18.02% | 33.54% | 1.0743 | 0.662 |
| ( >= 38 ) | 88.07% | 18.28% | 33.74% | 1.0777 | 0.6526 |
| ( >= 39 ) | 87.16% | 19.58% | 34.55% | 1.0838 | 0.6559 |
| ( >= 40 ) | 87.16% | 20.10% | 34.96% | 1.0909 | 0.6389 |
| ( >= 41 ) | 87.16% | 20.89% | 35.57% | 1.1017 | 0.6149 |
| ( >= 42 ) | 84.40% | 21.67% | 35.57% | 1.0776 | 0.7197 |
| ( >= 43 ) | 84.40% | 22.45% | 36.18% | 1.0884 | 0.6946 |
| ( >= 44 ) | 83.49% | 22.72% | 36.18% | 1.0802 | 0.727 |
| ( >= 45 ) | 82.57% | 23.76% | 36.79% | 1.083 | 0.7336 |
| ( >= 45.35 ) | 81.65% | 25.59% | 38.01% | 1.0973 | 0.7171 |
| ( >= 45.78 ) | 81.65% | 25.85% | 38.21% | 1.1011 | 0.7099 |
| ( >= 46 ) | 80.73% | 25.85% | 38.01% | 1.0888 | 0.7453 |
| ( >= 47 ) | 80.73% | 26.63% | 38.62% | 1.1004 | 0.7234 |
| ( >= 48 ) | 78.90% | 26.63% | 38.21% | 1.0754 | 0.7923 |
| ( >= 48.19 ) | 77.06% | 26.89% | 38.01% | 1.0541 | 0.8529 |
| ( >= 49 ) | 77.06% | 27.15% | 38.21% | 1.0579 | 0.8447 |
| ( >= 50 ) | 76.15% | 28.20% | 38.82% | 1.0605 | 0.8459 |
| ( >= 51 ) | 75.23% | 28.72% | 39.02% | 1.0554 | 0.8625 |
| ( >= 52 ) | 75.23% | 29.24% | 39.43% | 1.0632 | 0.8471 |
| ( >= 53 ) | 73.39% | 30.29% | 39.84% | 1.0528 | 0.8784 |
| ( >= 54 ) | 73.39% | 31.07% | 40.45% | 1.0648 | 0.8563 |
| ( >= 55 ) | 73.39% | 32.90% | 41.87% | 1.0938 | 0.8087 |
| ( >= 56 ) | 73.39% | 34.46% | 43.09% | 1.1199 | 0.772 |
| ( >= 57 ) | 71.56% | 35.51% | 43.50% | 1.1096 | 0.8009 |
| ( >= 58 ) | 70.64% | 36.81% | 44.31% | 1.118 | 0.7974 |
| ( >= 59 ) | 70.64% | 38.90% | 45.93% | 1.1562 | 0.7546 |
| ( >= 60 ) | 69.72% | 39.69% | 46.34% | 1.156 | 0.7629 |
| ( >= 60.23 ) | 67.89% | 40.47% | 46.54% | 1.1404 | 0.7934 |
| ( >= 61 ) | 67.89% | 40.73% | 46.75% | 1.1455 | 0.7883 |
| ( >= 62 ) | 67.89% | 40.99% | 46.95% | 1.1505 | 0.7833 |
| ( >= 63 ) | 67.89% | 42.04% | 47.76% | 1.1713 | 0.7639 |
| ( >= 64 ) | 66.97% | 42.56% | 47.97% | 1.1659 | 0.776 |
| ( >= 65 ) | 66.06% | 44.13% | 48.98% | 1.1822 | 0.7693 |
| ( >= 66 ) | 65.14% | 45.17% | 49.59% | 1.188 | 0.7718 |
| ( >= 67 ) | 62.39% | 47.26% | 50.61% | 1.1829 | 0.7959 |
| ( >= 68 ) | 59.63% | 48.56% | 51.02% | 1.1594 | 0.8312 |
| ( >= 69 ) | 59.63% | 49.87% | 52.03% | 1.1896 | 0.8095 |
| ( >= 70 ) | 59.63% | 52.74% | 54.27% | 1.2618 | 0.7654 |
| ( >= 71 ) | 58.72% | 53.79% | 54.88% | 1.2705 | 0.7676 |
| ( >= 72 ) | 58.72% | 54.31% | 55.28% | 1.285 | 0.7602 |
| ( >= 73 ) | 56.88% | 55.09% | 55.49% | 1.2666 | 0.7827 |
| ( >= 73.8 ) | 55.96% | 55.61% | 55.69% | 1.2608 | 0.7918 |
| ( >= 74 ) | 55.96% | 55.87% | 55.89% | 1.2683 | 0.7881 |
| ( >= 75 ) | 54.13% | 56.66% | 56.10% | 1.2489 | 0.8096 |
| ( >= 76 ) | 53.21% | 58.22% | 57.11% | 1.2737 | 0.8036 |
| ( >= 77 ) | 52.29% | 58.75% | 57.32% | 1.2676 | 0.8121 |
| ( >= 78 ) | 52.29% | 61.10% | 59.15% | 1.3442 | 0.7808 |
| ( >= 79 ) | 47.71% | 63.71% | 60.16% | 1.3145 | 0.8208 |
| ( >= 79.79 ) | 47.71% | 64.49% | 60.77% | 1.3435 | 0.8109 |
| ( >= 80 ) | 47.71% | 64.75% | 60.98% | 1.3534 | 0.8076 |
| ( >= 81 ) | 45.87% | 65.80% | 61.38% | 1.3411 | 0.8227 |
| ( >= 82 ) | 44.95% | 67.10% | 62.20% | 1.3665 | 0.8203 |
| ( >= 83 ) | 44.04% | 67.62% | 62.40% | 1.3602 | 0.8276 |
| ( >= 84 ) | 42.20% | 68.67% | 62.80% | 1.3469 | 0.8417 |
| ( >= 84.08 ) | 40.37% | 69.45% | 63.01% | 1.3214 | 0.8586 |
| ( >= 85 ) | 39.45% | 69.45% | 62.80% | 1.2914 | 0.8718 |
| ( >= 86 ) | 38.53% | 69.71% | 62.80% | 1.2722 | 0.8817 |
| ( >= 87 ) | 37.61% | 70.23% | 63.01% | 1.2637 | 0.8882 |
| ( >= 87.81 ) | 37.61% | 71.02% | 63.62% | 1.2979 | 0.8784 |
| ( >= 88 ) | 37.61% | 71.28% | 63.82% | 1.3097 | 0.8752 |
| ( >= 89 ) | 35.78% | 72.85% | 64.63% | 1.3177 | 0.8816 |
| ( >= 90 ) | 35.78% | 73.11% | 64.84% | 1.3305 | 0.8784 |
| ( >= 91 ) | 34.86% | 73.37% | 64.84% | 1.309 | 0.8878 |
| ( >= 91.38 ) | 34.86% | 74.41% | 65.65% | 1.3625 | 0.8754 |
| ( >= 92 ) | 34.86% | 74.67% | 65.85% | 1.3765 | 0.8723 |
| ( >= 93 ) | 33.03% | 74.67% | 65.45% | 1.3041 | 0.8969 |
| ( >= 94 ) | 33.03% | 75.46% | 66.06% | 1.3457 | 0.8876 |
| ( >= 95 ) | 33.03% | 76.24% | 66.67% | 1.3901 | 0.8784 |
| ( >= 96 ) | 32.11% | 76.50% | 66.67% | 1.3665 | 0.8874 |
| ( >= 97 ) | 30.28% | 76.76% | 66.46% | 1.3029 | 0.9083 |
| ( >= 98 ) | 30.28% | 77.81% | 67.28% | 1.3642 | 0.8961 |
| ( >= 99 ) | 28.44% | 78.85% | 67.68% | 1.3448 | 0.9075 |
| ( >= 100 ) | 26.61% | 79.37% | 67.68% | 1.2899 | 0.9247 |
| ( >= 101 ) | 24.77% | 80.42% | 68.09% | 1.265 | 0.9355 |
| ( >= 102 ) | 24.77% | 81.46% | 68.90% | 1.3362 | 0.9235 |
| ( >= 103 ) | 24.77% | 81.72% | 69.11% | 1.3553 | 0.9205 |
| ( >= 104 ) | 23.85% | 82.77% | 69.72% | 1.3842 | 0.92 |
| ( >= 105 ) | 22.02% | 84.07% | 70.33% | 1.3825 | 0.9275 |
| ( >= 105.17 ) | 21.10% | 84.33% | 70.33% | 1.3469 | 0.9356 |
| ( >= 106 ) | 20.18% | 84.33% | 70.12% | 1.2884 | 0.9464 |
| ( >= 107 ) | 19.27% | 85.12% | 70.53% | 1.2945 | 0.9485 |
| ( >= 108 ) | 19.27% | 85.38% | 70.73% | 1.3177 | 0.9456 |
| ( >= 109 ) | 18.35% | 86.42% | 71.34% | 1.3514 | 0.9448 |
| ( >= 110 ) | 18.35% | 86.68% | 71.54% | 1.3779 | 0.9419 |
| ( >= 111 ) | 16.51% | 86.68% | 71.14% | 1.2402 | 0.9631 |
| ( >= 112 ) | 16.51% | 86.95% | 71.34% | 1.265 | 0.9602 |
| ( >= 113 ) | 15.60% | 87.47% | 71.54% | 1.2445 | 0.965 |
| ( >= 113.07 ) | 15.60% | 87.99% | 71.95% | 1.2986 | 0.9592 |
| ( >= 114 ) | 15.60% | 88.25% | 72.15% | 1.3274 | 0.9564 |
| ( >= 115 ) | 14.68% | 88.51% | 72.15% | 1.2777 | 0.964 |
| ( >= 116 ) | 14.68% | 89.30% | 72.76% | 1.3712 | 0.9555 |
| ( >= 117 ) | 13.76% | 89.82% | 72.97% | 1.3514 | 0.9602 |
| ( >= 118 ) | 13.76% | 90.08% | 73.17% | 1.387 | 0.9574 |
| ( >= 119 ) | 13.76% | 91.38% | 74.19% | 1.5972 | 0.9437 |
| ( >= 120 ) | 11.93% | 91.64% | 73.98% | 1.4275 | 0.961 |
| ( >= 121 ) | 11.93% | 92.43% | 74.59% | 1.5751 | 0.9529 |
| ( >= 122 ) | 11.93% | 92.69% | 74.80% | 1.6314 | 0.9502 |
| ( >= 123 ) | 11.01% | 92.69% | 74.59% | 1.5059 | 0.9601 |
| ( >= 124 ) | 11.01% | 93.21% | 75.00% | 1.6217 | 0.9547 |
| ( >= 125 ) | 10.09% | 93.21% | 74.80% | 1.4866 | 0.9646 |
| ( >= 126 ) | 9.17% | 93.21% | 74.59% | 1.3514 | 0.9744 |
| ( >= 127 ) | 8.26% | 94.78% | 75.61% | 1.5812 | 0.968 |
| ( >= 128 ) | 8.26% | 95.04% | 75.81% | 1.6644 | 0.9653 |
| ( >= 131 ) | 8.26% | 95.30% | 76.02% | 1.7569 | 0.9627 |
| ( >= 133 ) | 8.26% | 95.56% | 76.22% | 1.8602 | 0.96 |
| ( >= 134 ) | 7.34% | 96.34% | 76.63% | 2.0079 | 0.9618 |
| ( >= 136 ) | 6.42% | 96.61% | 76.63% | 1.892 | 0.9687 |
| ( >= 137 ) | 6.42% | 97.13% | 77.03% | 2.236 | 0.9635 |
| ( >= 139 ) | 6.42% | 97.65% | 77.44% | 2.7329 | 0.9583 |
| ( >= 140 ) | 4.59% | 97.65% | 77.03% | 1.9521 | 0.9771 |
| ( >= 144 ) | 3.67% | 97.65% | 76.83% | 1.5617 | 0.9865 |
| ( >= 145 ) | 2.75% | 97.65% | 76.63% | 1.1713 | 0.9959 |
| ( >= 148 ) | 2.75% | 98.17% | 77.03% | 1.5059 | 0.9906 |
| ( >= 152 ) | 2.75% | 98.43% | 77.24% | 1.7569 | 0.988 |
| ( >= 153 ) | 2.75% | 98.69% | 77.44% | 2.1083 | 0.9853 |
| ( >= 162 ) | 2.75% | 98.96% | 77.64% | 2.6353 | 0.9827 |
| ( >= 163 ) | 2.75% | 99.22% | 77.85% | 3.5138 | 0.9802 |
| ( >= 164 ) | 1.83% | 99.22% | 77.64% | 2.3425 | 0.9894 |
| ( >= 167 ) | 0.92% | 99.48% | 77.64% | 1.7569 | 0.996 |
| ( >= 169 ) | 0.92% | 99.74% | 77.85% | 3.5137 | 0.9934 |
| ( >= 172 ) | 0.92% | 100.00% | 78.05% |  | 0.9908 |
| ( > 172 ) | 0.00% | 100.00% | 77.85% |  | 1 |

LR: Likelihood ratio

**Table S19:** Performance characteristics table of six-locus PIRCHE-T2 for rejection

| Cutpoint | Sensitivity | Specificity | Overall % correctly classified | LR+ | LR- |
| --- | --- | --- | --- | --- | --- |
| ( >= 0 ) | 100.00% | 0.00% | 41.28% | 1 |  |
| ( >= 7 ) | 100.00% | 6.93% | 45.35% | 1.0745 | 0 |
| ( >= 8 ) | 98.59% | 6.93% | 44.77% | 1.0593 | 0.2032 |
| ( >= 9 ) | 98.59% | 7.92% | 45.35% | 1.0707 | 0.1778 |
| ( >= 11 ) | 98.59% | 8.91% | 45.93% | 1.0824 | 0.1581 |
| ( >= 15 ) | 98.59% | 9.90% | 46.51% | 1.0943 | 0.1423 |
| ( >= 17 ) | 97.18% | 9.90% | 45.93% | 1.0786 | 0.2845 |
| ( >= 21 ) | 97.18% | 10.89% | 46.51% | 1.0906 | 0.2586 |
| ( >= 28 ) | 95.77% | 13.86% | 47.67% | 1.1119 | 0.3048 |
| ( >= 29 ) | 95.77% | 14.85% | 48.26% | 1.1248 | 0.2845 |
| ( >= 30 ) | 95.77% | 15.84% | 48.84% | 1.138 | 0.2667 |
| ( >= 31 ) | 95.77% | 16.83% | 49.42% | 1.1516 | 0.251 |
| ( >= 34 ) | 95.77% | 17.82% | 50.00% | 1.1655 | 0.2371 |
| ( >= 36 ) | 94.37% | 17.82% | 49.42% | 1.1483 | 0.3161 |
| ( >= 39 ) | 94.37% | 22.77% | 52.33% | 1.2219 | 0.2474 |
| ( >= 40 ) | 92.96% | 22.77% | 51.74% | 1.2037 | 0.3092 |
| ( >= 41 ) | 91.55% | 23.76% | 51.74% | 1.2008 | 0.3556 |
| ( >= 44 ) | 88.73% | 23.76% | 50.58% | 1.1639 | 0.4742 |
| ( >= 45 ) | 85.92% | 24.75% | 50.00% | 1.1418 | 0.569 |
| ( >= 45.35 ) | 85.92% | 28.71% | 52.33% | 1.2052 | 0.4905 |
| ( >= 45.78 ) | 85.92% | 29.70% | 52.91% | 1.2222 | 0.4742 |
| ( >= 47 ) | 85.92% | 30.69% | 53.49% | 1.2396 | 0.4589 |
| ( >= 48 ) | 85.92% | 31.68% | 54.07% | 1.2576 | 0.4445 |
| ( >= 49 ) | 85.92% | 32.67% | 54.65% | 1.2761 | 0.4311 |
| ( >= 51 ) | 83.10% | 32.67% | 53.49% | 1.2343 | 0.5173 |
| ( >= 52 ) | 83.10% | 33.66% | 54.07% | 1.2527 | 0.5021 |
| ( >= 53 ) | 83.10% | 34.65% | 54.65% | 1.2717 | 0.4877 |
| ( >= 54 ) | 83.10% | 35.64% | 55.23% | 1.2912 | 0.4742 |
| ( >= 55 ) | 83.10% | 37.62% | 56.40% | 1.3322 | 0.4492 |
| ( >= 57 ) | 81.69% | 39.60% | 56.98% | 1.3526 | 0.4623 |
| ( >= 58 ) | 78.87% | 40.59% | 56.40% | 1.3277 | 0.5204 |
| ( >= 59 ) | 77.46% | 40.59% | 55.81% | 1.304 | 0.5551 |
| ( >= 60 ) | 76.06% | 41.58% | 55.81% | 1.302 | 0.5758 |
| ( >= 62 ) | 76.06% | 42.57% | 56.40% | 1.3244 | 0.5624 |
| ( >= 64 ) | 76.06% | 44.55% | 57.56% | 1.3717 | 0.5374 |
| ( >= 65 ) | 74.65% | 44.55% | 56.98% | 1.3463 | 0.569 |
| ( >= 66 ) | 73.24% | 46.53% | 57.56% | 1.3698 | 0.5751 |
| ( >= 67 ) | 67.61% | 50.50% | 57.56% | 1.3656 | 0.6415 |
| ( >= 68 ) | 64.79% | 50.50% | 56.40% | 1.3087 | 0.6973 |
| ( >= 69 ) | 63.38% | 51.49% | 56.40% | 1.3064 | 0.7113 |
| ( >= 70 ) | 60.56% | 54.46% | 56.98% | 1.3298 | 0.7242 |
| ( >= 72 ) | 60.56% | 55.45% | 57.56% | 1.3593 | 0.7113 |
| ( >= 73 ) | 57.75% | 56.44% | 56.98% | 1.3255 | 0.7487 |
| ( >= 74 ) | 56.34% | 57.43% | 56.98% | 1.3233 | 0.7603 |
| ( >= 75 ) | 52.11% | 57.43% | 55.23% | 1.224 | 0.8339 |
| ( >= 76 ) | 50.70% | 58.42% | 55.23% | 1.2193 | 0.8439 |
| ( >= 77 ) | 49.30% | 59.41% | 55.23% | 1.2144 | 0.8535 |
| ( >= 78 ) | 49.30% | 62.38% | 56.98% | 1.3102 | 0.8129 |
| ( >= 79 ) | 43.66% | 63.37% | 55.23% | 1.1919 | 0.8891 |
| ( >= 80 ) | 43.66% | 64.36% | 55.81% | 1.225 | 0.8754 |
| ( >= 81 ) | 43.66% | 65.35% | 56.40% | 1.26 | 0.8621 |
| ( >= 82 ) | 43.66% | 67.33% | 57.56% | 1.3363 | 0.8368 |
| ( >= 83 ) | 40.85% | 67.33% | 56.40% | 1.2501 | 0.8786 |
| ( >= 84 ) | 40.85% | 69.31% | 57.56% | 1.3308 | 0.8535 |
| ( >= 85 ) | 39.44% | 70.30% | 57.56% | 1.3277 | 0.8615 |
| ( >= 86 ) | 38.03% | 71.29% | 57.56% | 1.3244 | 0.8693 |
| ( >= 88 ) | 36.62% | 71.29% | 56.98% | 1.2754 | 0.8891 |
| ( >= 90 ) | 35.21% | 71.29% | 56.40% | 1.2263 | 0.9088 |
| ( >= 91 ) | 35.21% | 72.28% | 56.98% | 1.2701 | 0.8964 |
| ( >= 92 ) | 33.80% | 72.28% | 56.40% | 1.2193 | 0.9159 |
| ( >= 93 ) | 33.80% | 73.27% | 56.98% | 1.2645 | 0.9035 |
| ( >= 94 ) | 33.80% | 75.25% | 58.14% | 1.3656 | 0.8797 |
| ( >= 96 ) | 32.39% | 75.25% | 57.56% | 1.3087 | 0.8984 |
| ( >= 98 ) | 29.58% | 76.24% | 56.98% | 1.2447 | 0.9237 |
| ( >= 99 ) | 29.58% | 78.22% | 58.14% | 1.3579 | 0.9003 |
| ( >= 100 ) | 28.17% | 78.22% | 57.56% | 1.2932 | 0.9183 |
| ( >= 101 ) | 26.76% | 79.21% | 57.56% | 1.2871 | 0.9246 |
| ( >= 103 ) | 26.76% | 81.19% | 58.72% | 1.4225 | 0.9021 |
| ( >= 104 ) | 25.35% | 82.18% | 58.72% | 1.4225 | 0.9084 |
| ( >= 105 ) | 23.94% | 82.18% | 58.14% | 1.3435 | 0.9255 |
| ( >= 106 ) | 23.94% | 83.17% | 58.72% | 1.4225 | 0.9145 |
| ( >= 108 ) | 21.13% | 84.16% | 58.14% | 1.3336 | 0.9372 |
| ( >= 109 ) | 19.72% | 86.14% | 58.72% | 1.4225 | 0.932 |
| ( >= 110 ) | 19.72% | 87.13% | 59.30% | 1.532 | 0.9214 |
| ( >= 111 ) | 18.31% | 88.12% | 59.30% | 1.5411 | 0.927 |
| ( >= 112 ) | 16.90% | 88.12% | 58.72% | 1.4225 | 0.943 |
| ( >= 113.07 ) | 15.49% | 88.12% | 58.14% | 1.304 | 0.959 |
| ( >= 114 ) | 14.08% | 88.12% | 57.56% | 1.1854 | 0.975 |
| ( >= 115 ) | 12.68% | 88.12% | 56.98% | 1.0669 | 0.991 |
| ( >= 116 ) | 12.68% | 89.11% | 57.56% | 1.1639 | 0.98 |
| ( >= 118 ) | 12.68% | 91.09% | 58.72% | 1.4225 | 0.9587 |
| ( >= 119 ) | 12.68% | 94.06% | 60.47% | 2.1338 | 0.9284 |
| ( >= 123 ) | 12.68% | 95.05% | 61.05% | 2.5606 | 0.9187 |
| ( >= 124 ) | 12.68% | 96.04% | 61.63% | 3.2007 | 0.9092 |
| ( >= 125 ) | 11.27% | 96.04% | 61.05% | 2.8451 | 0.9239 |
| ( >= 126 ) | 9.86% | 96.04% | 60.47% | 2.4894 | 0.9386 |
| ( >= 133 ) | 8.45% | 96.04% | 59.88% | 2.1338 | 0.9532 |
| ( >= 139 ) | 8.45% | 98.02% | 61.05% | 4.2676 | 0.934 |
| ( >= 140 ) | 7.04% | 98.02% | 60.47% | 3.5563 | 0.9484 |
| ( >= 148 ) | 5.63% | 98.02% | 59.88% | 2.8451 | 0.9627 |
| ( >= 152 ) | 4.23% | 98.02% | 59.30% | 2.1338 | 0.9771 |
| ( >= 162 ) | 4.23% | 99.01% | 59.88% | 4.2676 | 0.9673 |
| ( >= 163 ) | 4.23% | 100.00% | 60.47% |  | 0.9577 |
| ( >= 164 ) | 2.82% | 100.00% | 59.88% |  | 0.9718 |
| ( >= 172 ) | 1.41% | 100.00% | 59.30% |  | 0.9859 |
| ( > 172 ) | 0.00% | 100.00% | 58.72% |  | 1 |

LR: Likelihood ratio

**Table S20:** Performance characteristics table of six-locus PIRCHE-T2 for donor-derived cell-free DNA

| Cutpoint | Sensitivity | Specificity | Overall % correctly classified | LR+ | LR- |
| --- | --- | --- | --- | --- | --- |
| ( >= 0 ) | 100.00% | 0.00% | 19.64% | 1 |  |
| ( >= 1 ) | 100.00% | 4.44% | 23.21% | 1.0465 | 0 |
| ( >= 8 ) | 100.00% | 4.76% | 23.47% | 1.05 | 0 |
| ( >= 10 ) | 100.00% | 5.71% | 24.23% | 1.0606 | 0 |
| ( >= 11 ) | 100.00% | 6.35% | 24.74% | 1.0678 | 0 |
| ( >= 12 ) | 100.00% | 6.67% | 25.00% | 1.0714 | 0 |
| ( >= 14 ) | 100.00% | 6.98% | 25.26% | 1.0751 | 0 |
| ( >= 15 ) | 100.00% | 7.30% | 25.51% | 1.0788 | 0 |
| ( >= 16 ) | 98.70% | 7.94% | 25.77% | 1.0721 | 0.1636 |
| ( >= 17 ) | 98.70% | 8.25% | 26.02% | 1.0758 | 0.1573 |
| ( >= 20 ) | 98.70% | 9.21% | 26.79% | 1.0871 | 0.1411 |
| ( >= 21 ) | 97.40% | 9.84% | 27.04% | 1.0803 | 0.2639 |
| ( >= 22 ) | 96.10% | 10.48% | 27.30% | 1.0735 | 0.3719 |
| ( >= 23 ) | 96.10% | 10.79% | 27.55% | 1.0773 | 0.361 |
| ( >= 26 ) | 93.51% | 10.79% | 27.04% | 1.0482 | 0.6016 |
| ( >= 28 ) | 93.51% | 11.11% | 27.30% | 1.0519 | 0.5844 |
| ( >= 30 ) | 93.51% | 11.43% | 27.55% | 1.0557 | 0.5682 |
| ( >= 31 ) | 93.51% | 12.06% | 28.06% | 1.0633 | 0.5383 |
| ( >= 31.71 ) | 93.51% | 13.02% | 28.83% | 1.075 | 0.4989 |
| ( >= 32 ) | 93.51% | 13.33% | 29.08% | 1.0789 | 0.487 |
| ( >= 33 ) | 92.21% | 15.24% | 30.36% | 1.0878 | 0.5114 |
| ( >= 34 ) | 92.21% | 16.19% | 31.12% | 1.1002 | 0.4813 |
| ( >= 35 ) | 92.21% | 16.51% | 31.38% | 1.1044 | 0.472 |
| ( >= 36 ) | 92.21% | 17.46% | 32.14% | 1.1171 | 0.4463 |
| ( >= 38 ) | 92.21% | 19.68% | 33.93% | 1.148 | 0.3959 |
| ( >= 39 ) | 92.21% | 21.59% | 35.46% | 1.1759 | 0.361 |
| ( >= 40 ) | 92.21% | 21.90% | 35.71% | 1.1807 | 0.3557 |
| ( >= 41 ) | 92.21% | 22.86% | 36.48% | 1.1953 | 0.3409 |
| ( >= 42 ) | 90.91% | 24.13% | 37.24% | 1.1982 | 0.3768 |
| ( >= 43 ) | 89.61% | 24.76% | 37.50% | 1.191 | 0.4196 |
| ( >= 44 ) | 89.61% | 25.08% | 37.76% | 1.1961 | 0.4143 |
| ( >= 45 ) | 89.61% | 25.40% | 38.01% | 1.2012 | 0.4091 |
| ( >= 45.35 ) | 89.61% | 27.30% | 39.54% | 1.2326 | 0.3805 |
| ( >= 46 ) | 89.61% | 27.62% | 39.80% | 1.238 | 0.3762 |
| ( >= 47 ) | 89.61% | 28.25% | 40.31% | 1.249 | 0.3677 |
| ( >= 48 ) | 89.61% | 29.21% | 41.07% | 1.2658 | 0.3557 |
| ( >= 48.19 ) | 89.61% | 29.84% | 41.58% | 1.2773 | 0.3482 |
| ( >= 49 ) | 89.61% | 30.16% | 41.84% | 1.2831 | 0.3445 |
| ( >= 50 ) | 87.01% | 30.79% | 41.84% | 1.2573 | 0.4217 |
| ( >= 51 ) | 85.71% | 31.43% | 42.09% | 1.25 | 0.4545 |
| ( >= 52 ) | 85.71% | 32.06% | 42.60% | 1.2617 | 0.4455 |
| ( >= 53 ) | 85.71% | 33.65% | 43.88% | 1.2919 | 0.4245 |
| ( >= 54 ) | 85.71% | 34.60% | 44.64% | 1.3107 | 0.4128 |
| ( >= 55 ) | 85.71% | 36.83% | 46.43% | 1.3568 | 0.3879 |
| ( >= 56 ) | 84.42% | 37.78% | 46.94% | 1.3567 | 0.4125 |
| ( >= 57 ) | 83.12% | 39.37% | 47.96% | 1.3708 | 0.4289 |
| ( >= 58 ) | 81.82% | 40.95% | 48.98% | 1.3856 | 0.444 |
| ( >= 59 ) | 79.22% | 42.54% | 49.74% | 1.3787 | 0.4885 |
| ( >= 60 ) | 79.22% | 43.49% | 50.51% | 1.4019 | 0.4778 |
| ( >= 61 ) | 77.92% | 44.76% | 51.28% | 1.4107 | 0.4932 |
| ( >= 62 ) | 77.92% | 45.08% | 51.53% | 1.4188 | 0.4898 |
| ( >= 63 ) | 77.92% | 46.03% | 52.30% | 1.4439 | 0.4796 |
| ( >= 64 ) | 76.62% | 46.03% | 52.04% | 1.4198 | 0.5078 |
| ( >= 65 ) | 75.32% | 47.30% | 52.81% | 1.4294 | 0.5217 |
| ( >= 66 ) | 72.73% | 47.30% | 52.30% | 1.3801 | 0.5766 |
| ( >= 67 ) | 70.13% | 49.21% | 53.32% | 1.3807 | 0.607 |
| ( >= 68 ) | 68.83% | 50.79% | 54.34% | 1.3988 | 0.6136 |
| ( >= 69 ) | 67.53% | 51.43% | 54.59% | 1.3904 | 0.6313 |
| ( >= 70 ) | 66.23% | 53.97% | 56.38% | 1.4389 | 0.6257 |
| ( >= 71 ) | 66.23% | 54.92% | 57.14% | 1.4693 | 0.6148 |
| ( >= 72 ) | 66.23% | 55.56% | 57.65% | 1.4903 | 0.6078 |
| ( >= 73 ) | 64.94% | 56.83% | 58.42% | 1.504 | 0.6171 |
| ( >= 73.8 ) | 63.64% | 57.46% | 58.67% | 1.4959 | 0.6328 |
| ( >= 74 ) | 63.64% | 57.78% | 58.93% | 1.5072 | 0.6294 |
| ( >= 75 ) | 59.74% | 58.41% | 58.67% | 1.4365 | 0.6892 |
| ( >= 76 ) | 57.14% | 59.05% | 58.67% | 1.3953 | 0.7258 |
| ( >= 77 ) | 55.84% | 59.68% | 58.93% | 1.3851 | 0.7398 |
| ( >= 78 ) | 54.55% | 61.27% | 59.95% | 1.4083 | 0.7419 |
| ( >= 79 ) | 50.65% | 64.76% | 61.99% | 1.4373 | 0.762 |
| ( >= 80 ) | 49.35% | 65.08% | 61.99% | 1.4132 | 0.7783 |
| ( >= 81 ) | 49.35% | 66.35% | 63.01% | 1.4666 | 0.7634 |
| ( >= 83 ) | 46.75% | 67.30% | 63.27% | 1.4298 | 0.7912 |
| ( >= 84 ) | 45.45% | 68.89% | 64.29% | 1.461 | 0.7918 |
| ( >= 84.08 ) | 42.86% | 69.52% | 64.29% | 1.4063 | 0.8219 |
| ( >= 85 ) | 41.56% | 69.52% | 64.03% | 1.3636 | 0.8406 |
| ( >= 86 ) | 41.56% | 69.84% | 64.29% | 1.378 | 0.8368 |
| ( >= 87 ) | 38.96% | 69.84% | 63.78% | 1.2919 | 0.874 |
| ( >= 87.81 ) | 38.96% | 70.79% | 64.54% | 1.334 | 0.8622 |
| ( >= 88 ) | 38.96% | 71.11% | 64.80% | 1.3487 | 0.8584 |
| ( >= 89 ) | 37.66% | 72.70% | 65.82% | 1.3795 | 0.8575 |
| ( >= 90 ) | 37.66% | 73.02% | 66.07% | 1.3957 | 0.8538 |
| ( >= 91 ) | 37.66% | 73.33% | 66.33% | 1.4123 | 0.8501 |
| ( >= 92 ) | 37.66% | 74.29% | 67.09% | 1.4646 | 0.8392 |
| ( >= 93 ) | 36.36% | 74.29% | 66.84% | 1.4141 | 0.8566 |
| ( >= 94 ) | 36.36% | 74.92% | 67.35% | 1.4499 | 0.8494 |
| ( >= 95 ) | 35.06% | 75.56% | 67.60% | 1.4345 | 0.8594 |
| ( >= 96 ) | 35.06% | 76.19% | 68.11% | 1.4727 | 0.8523 |
| ( >= 97 ) | 31.17% | 76.19% | 67.35% | 1.3091 | 0.9034 |
| ( >= 98 ) | 29.87% | 77.14% | 67.86% | 1.3068 | 0.9091 |
| ( >= 99 ) | 27.27% | 78.10% | 68.11% | 1.2451 | 0.9313 |
| ( >= 100 ) | 25.97% | 78.73% | 68.37% | 1.2212 | 0.9402 |
| ( >= 101 ) | 24.68% | 80.00% | 69.13% | 1.2338 | 0.9416 |
| ( >= 102 ) | 24.68% | 81.27% | 70.15% | 1.3174 | 0.9268 |
| ( >= 103 ) | 24.68% | 81.59% | 70.41% | 1.3401 | 0.9232 |
| ( >= 104 ) | 22.08% | 82.22% | 70.41% | 1.2419 | 0.9477 |
| ( >= 105 ) | 20.78% | 83.49% | 71.17% | 1.2587 | 0.9488 |
| ( >= 105.17 ) | 19.48% | 83.81% | 71.17% | 1.2032 | 0.9607 |
| ( >= 106 ) | 19.48% | 84.13% | 71.43% | 1.2273 | 0.9571 |
| ( >= 107 ) | 15.58% | 84.13% | 70.66% | 0.9818 | 1.0034 |
| ( >= 108 ) | 15.58% | 84.44% | 70.92% | 1.0019 | 0.9997 |
| ( >= 109 ) | 15.58% | 85.71% | 71.94% | 1.0909 | 0.9848 |
| ( >= 110 ) | 15.58% | 86.03% | 72.19% | 1.1157 | 0.9812 |
| ( >= 112 ) | 15.58% | 86.35% | 72.45% | 1.1416 | 0.9776 |
| ( >= 113 ) | 14.29% | 86.98% | 72.70% | 1.0976 | 0.9854 |
| ( >= 114 ) | 14.29% | 87.62% | 73.21% | 1.1538 | 0.9783 |
| ( >= 115 ) | 14.29% | 87.94% | 73.47% | 1.1842 | 0.9747 |
| ( >= 116 ) | 12.99% | 87.94% | 73.21% | 1.0766 | 0.9895 |
| ( >= 118 ) | 12.99% | 88.57% | 73.72% | 1.1364 | 0.9824 |
| ( >= 119 ) | 11.69% | 89.84% | 74.49% | 1.1506 | 0.983 |
| ( >= 120 ) | 11.69% | 90.79% | 75.26% | 1.2696 | 0.9727 |
| ( >= 121 ) | 11.69% | 91.43% | 75.77% | 1.3636 | 0.9659 |
| ( >= 123 ) | 11.69% | 91.75% | 76.02% | 1.4161 | 0.9626 |
| ( >= 124 ) | 10.39% | 92.06% | 76.02% | 1.3091 | 0.9734 |
| ( >= 125 ) | 9.09% | 92.06% | 75.77% | 1.1455 | 0.9875 |
| ( >= 126 ) | 7.79% | 92.06% | 75.51% | 0.9818 | 1.0016 |
| ( >= 127 ) | 5.19% | 93.33% | 76.02% | 0.7792 | 1.0158 |
| ( >= 128 ) | 5.19% | 93.65% | 76.28% | 0.8182 | 1.0123 |
| ( >= 131 ) | 5.19% | 93.97% | 76.53% | 0.8612 | 1.0089 |
| ( >= 133 ) | 5.19% | 94.29% | 76.79% | 0.9091 | 1.0055 |
| ( >= 134 ) | 5.19% | 95.24% | 77.55% | 1.0909 | 0.9955 |
| ( >= 136 ) | 5.19% | 95.87% | 78.06% | 1.2587 | 0.9889 |
| ( >= 137 ) | 5.19% | 96.51% | 78.57% | 1.4876 | 0.9824 |
| ( >= 139 ) | 5.19% | 96.83% | 78.83% | 1.6364 | 0.9791 |
| ( >= 140 ) | 3.90% | 97.14% | 78.83% | 1.3636 | 0.9893 |
| ( >= 144 ) | 2.60% | 97.14% | 78.57% | 0.9091 | 1.0027 |
| ( >= 145 ) | 2.60% | 97.46% | 78.83% | 1.0227 | 0.9994 |
| ( >= 146 ) | 2.60% | 98.10% | 79.34% | 1.3636 | 0.9929 |
| ( >= 148 ) | 2.60% | 98.41% | 79.59% | 1.6364 | 0.9897 |
| ( >= 152 ) | 2.60% | 98.73% | 79.85% | 2.0455 | 0.9866 |
| ( >= 153 ) | 2.60% | 99.05% | 80.10% | 2.7273 | 0.9834 |
| ( >= 164 ) | 2.60% | 99.37% | 80.36% | 4.0909 | 0.9802 |
| ( >= 167 ) | 1.30% | 99.37% | 80.10% | 2.0455 | 0.9933 |
| ( >= 169 ) | 1.30% | 99.68% | 80.36% | 4.0909 | 0.9902 |
| ( >= 172 ) | 1.30% | 100.00% | 80.61% |  | 0.987 |
| ( > 172 ) | 0.00% | 100.00% | 80.36% |  | 1 |

LR: Likelihood ratio

**
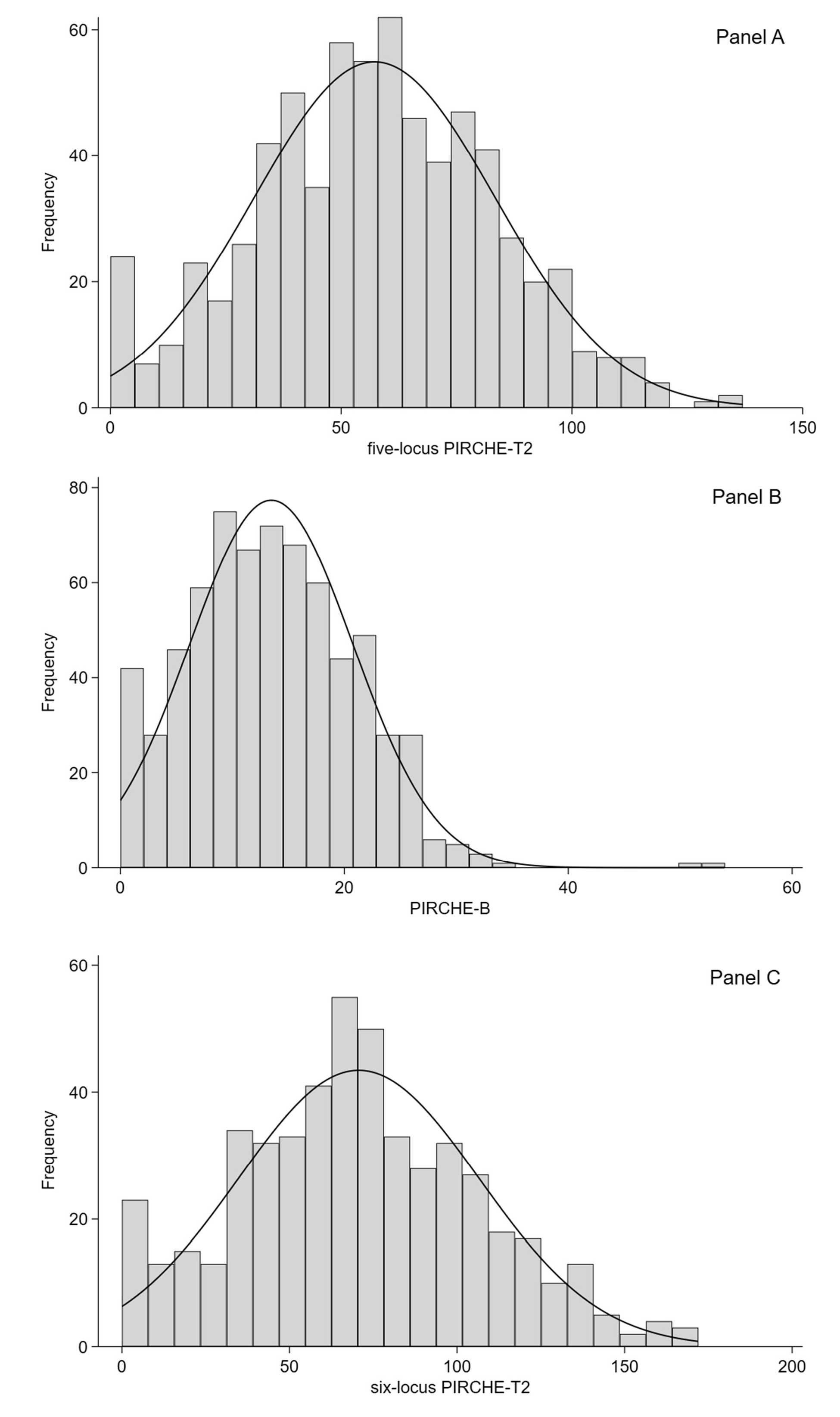
Figure S1**

**Figure S2:**

**
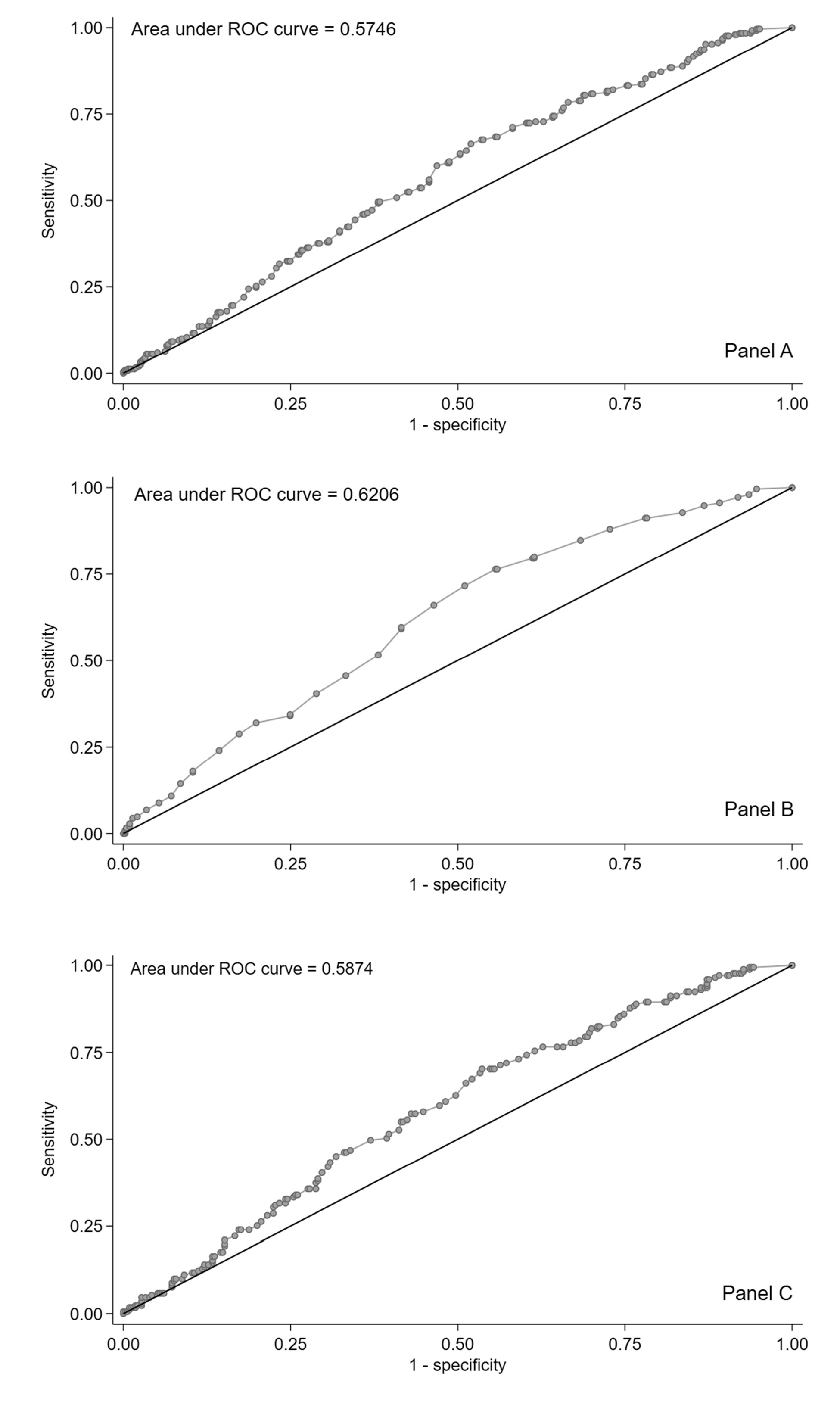
**

**Figure S3**

**
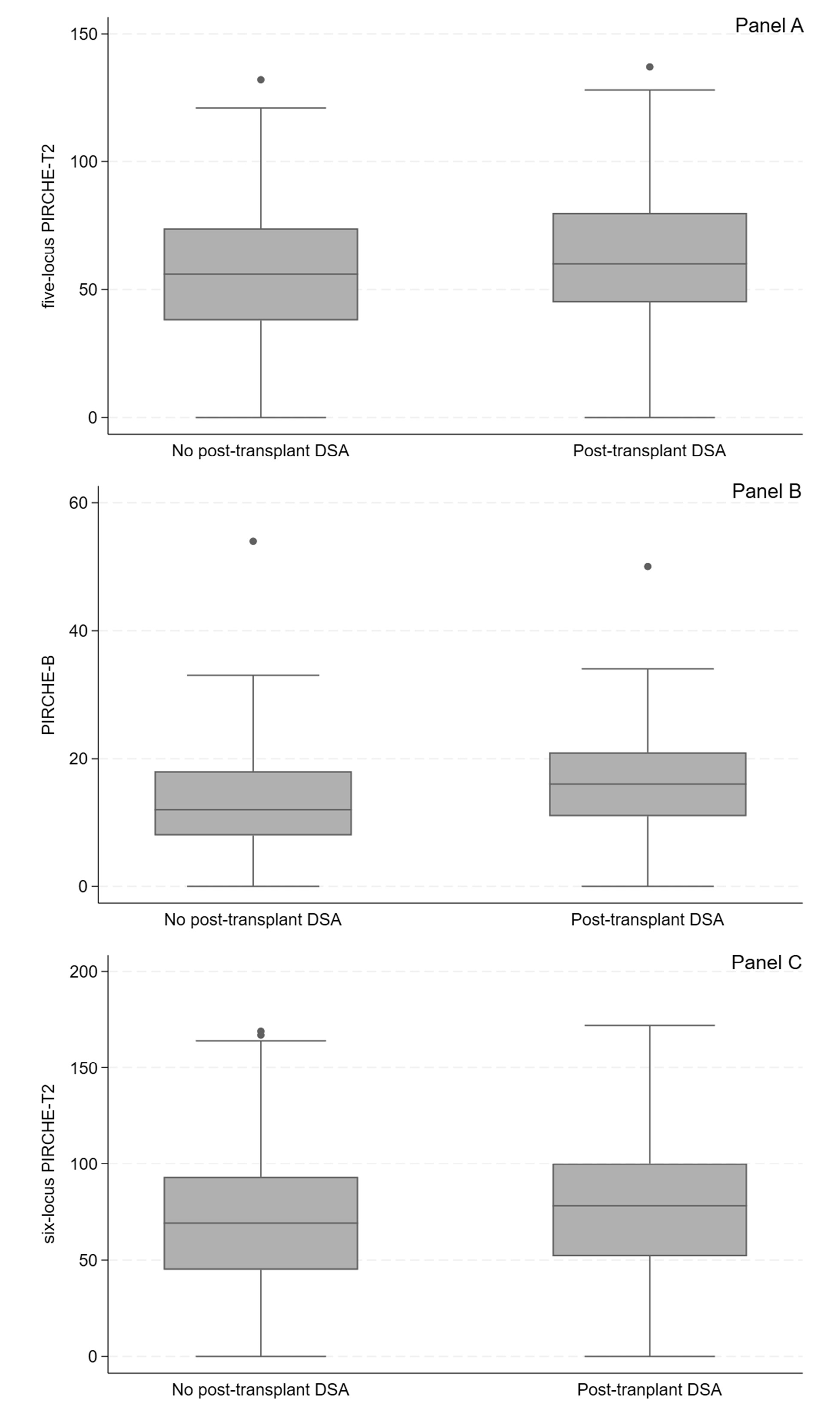
**

**Figure S4**

**
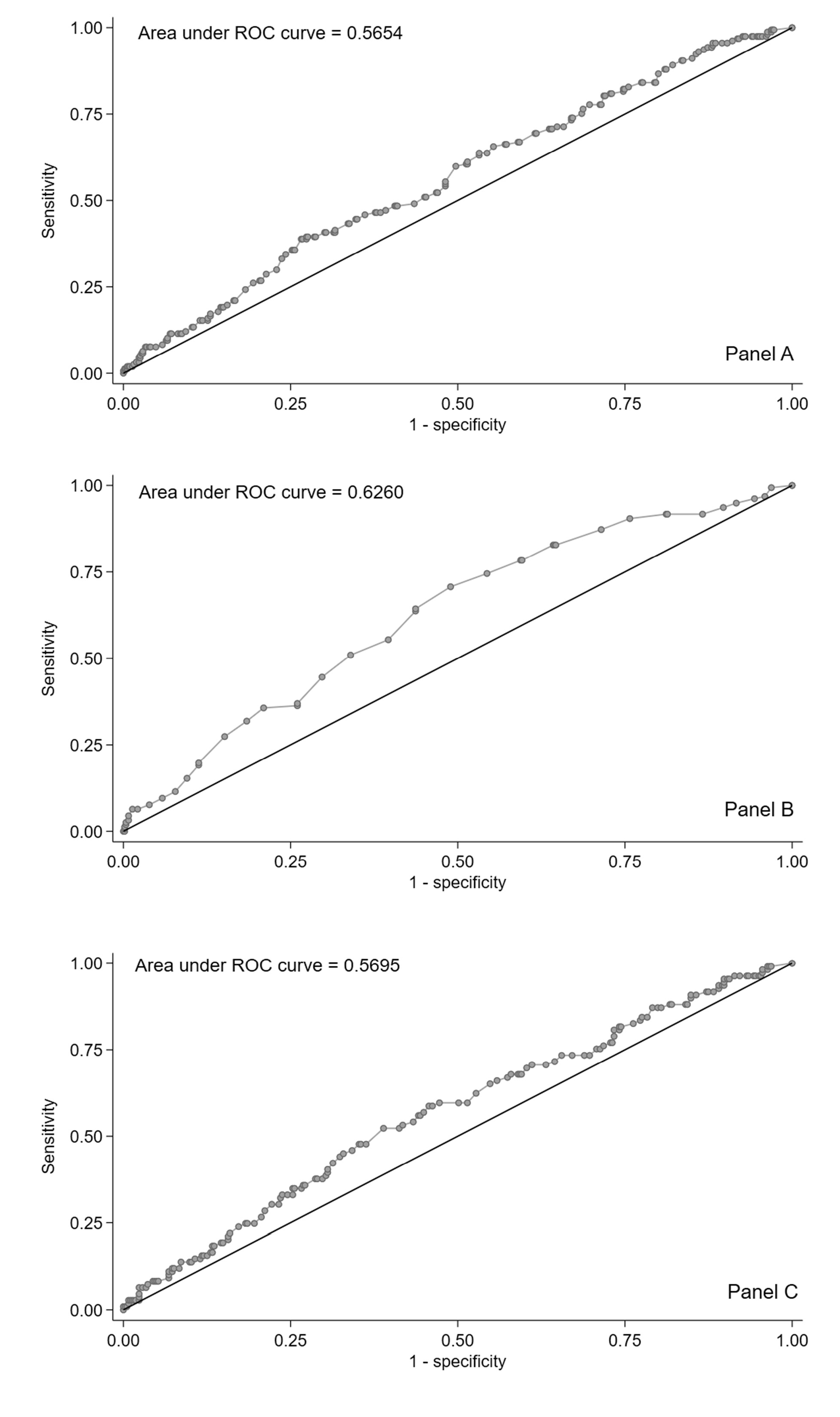
**

**Figure S5**

**
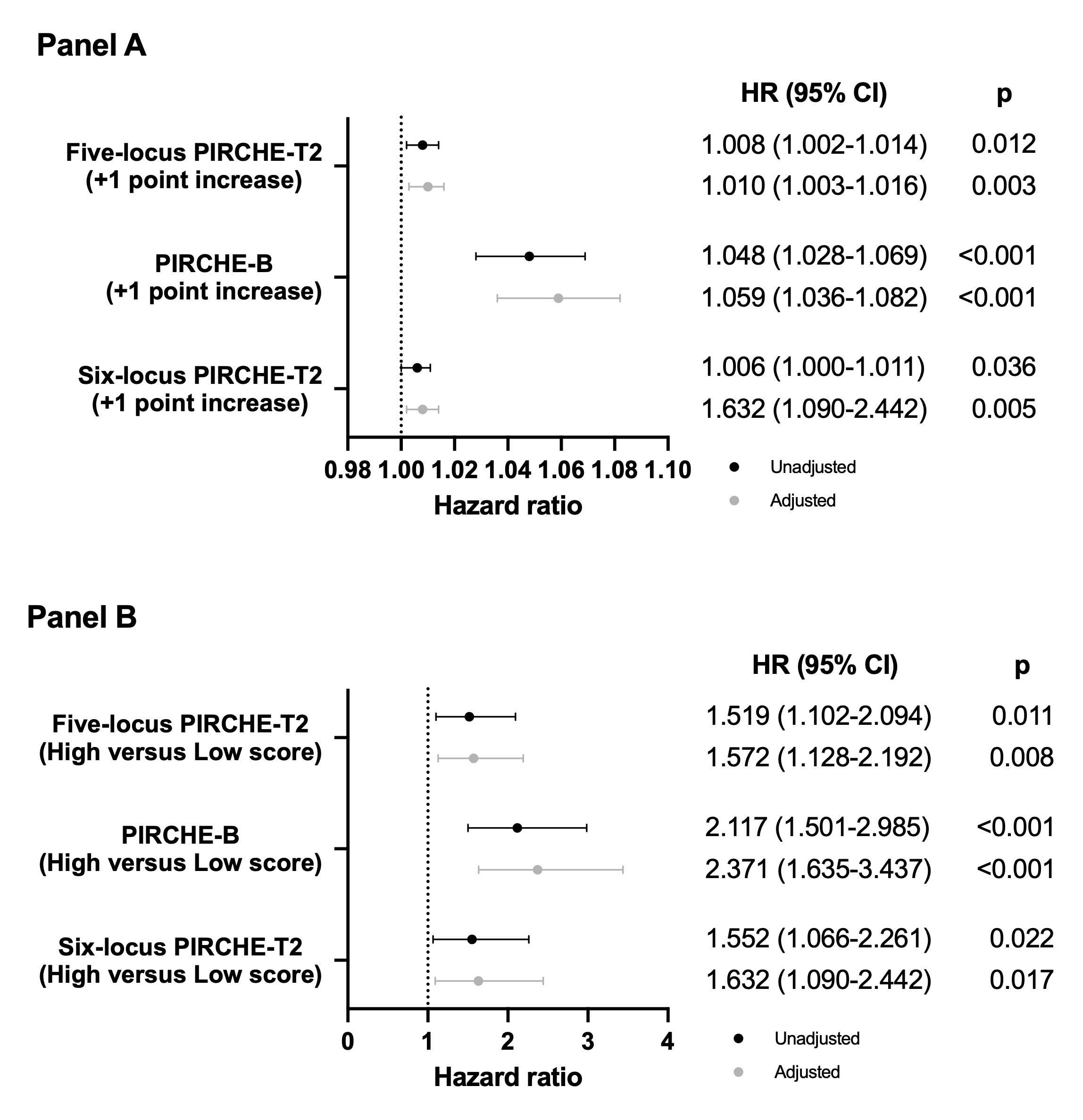
**

**Figure S6**

**
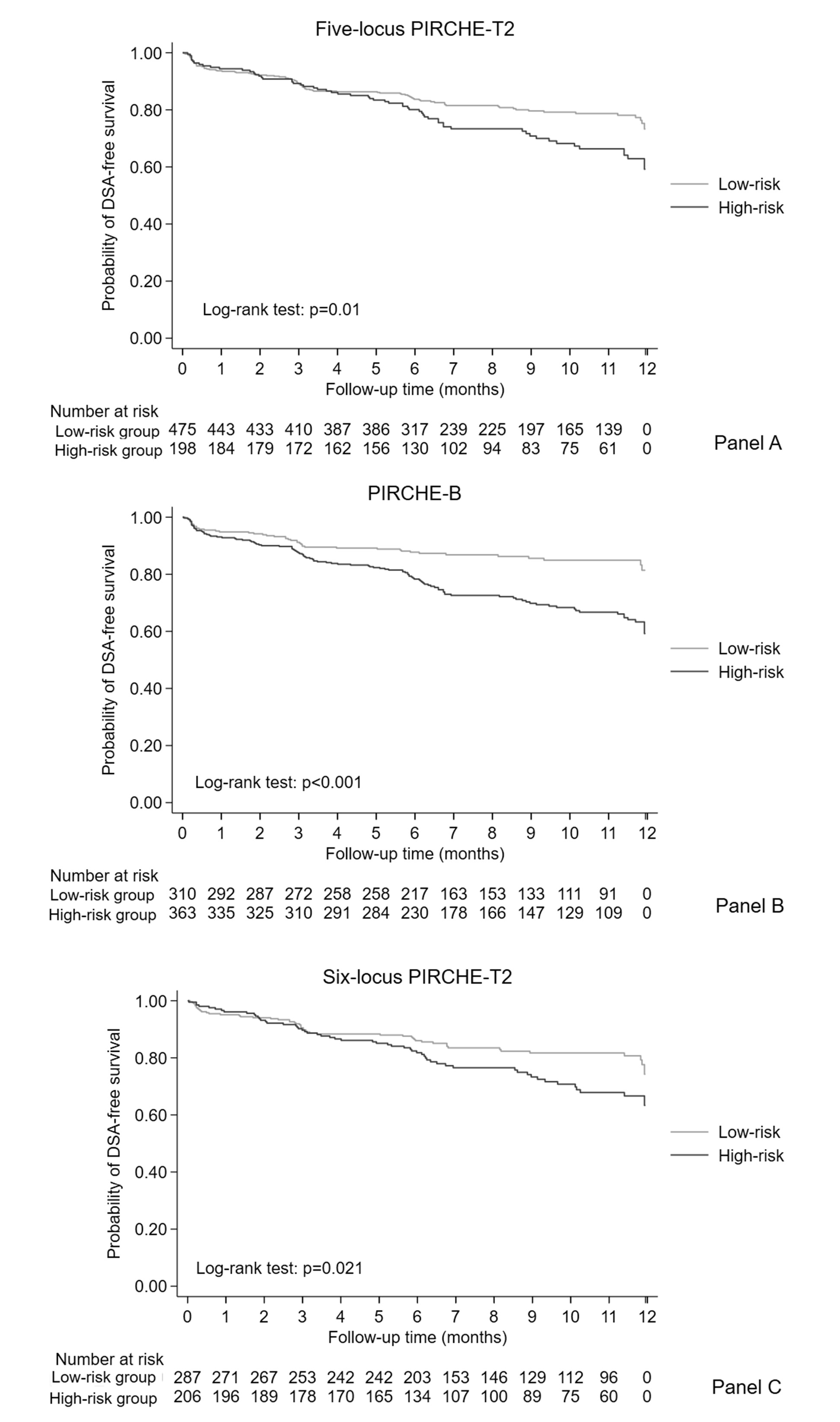
**

**Figure S7**

**
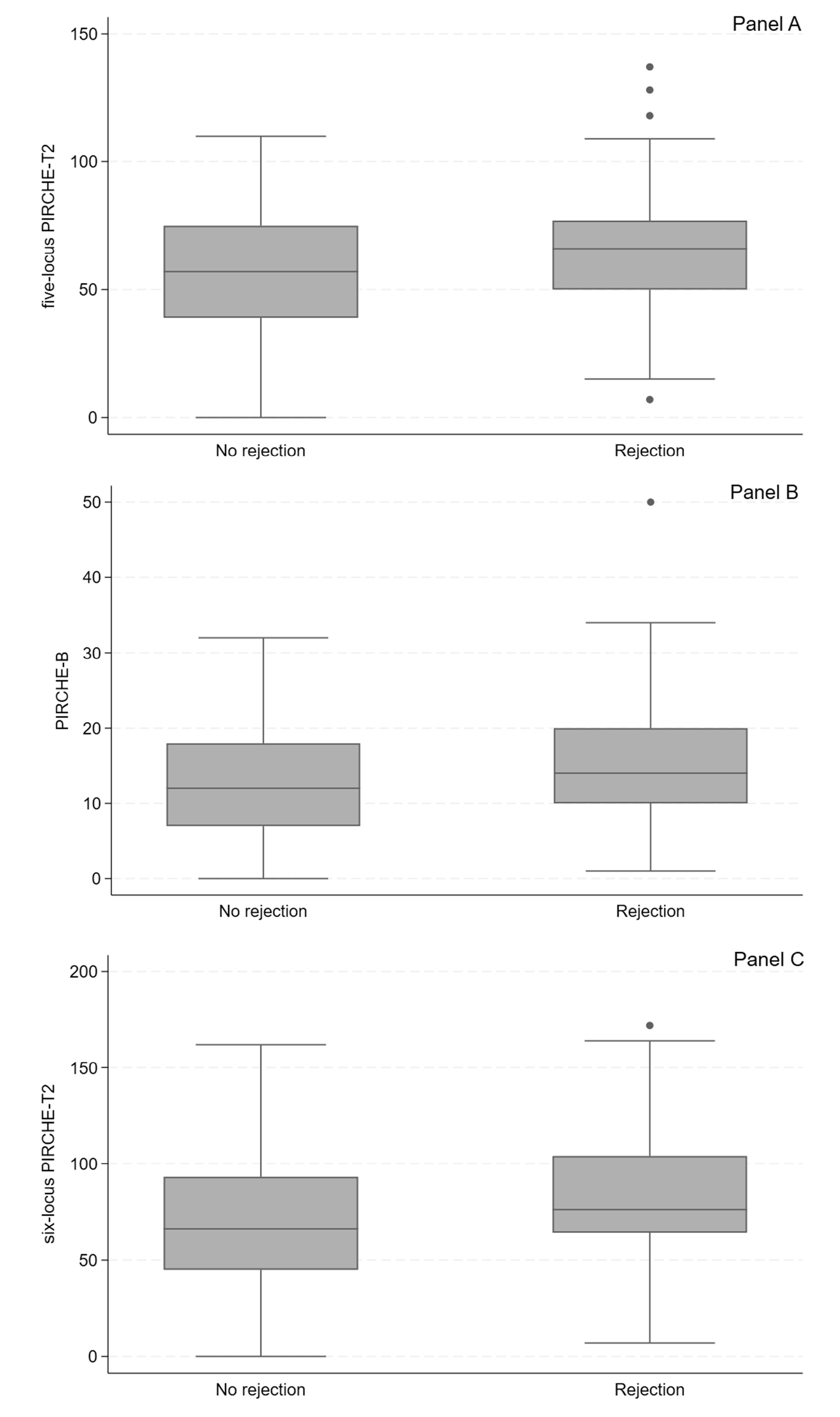
**

**Figure S8**

**
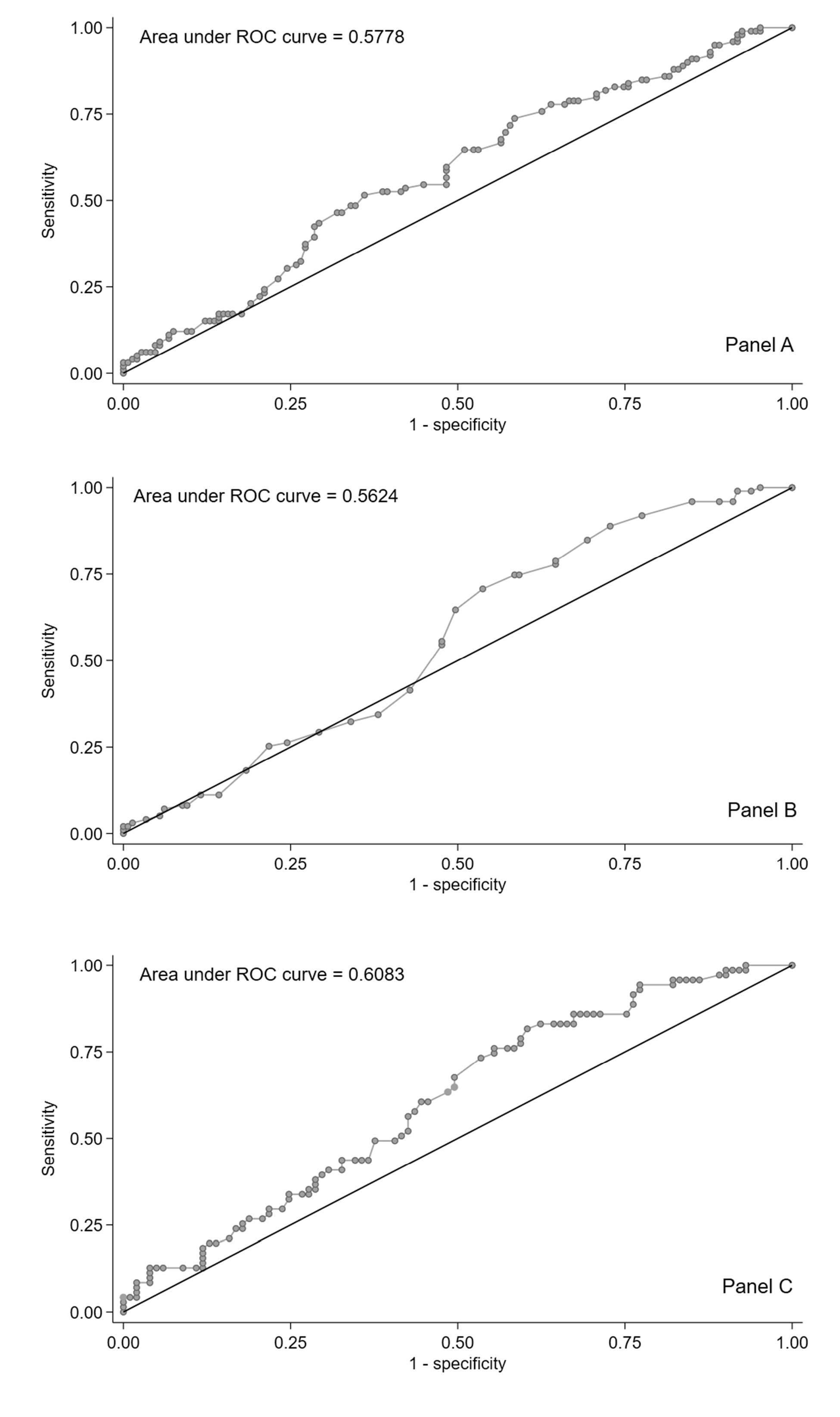
**

**Figure S9**

**
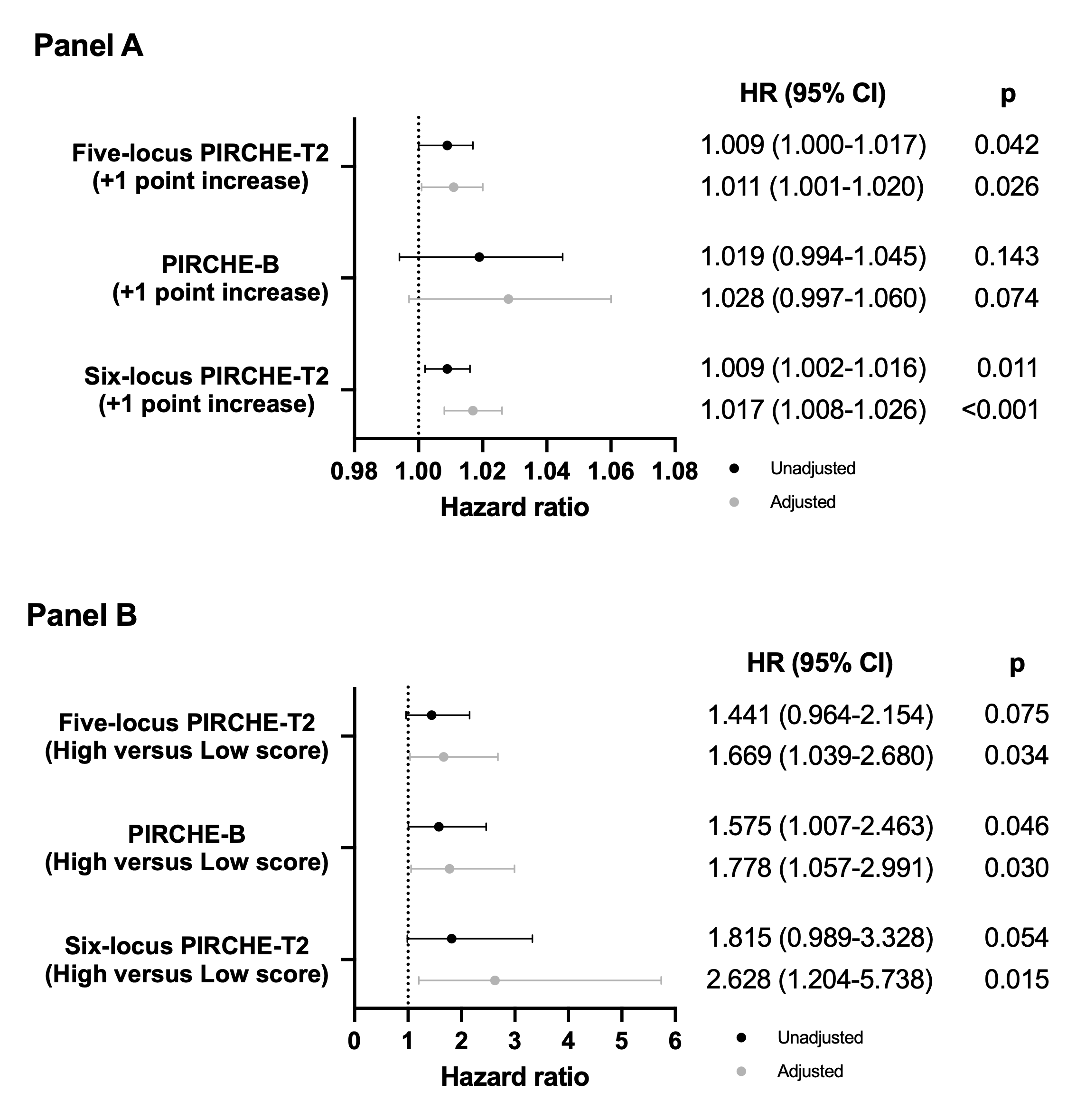
**

**Figure S10**

**
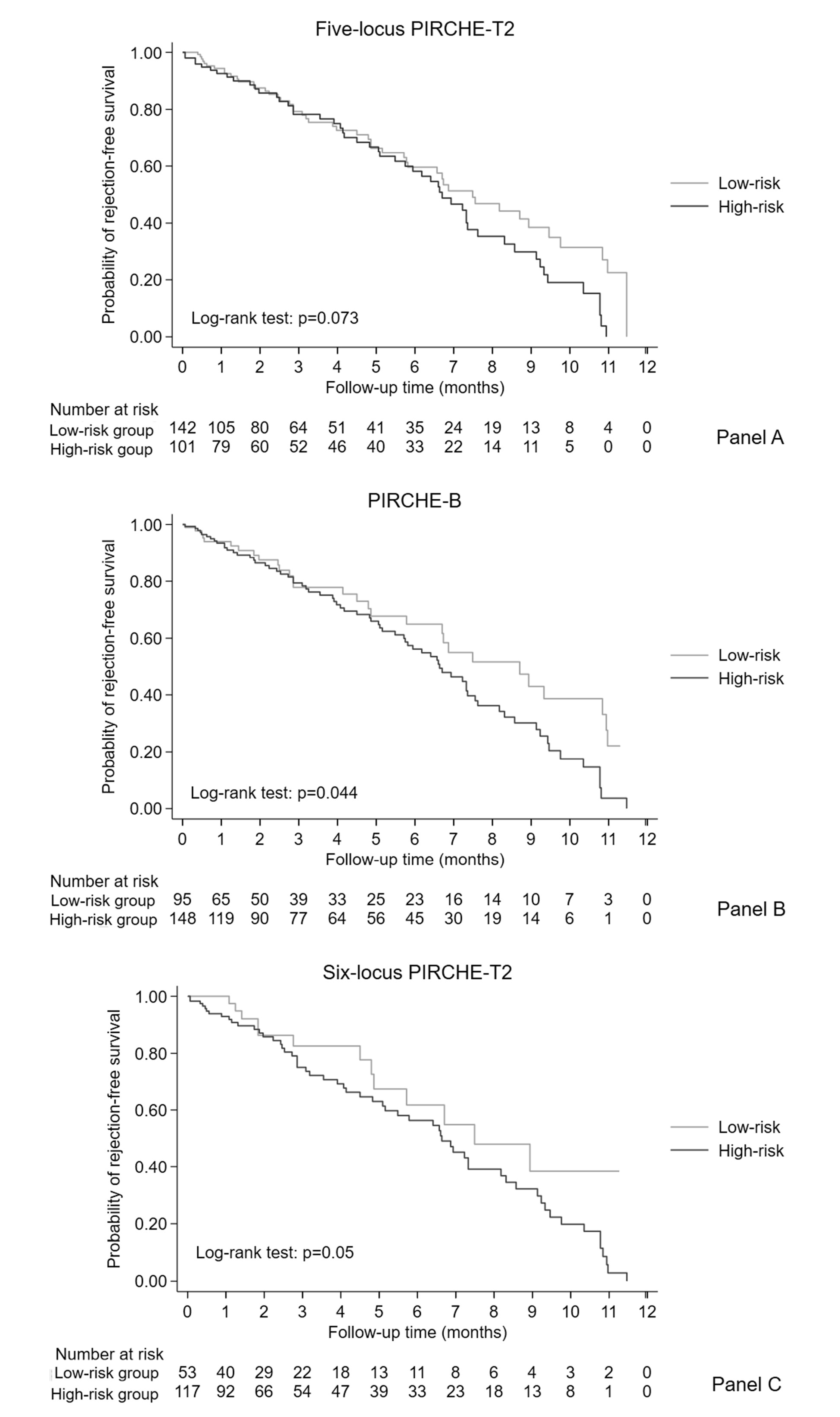
**

**Figure S11**

**
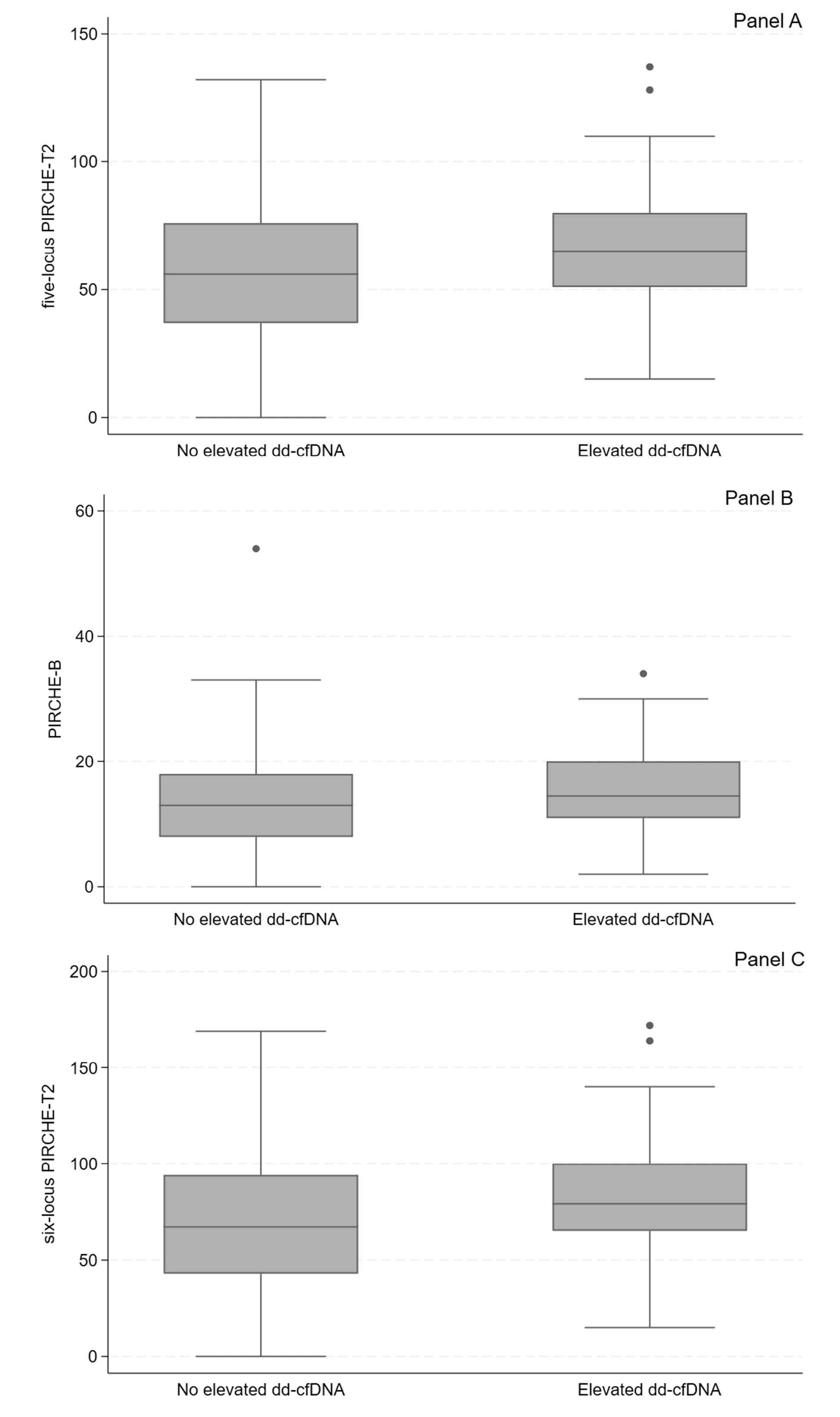
**

**Figure S12**

**
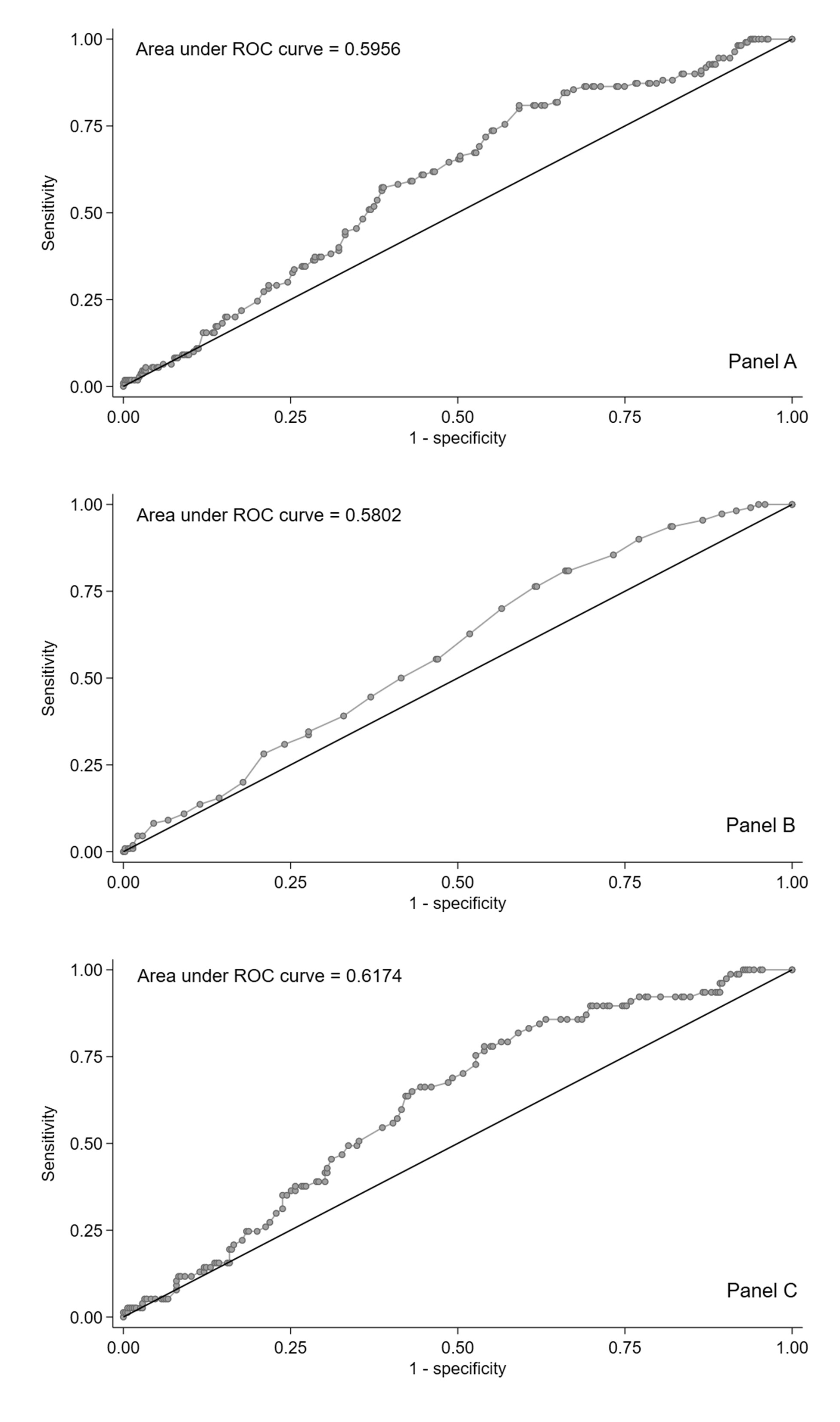
**

**Figure S13**

**
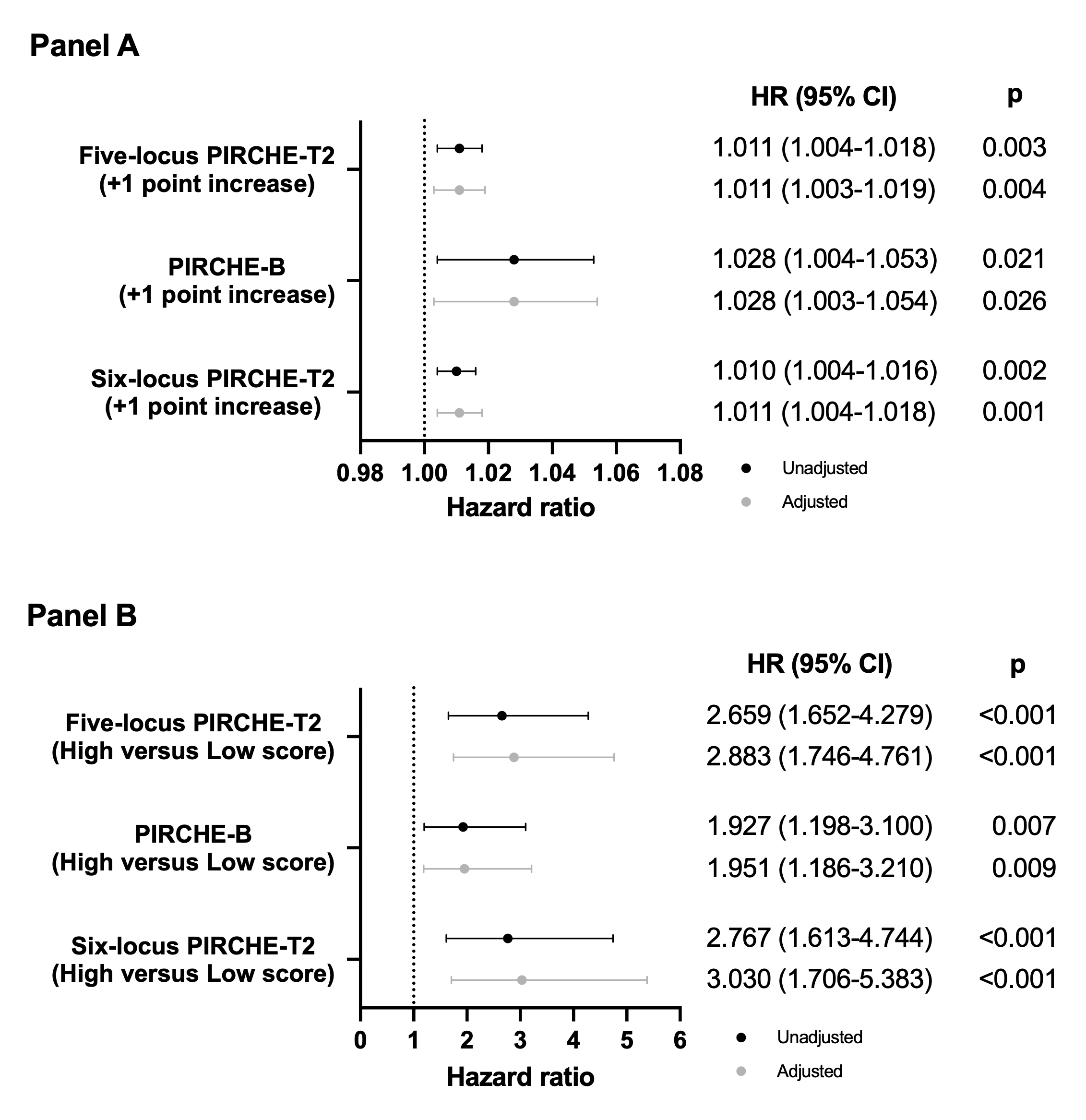
**

**Figure S14**

**
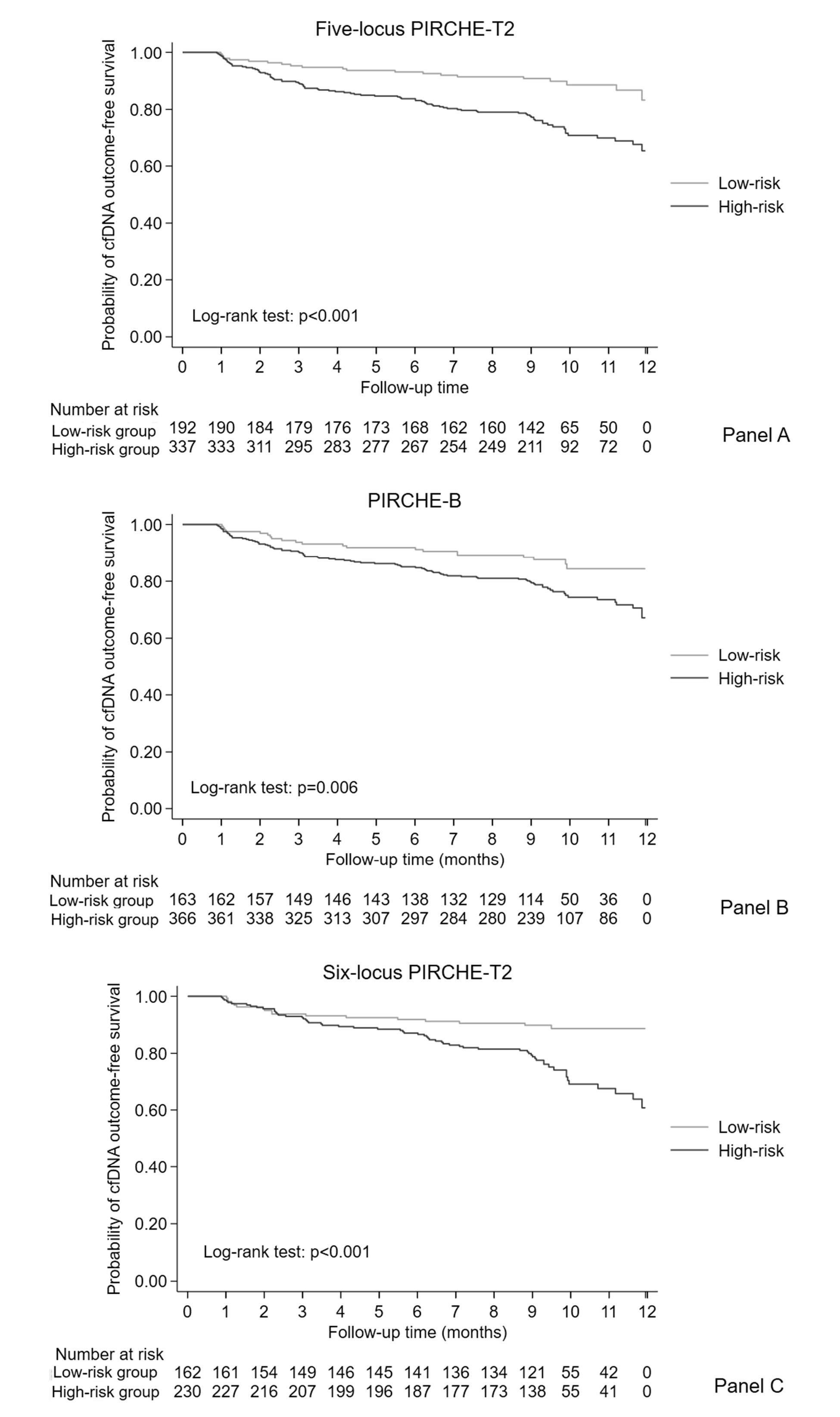
**

**Figure S15:**

**
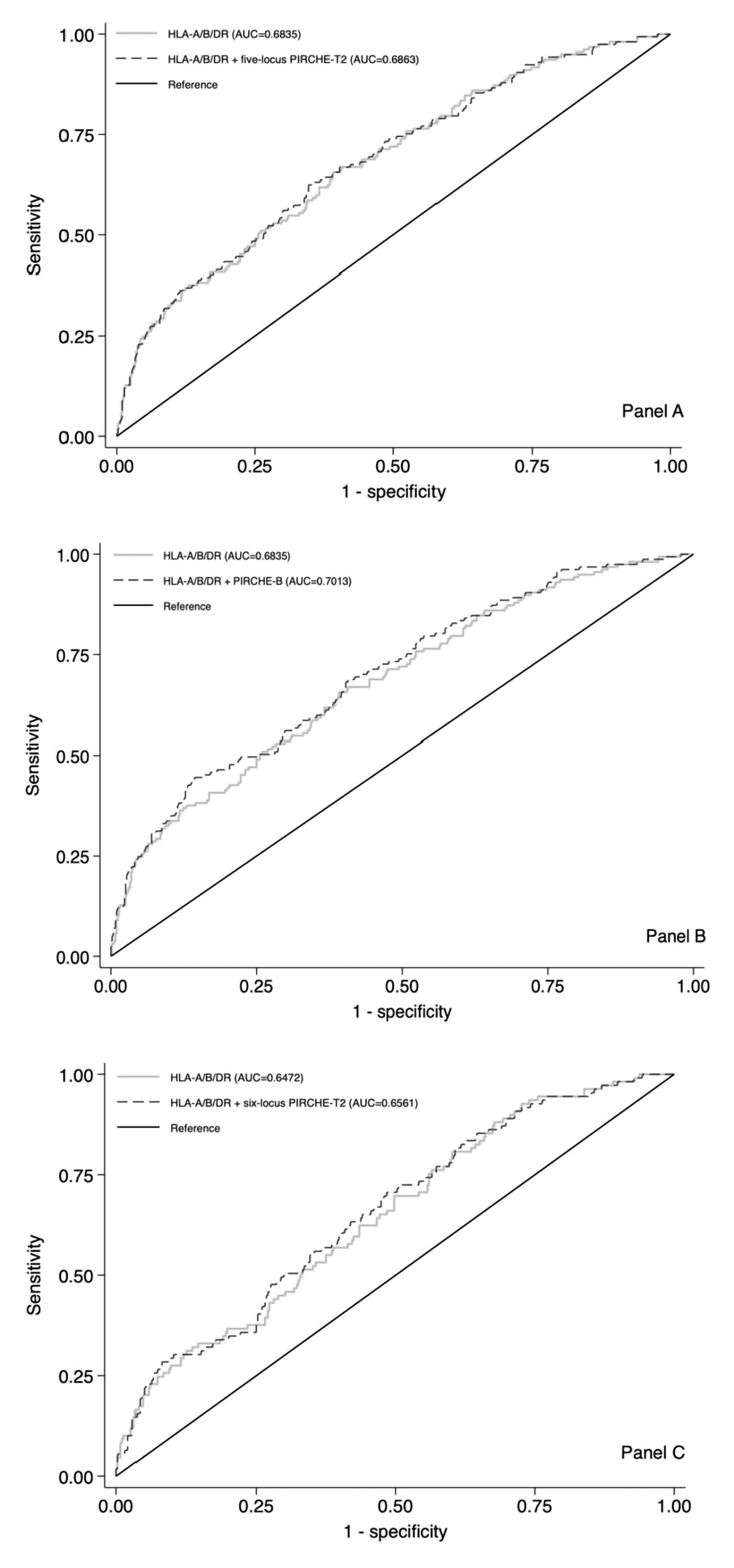
**

**Figure S16:**

**
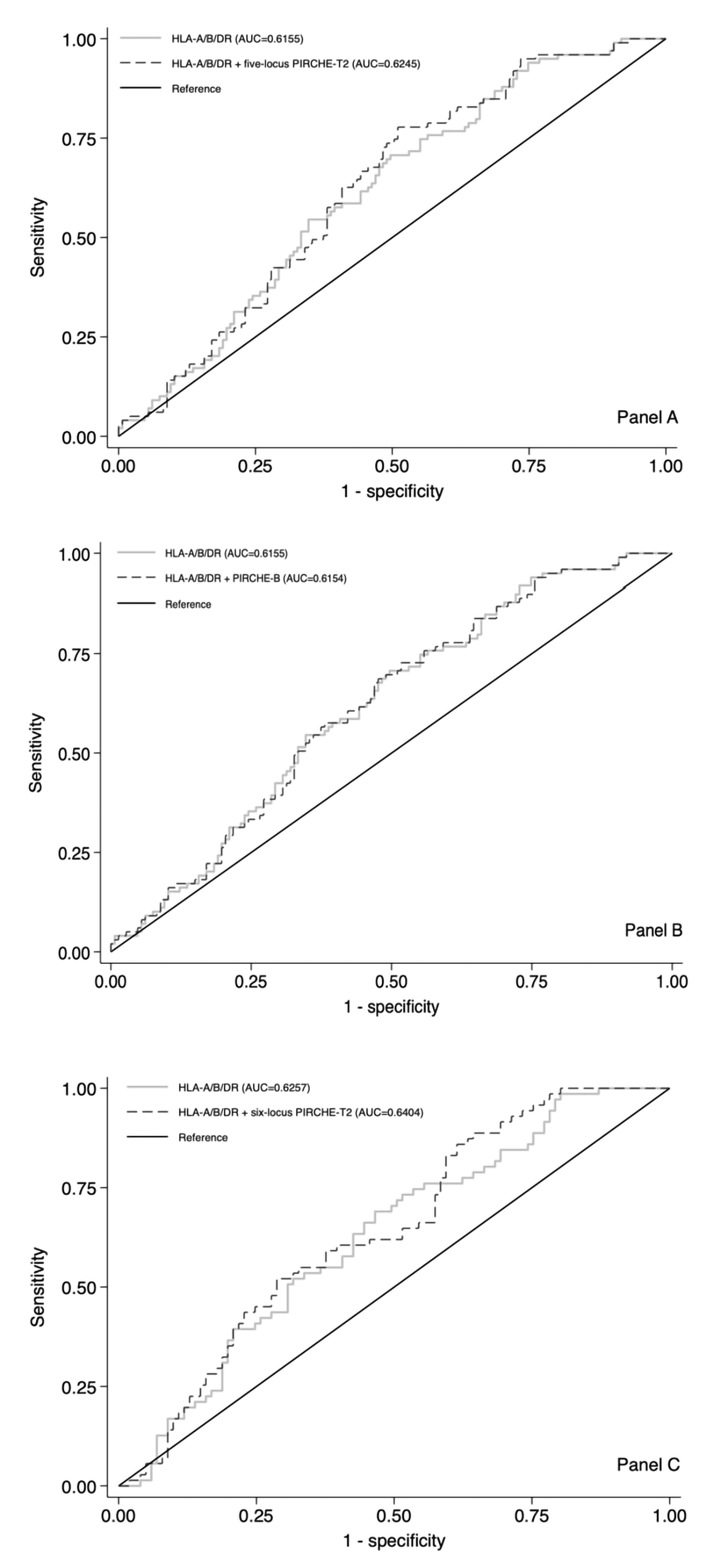
**

**Figure S17:**

**
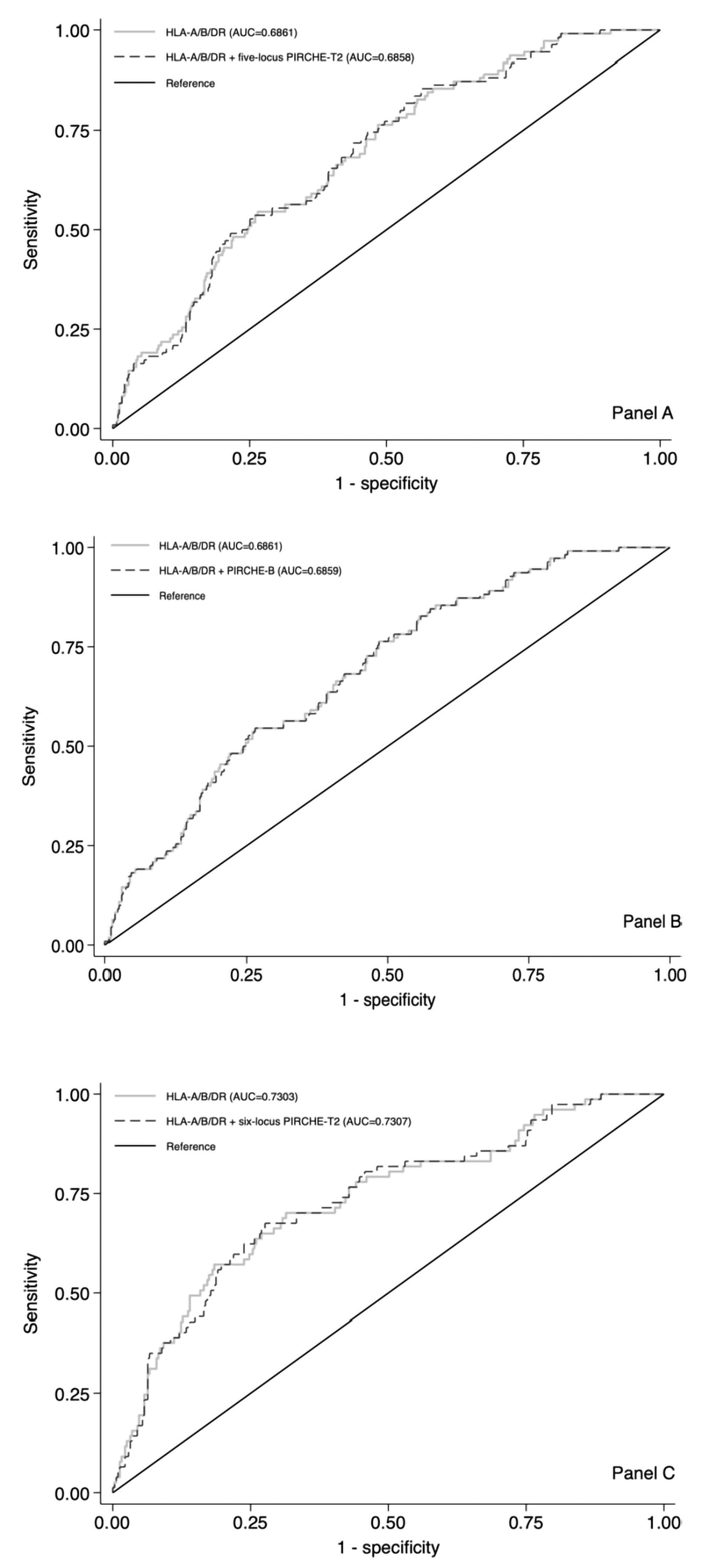
**
